# Research efforts and gaps in the assessment of forest system resilience: A scoping review

**DOI:** 10.1007/s13280-025-02243-4

**Published:** 2025-09-10

**Authors:** Sara Anamaghi, Massoud Behboudian, Mohammad Javad Emami-Skardi, Elisie Kåresdotter, Carla Sofia Santos Ferreira, Georgia Destouni, Lan Wang-Erlandsson, Anna Tengberg, Fabian Stenzel, Ingo Fetzer, Najmeh Mahjouri, Reza Kerachian, Zahra Kalantari

**Affiliations:** 1 Department of Sustainable Development, Environmental Science and Engineering (SEED), KTH Royal Institute of Technology, Stockholm, Sweden; 2 Department of Civil Engineering, Faculty of Technology and Engineering, University of Mazandaran, Mazandaran, Iran; 3 Polytechnic Institute of Coimbra, Applied Research Institute, Coimbra, Portugal; 4 Research Centre for Natural Resources Environment and Society (CERNAS), Polytechnic Institute of Coimbra, Coimbra, Portugal; 5 Department of Physical Geography, Stockholm University, Stockholm, Sweden; 6 Stellenbosch Institute for Advanced Study, Stellenbosch, South Africa; 7 Stockholm Resilience Centre, Stockholm University, Stockholm, Sweden; 8 Bolin Centre for Climate Research, Stockholm University, Stockholm, Sweden; 9 Potsdam Institute for Climate Impact Research, Potsdam, Germany; 10 Lund University Centre for Sustainability Studies (LUCSUS), Lund University, Lund, Sweden; 11 Swedish International Water Institute (SIWI), Stockholm, Sweden; 12 Faculty of Civil Engineering, K. N. Toosi University of Technology, Tehran, Iran; 13 School of Civil Engineering, College of Engineering, University of Tehran, Tehran, Iran

**Keywords:** Ecological resilience, Ecosystem services, Engineering resilience, Forest, Resilience principles, Social-ecological resilience

## Abstract

This study investigates how the seven core resilience principles are integrated into assessments of forest system resilience to natural or human-induced disturbances across engineering, ecological, and social-ecological resilience concepts. Following PRISMA guidelines, a literature search in the Web of Science database using the keywords “resilience”, “forest” and “ecosystem services” yielded 1828 studies, of which 330 met the selection criteria. The most commonly used criterion was diversity, a sub-criterion of “diversity and redundancy”, appearing in 50% of studies. The results indicate that social and governance-related principles, learning and experimentation (7%), participation (11%), and polycentric governance (9%) have not been frequently addressed. Although numerous studies have employed various principles for assessing forest resilience, none have considered all seven principles jointly. This highlights a significant research gap, emphasising the need to quantify these principles in forest systems. Understanding forest-community dynamics is essential for enhancing the long-term resilience and sustainability of both systems.

**Supplementary Information:**

The online version contains supplementary material available at 10.1007/s13280-025-02243-4.

## Introduction

Forests are vital social-ecological systems of Earth, where complex interactions between human societies and the natural environment take place (Biggs et al. 2021). Increasingly, trends in global environmental changes driven by natural or anthropogenic disturbances have rendered forests vulnerable to threats arising from these changes (Ibáñez et al. 2019; Italiano et al. 2023). It is noteworthy that while some natural disturbances, such as wildfires or insect outbreaks, are integral components of forest dynamics, anthropogenic influences like climate change are changing the frequency, magnitude, and spatial extent of these disturbances (Guz and Kulakowski 2020). For instance, climate change has prolonged fire seasons by 18.7% and expanded fire-prone areas by approximately 3% annually between 1979 and 2013 (Jia et al. 2019). Water scarcity, droughts, and pest infestation have also adversely affected larger areas within tropical and temperate forests, in recent decades (Jia et al. 2019). These disturbances can interfere with critical ecosystem functions and the delivery of nearly all types of forest-based ecosystem services, including provisioning (e.g. timber and non-timber forest products (NTFPs), such as fruits and honey), regulating (e.g. carbon sequestration and flood regulation), supporting (e.g. nutrient cycle) and cultural (e.g. cultural heritage and recreation) services. These interferences, in turn, can obstruct the sustainable use of forest resources (Falk et al. 2022) and negatively impact societal well-being (Rani and Sangwan 2022). Hence, understanding and enhancing forest systems’ resilience are critical for maintaining the forest system, its ecosystem services (Seidl et al. 2016) and the long-term sustainability of human societies and the natural environment (Hendrati 2019).

The concept of resilience has its roots in engineering and physical sciences as a part of classical stability theory (Van Meerbeek et al. 2021). It was then applied in the field of ecology by Holling (1973). Since then, this concept has undergone an evolutionary process leading to the generation of diverse definitions and interpretations of resilience for different implementation scopes. As a result of increasing trends in disturbances posing threats to the sustainable development of human communities whose livelihoods are contingent upon forests, a growing body of literature has been devoted to studying forest systems’ resilience in recent decades (Wu and Kim 2013; Cantarello et al. 2017; Sarkki et al. 2017; Islam et al. 2020; Mina et al. 2022; Turner et al. 2022; Nikinmaa et al. 2023; Shumi et al. 2024).

According to Nikinmaa et al. (2020), the three main concepts of resilience in forest studies are engineering resilience, ecological resilience, and social-ecological resilience, with scholars, depending on the nature of their research, focusing on one of them. Among these concepts, social-ecological resilience—the capacity of a social-ecological system (SES) to sustain its structure and functions in the face of internal and external disturbances (e.g. effects of severe droughts on societies and ecosystems, or anthropogenic pressures such as deforestation on ecosystems) by withstanding or absorbing shocks and disturbances, adapting to them, or transforming (Folke 2006; Biggs et al. 2012)—offers the most comprehensive perspective on forest resilience. As forests are SES, the social-ecological resilience concept is best suited to addressing broader environmental challenges and implementing adaptive management strategies within these systems (Mayar et al. 2022). Considering the social aspects when assessing resilience is important because ignoring these facets and the interaction between humans and forests can lead to an incomplete understanding of resilience.

Furthermore, enhancing forest resilience necessitates specific governance arrangements and management policies to meet current and future societal needs (Schlüter et al. 2015). Therefore, besides considering all the ecological facets pertaining to forest resilience, principles relevant to generic policy should also be identified for enhancing the resilience of forest systems and desired ecosystem services in the face of disturbances and ongoing changes. The seven core resilience principles (7PsR) proposed by Biggs et al. (2012) provide a comprehensive framework encompassing all influential dimensions of forest resilience, including ecological, social, and governance factors. Considering these principles for enhancing and assessing resilience in forest systems is crucial, as SES are complex adaptive systems that require a multifaceted approach. These principles are founded on empirical evidence that underscore the interconnectedness of different components of SES. Each principle addresses a specific aspect of SES that contributes to resilience. Maintaining diversity and redundancy (P1) ensures functional backup and response variety. Managing connectivity (P2) supports beneficial interactions across landscapes. Slow variables and feedback (P3) must be closely monitored to prevent abrupt regime shifts. Complex adaptive systems thinking (P4) encourages flexible, multi-scale management in the face of uncertainty. Learning and experimentation (P5) facilitate adaptive responses through continuous knowledge generation, and broadening participation (P6) ensures inclusive, stakeholder-driven decision-making. Lastly, polycentric governance (P7) promotes resilience through multiple, interconnected decision-making centres operating across various levels. More information on these principles and their associated criteria is provided in the supplementary material (Table S1).

In addition, the role of 7PsR in enhancing and assessing resilience has been highlighted in different studies. Gillson et al. (2019) investigated the role of 7PsR in enhancing resilience in fire-prone areas, and Shumi et al. (2024) examined the significance of these principles in the management of woody vegetation and investigated methods to strengthen the resilience of smallholder farming landscapes in the Global South.

On an important note, an issue to be addressed in resilience assessments is answering the two fundamental questions: *resilience of what* and *resilience to what *(Carpenter et al. 2001). In the context of forests as social-ecological systems, the forest-based ecosystem services can provide a basis to frame the first question. Forests provide an array of different ecosystem services, including provisioning, regulating, supporting, and cultural services. These services represent the benefits that forests provide to human well-being and lie at the intersection of ecological and social systems. Changes in forest resilience affect both ecological processes and societal needs, making it crucial to integrate these services in resilience assessments (Seidl et al. 2016). This framing allows reflecting how ecological changes (e.g. loss of biodiversity) translate into impacts on people, and vice versa. Thus, focusing on ecosystem services provides a meaningful lens to integrate both ecological dynamics and social dependencies in resilience assessments. The second question can be answered by considering different disturbances (e.g. drought, land-use change), against which the resilience is assessed.

A review of the literature revealed that despite the widespread adoption of resilience in ecology, a universally applicable framework for resilience assessment remains elusive (Baho et al. 2017). This challenge stems from the complexity of operationalising and quantifying resilience (Albrich et al. 2020). In addition, a significant portion of the literature has not considered both social and ecological aspects when assessing resilience, resulting in an inaccurate perception of the resilience of the region being studied (le Polain de Waroux et al. 2024).

Hence, there is a crucial need to investigate how comprehensively resilience has been assessed in forest systems, particularly in relation to resilience principles. Through a scoping review, this paper provides a broad overview of mapping assessments of forest system resilience in the face of natural or human-induced disturbances and identifies gaps in the resilience assessment. Specifically, we aim to investigate how the 7PsR have been incorporated into forest-related studies, and how they have been quantified. By investigating these aspects, this review offers insights into how resilience in forest studies has been conceptualised and measured, shedding light on the different ways resilience principles have been quantified. This synthesis not only helps to unveil the progress made in developing resilience assessment criteria but also highlights critical conceptual and methodological gaps that need to be addressed to advance resilience research in forest systems.

## Materials and methods

The following subsections of the methodology explain how the relevant studies were identified and the information in them screened. In this review, the Preferred Reporting Items for Systematic reviews and Meta-Analyses extension for Scoping Reviews (PRISMA-ScR) checklist were used to enhance the clarity and transparency of the scoping review (Tricco et al. 2018). For more information regarding the PRISMA checklist, readers are referred to the supplementary material 10.1007/s13280-025-02243-4. Furthermore, data visualisation and validation are expounded in Sects. “Data visualisation” and “Validity”, respectively.

### Search process

The search for relevant studies was conducted via the Web of Science (WoS) academic literature database. For scoping reviews, WoS is one of the commonly used multidisciplinary databases encompassing a substantial collection of the most related representative papers across different research topics, including environmental studies (Rokaya et al. 2018; Dennen et al. 2020; Vigouroux and Destouni 2022; Ma et al. 2023; Zarei and Destouni 2024).

Initially, a set of keywords for locating relevant papers was established by referring to the pertinent and benchmark literature, including Biggs et al. (2012), Nikinmaa et al. (2020) and Nikinmaa et al. (2023). For retrieving papers, the search was conducted on “All Fields” across all available publication years using keywords related to 7PsR via the following command:

(“Resilience” AND “forest” AND “ecosystem services”) AND (“diversity” OR “redundancy” OR “connectivity” OR “slow variables” OR “feedback” OR “adaptive” OR “CAS” OR “learning” OR “experimentation” OR “participation” OR “polycentric governance”).

To ensure the inclusion of all relevant papers, the search was conducted once more with only the following keywords: “forest” AND “resilience” AND “ecosystem services”. The reason for giving consideration to ecosystem services is the coupled nature of forest resilience and ecosystem service concepts. Also, this secondary search served as a validation step to cross-check whether any additional studies, particularly those addressing 7PsR-related concepts without using the specific principle terms, were excluded in the primary search. It is also noteworthy that, for considering all the relevant papers, different word endings for keywords such as forest were captured using the wildcard character, “*”, symbol.

All these keywords together yielded 1828 records for the years 1998 to 2024. This year range was not pre-selected but reflects the full extent of available records retrieved using the defined search keywords, with a cut-off date of March 2024.

### Screening process

**Fig. 1:**
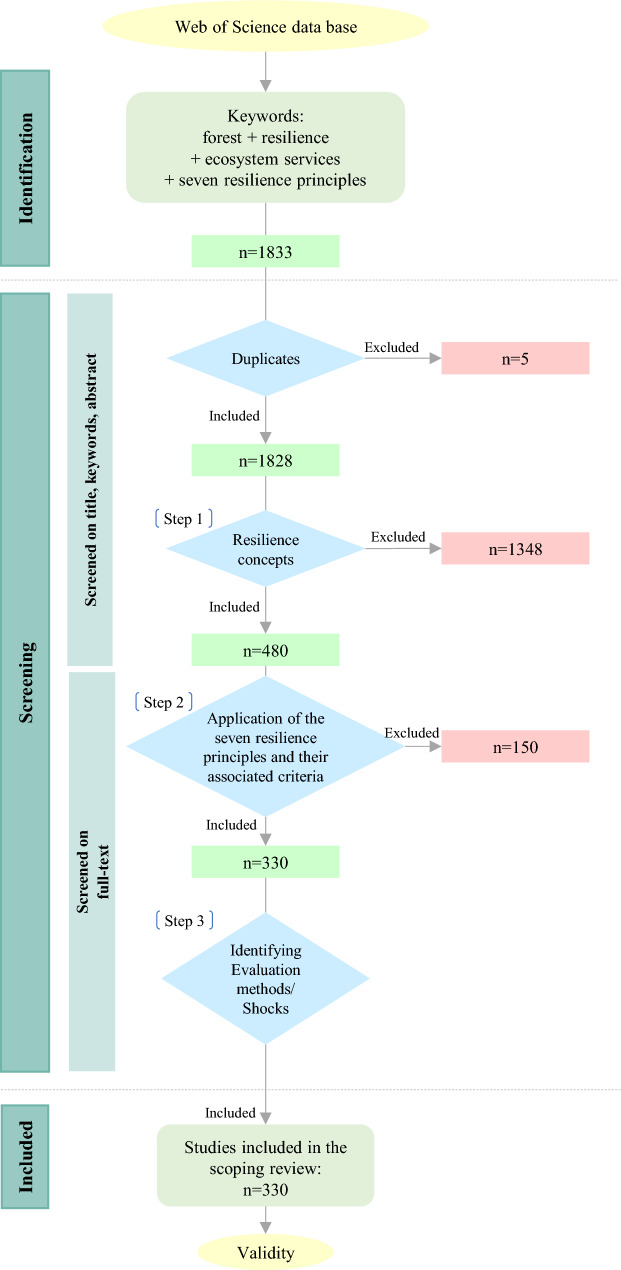
Flowchart for study selection

In the screening process, all the extracted papers were examined using the selection criteria, including resilience concepts, resilience principles and criteria, and evaluation methods. The filtering process of the extracted studies comprised three main steps (Fig. 1):

1. Excluding studies that do not implement engineering, ecological, or social-ecological resilience
2. Filtering papers based on 7PsR and their associated criteria
3. Examining studies for the assessment methods used.

In step one, only those studies that implemented engineering, ecological and social-ecological resilience concepts were selected for further investigations. 480 of the papers met the three selection criteria. In the next step, the studies were examined for the consideration or implementation of resilience principles and their associated criteria. The resilience criteria are defined to enable the quantification of the resilience value. Hence, different criteria and sub-criteria are introduced for each principle. By hierarchically aggregating the values of these resilience criteria, the overall value of resilience can be determined. In this study, we merely considered the resilience criteria that enable the quantification of the seven resilience principles directly. These criteria are proposed based on literature review (Bryant et al. 2019; Albrich et al. 2020; Nikinmaa et al. 2020; Anamaghi et al. 2023; Behboudian et al. 2023, 2024). For more information on resilience principles and their associated criteria, see the Supplementary material (Table S1). While the trade-offs between the resilience principles are important, especially as the principles can reinforce or constrain each other, they were not considered as separate criteria due to the original formulation of the seven resilience principles by Biggs et al. (2012), not considering trade-offs as an explicit principle but rather viewing them as emergent dynamics within and between principles. However, their implications are acknowledged and discussed (see Sect. “Resilience principles in the literature”).

To investigate which resilience principles and criteria have been used in forest resilience studies, all the papers underwent a full-text manual review. This led to the exclusion of 138 papers. At this stage, all irrelevant studies, such as those whose main objectives did not relate to studying and improving forest resilience and resilience mechanisms or that focused on systems other than forests (e.g. coastal, peatland and farmlands), were excluded from the list. Moreover, all studies that did not use any of the listed principles or their related criteria were excluded from the list of records (excluded n = 12). This screening process yielded 330 studies that met all the selection criteria.

In the next step, the assessment methods of the studies were investigated according to the abstract, keywords, and methodology sections. The methods were classified into twelve categories (Table 1). These categories were initially developed by de Vos et al. (2019) and were further refined through a manual review of the relevant papers.

**Table 1:** Methods used in forest resilience assessments (adapted from de Vos et al. (2019))

Name	Explanation
Multi-criteria decision-making (MCDM) methods	MCDM methods use both quantitative and qualitative factors to facilitate the selection of optimal alternatives by systematically reviewing and comparing multiple criteria
Modelling approaches	Studies that utilise ecological/landscape/ecosystem service models
Evidence-based approaches	Studies that conduct interviews, surveys, behavioural analysis, case study analysis, and literature review analysis to describe resilience mechanisms
Conceptual studies	Studies that propose theoretical frameworks for assessing or studying resilience
Spatial mapping and analysis	Studies that use remote sensing and/or geographic information system (GIS)-based approaches either to assess resilience or to map ecosystem services
Scenario analysis	Studies that investigate forest resilience under different climate change or management scenarios
Mixed method	Methods that enable the utilisation of both qualitative and quantitative methods
Social network analysis (SNA) and agent-based modelling	Studies that identify and map influential stakeholders and investigate the impacts of their interaction on forests
Statistical approaches	Studies that employ statistical methods to evaluate resilience criteria or resilience
Experimental and field studies	Studies that conduct experiments in forests or use data from field surveys
Review	–
Other	For example, records of workshops

Finally, for the selected studies, specific disturbances (i.e. resilience to what) that hinder the delivery of ecosystem services in forests, namely climate change, land-use change, fire, drought, insect outbreak, wind, and other disturbances (e.g. deforestation) were identified (adapted from Randhir and Erol (2013)).

### Data visualisation

To enhance the presentation of the study’s results, various visualisation tools were employed. The world map (Figure in Sect. “Key questions of resilience” was generated using ESRI ArcMap software Ver. 10.8.2. The alluvial and radial diagrams (Figures in Sect. “The use of resilience concepts and principles in literature” and Sect. “The use of defined criteria ans sub-criteria for assessing resilience”, respectively) were designed using the Flourish website^1^, and the combined scatter and bar plot (Figure a in the Sect. “Methods for resilience assessment”) was developed with the Python3 Seaborn library Ver. 0.12.2. Additionally, UpSetPlots (Fugure b in Sect. “Methods for resilience assessment” and Figure in Sect. “The co-occurence of core resilience principles” were employed to depict the intersections among various sets of large complex data using MATLAB scripts (MATLAB Ver. 24.2).

### Validity

To ensure the credibility of the results and screening process, a random sample (approximately 10%) of the categorised records was selected and examined by another reviewer. The level of agreement between reviewers can be assessed using Cohen’s kappa coefficient (Cohen 1960). This coefficient measures the accuracy of the review by investigating the discrepancies in the decisions of different reviewers regarding the inclusion of the papers for the review. For more information on this method, readers are referred to Pérez et al. (2020).

In this study, Cohen’s kappa coefficient was determined for 35 randomly selected studies. Given that the diversity criterion was used in 50% of the included studies, this coefficient was computed specifically for the diversity criterion. Among the 35 studies examined by another reviewer, discrepancies in agreement were observed in just three of the studies, resulting in a Cohen’s kappa value of 0.81. Based on Cohen’s kappa categorisation by Landis and Koch (1977), the results of the current review fall into the almost perfect agreement category (Table 2).

**Table 2:** Results of Cohen’s kappa analysis

		Reviewer 1		Sum
		Inclusion	Exclusion	
Reviewer 2	Inclusion	20	2	22
	Exclusion	1	12	13
Sum		21	14	35

As another step to ensure the accuracy of the results, a macro code was used to select the included studies and a thorough full-text manual review was also conducted to cross-check the studies (for more details about the code readers are referred to the Supplementary material S1).

## Results

A total of 1828 peer-reviewed studies on forest resilience were initially identified. The studies were filtered using the different selection criteria, including the resilience concepts (i.e. engineering resilience, ecological resilience, and social-ecological resilience) and resilience principles and their associated criteria. This screening process yielded 330 studies that met all the selection criteria. For the list of selected studies, see the Supplementary material S1. The following subsections elaborate in more detail on the results obtained.

### Key questions of resilience

To address the question of *resilience of what*, 43% of the studies have aimed to investigate the provisioning of different forest-based ecosystem services or have tried to consider them in the resilience assessment process. A list of these ecosystem services is provided in Table 3. Another perspective in answering this question is considering forest health through criteria such as biodiversity and vegetation cover. 32% of the studies have considered one of these criteria (which are discussed in more detail in Sect. “riteria and sub-criteria for assessing resilience”). Furthermore, 4% of the included studies have considered all the factors (ecosystem services, biodiversity, and vegetation cover), and the remaining ones have considered a combination of these factors.

**Table 3:** List of ecosystem services in the studies included in the review

Type	Ecosystem service	Number of papers
Provisioning	Forest products^1^	33
	Clean water	25
Regulating	Soil quality and properties	15
	Soil erosion rate	13
	Habitat air quality	4
	Pest control	11
	Carbon sequestration	27
	Flood regulation	10
	Pollination	6
Supporting	Habitat quality	9
	Nitrogen cycle	14
	Carbon cycle	12
Cultural services	Tourism and recreation	21
	Educational value	9
	Aesthetic inspiration for culture and art	15
	Health and well-being	29
	Cultural practices	28
	Spiritual and religious values	20

^1^Forest products are any products that can be obtained from forests, such as wood, herbs, and honey

**Fig. 2:**
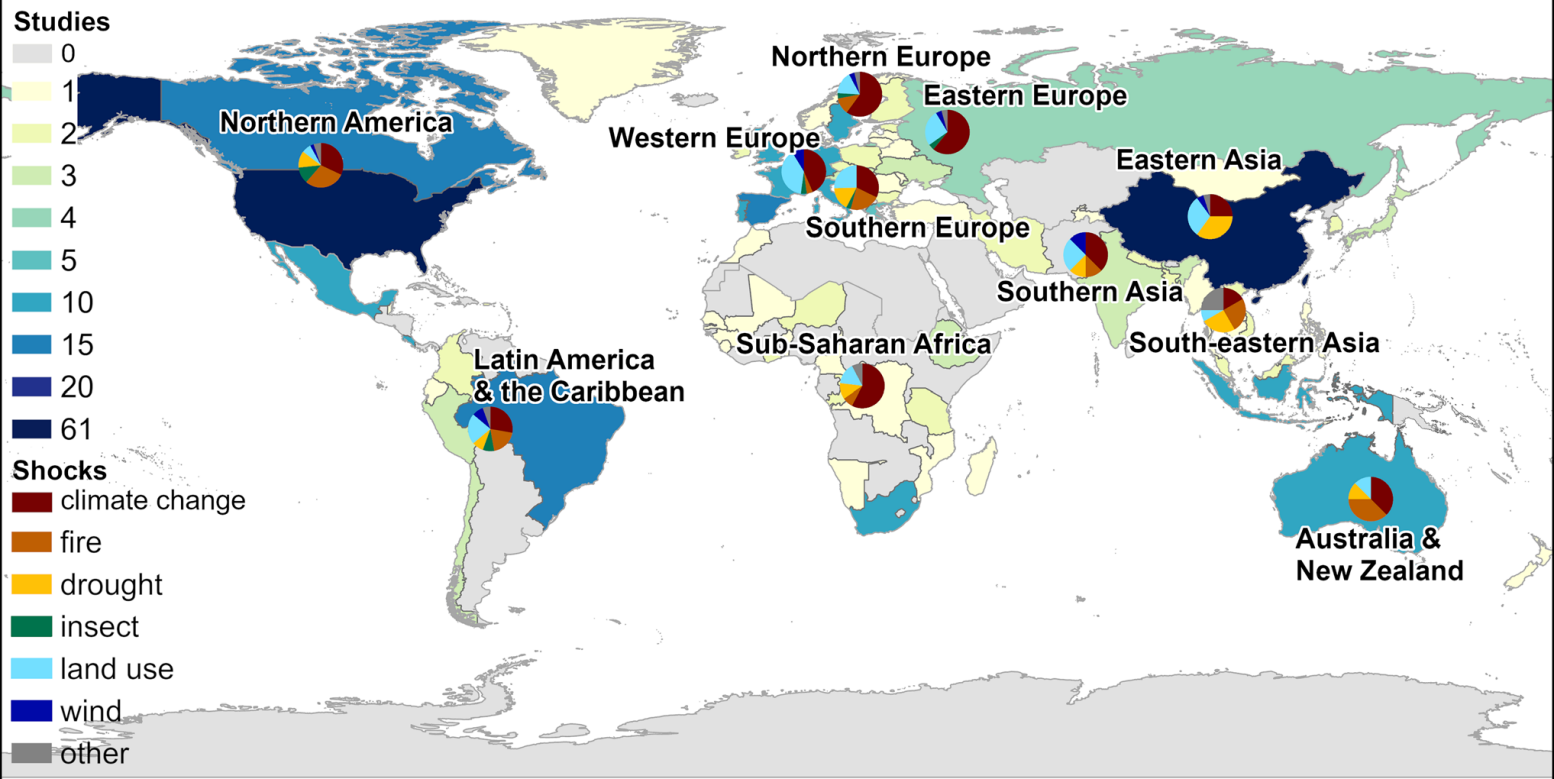
The geographical location of the forests being studied included in this review and the number of disturbances mentioned in these studies per country. A country’s colour represents the number of studies, with darker colours indicating more studies carried out. The pie charts show the total number of researched disturbances in different regions (the division of the regions is based on the United Nations’ M49 subregions)

Studies have addressed the *resilience to what* question by identifying and analysing the disturbances that threaten forest resilience. As illustrated in Fig. 2, climate change is the most frequently studied disturbance across different continents, including Europe, North America, parts of Asia (e.g. India and China) and South America. Resilience against forest fires has been mainly conducted in in the USA, Australia and parts of Africa. Drought has also been considered in studies in North America, Europe, China, and parts of Africa. Insect infestations have received the most research attention in North America, Europe, and certain parts of Asia. Whilst the impacts of land-use changes are important in several regions, they have predominantly been included in studies in parts of Europe and Asia. Wind disturbances are less frequently studied, but studies of some regions, particularly Europe and parts of Asia, have also investigated resilience to this disturbance.

In terms of geographical distribution, most forest resilience studies have been conducted in the USA (61 studies), followed by China (40 studies). There is, also, a high concentration of studies in Europe, parts of South America, and Oceania, while large areas of Africa, Central Asia, and Southeast Asia remain underrepresented.

### The use of resilience concepts and principles in literature

**Fig. 3:**
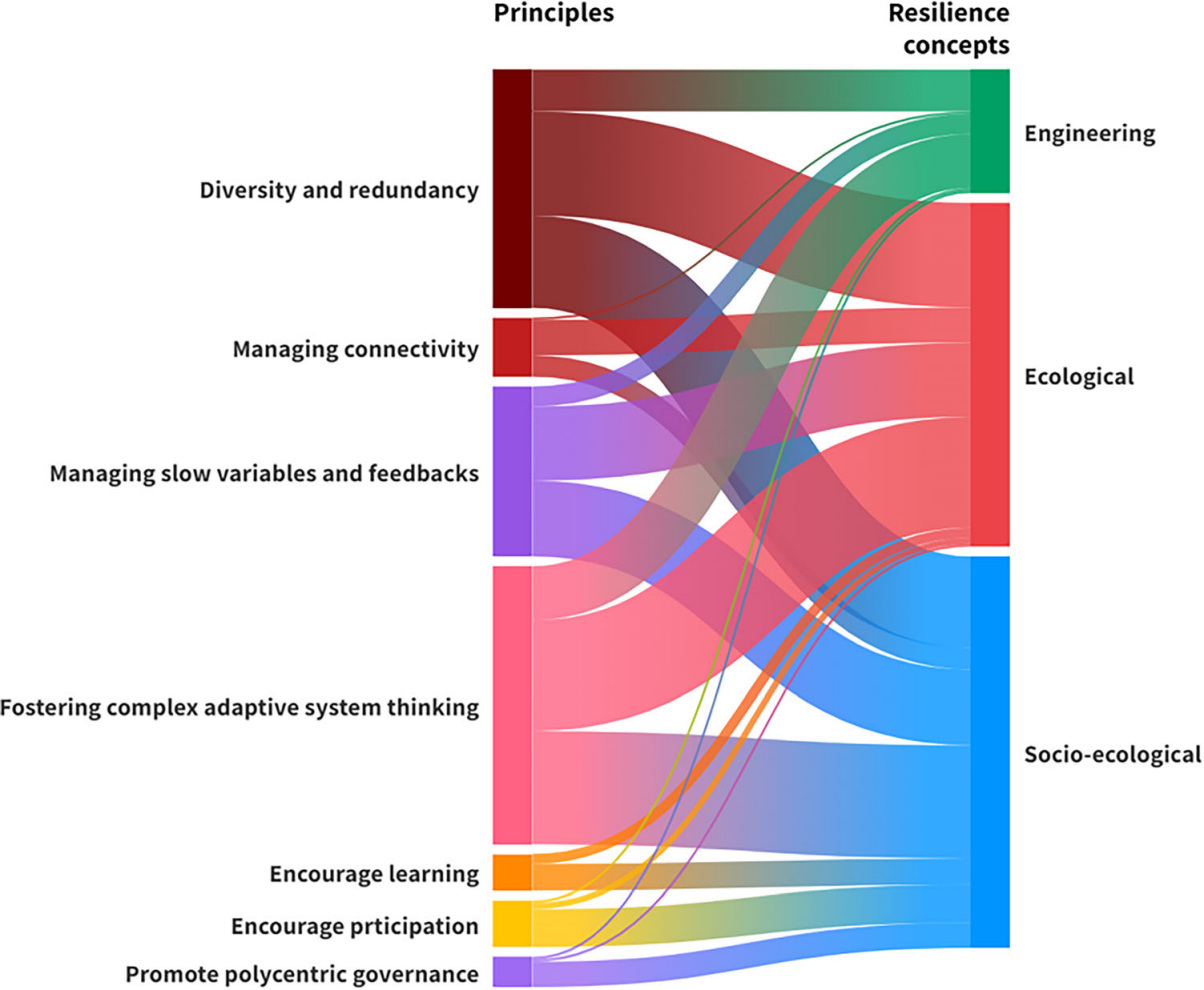
Alluvial diagram depicting the use of core resilience principles across resilience concepts

According to the results, the 7PsR have been implemented in concepts other than social-ecological resilience, including engineering and ecological resilience. The consideration of ecological principles in the two latter concepts is to be expected due to the nature of these concepts (for more details on these concepts readers are referred to the supplementary material S1). However, some studies on ecological and engineering resilience went beyond this and discussed the role of learning, participation, and governance in resilience. This further highlights the comprehensiveness of 7PsR. Figure 3 depicts the application of 7PsR across different resilience concepts in forest systems. On the basis of this data, 15% of the research utilised engineering concepts, 43% employed ecological concepts and 42% incorporated social-ecological concepts.

Engineering resilience uses criteria and indices more closely related to ecological aspects (P1-P4), as it focuses on the system’s recovery time. In this respect, 66%, 4%, 32%, and 86% of the studies on engineering resilience utilised the principles of maintaining diversity and redundancy (P1), managing connectivity (P2), managing slow variables and feedback (P3), and fostering complex adaptive systems thinking (P4), respectively. The reason behind the prevalent usage of P4 in engineering concepts is that other than uncertainties, this principle also addresses the intrinsic characteristics of the system, such as vegetation cover, precipitation, and criteria that pertain to the system’s recovery time (Anamaghi et al. 2023; Behboudian et al. 2023). However, encouraging learning and experimentation (P5) was not considered in engineering resilience, and only 4% of the studies discussed encouraging participation (P6) and promoting polycentric governance (P7). This indicates that the social and policy-related principles (i.e. P5 to P7) are rarely considered when applying the engineering concept.

Ecological resilience, a commonly implemented concept in forest resilience discourse, used all the principles to some extent. Ecological resilience considered principles P1 to P7 in 57%, 19%, 40%, 60%, 5%, 3%, and 2% of the studies, respectively. Like engineering resilience, this concept also emphasises the first four principles more frequently than it does P5, P6, and P7, as the social aspects of the system are overlooked in the ecological concept.

The social-ecological concept incorporates all the principles as it considers social aspects along with environmental aspects. 51%, 12%, 42%, and 63% of the studies that employed the social-ecological concept implemented principles P1 to P4. Moreover, 15%, 21%, and 14% of the studies assessed or discussed the role of learning and experimentation, participation, and polycentric governance, respectively, in enhancing the resilience of SES. Although overall, social principles and their criteria are still less represented than the other principles, within the social-ecological concept these social and policy-related aspects are considered more frequently than in the engineering or ecological concepts. Among all the studies, only two investigated all the 7PsR together when examining the resilience of forest systems, both of which are review papers (Gillson et al. 2019; Shumi et al. 2024). This indicates a significant gap in the evaluation of forest resilience via 7PsR.

Hence, the results indicate that much closer attention should be paid to social aspects (P5-P7) when assessing forest resilience. Notably, Fig. 3 merely illustrates the use of the principles in the different concepts, irrespective of the co-occurrence of the principles. This issue is elaborated in more depth in Sect. “The co-occurrence of core resilience principles”.

### The use of defined criteria and sub-criteria for assessing resilience

**Fig. 4:**
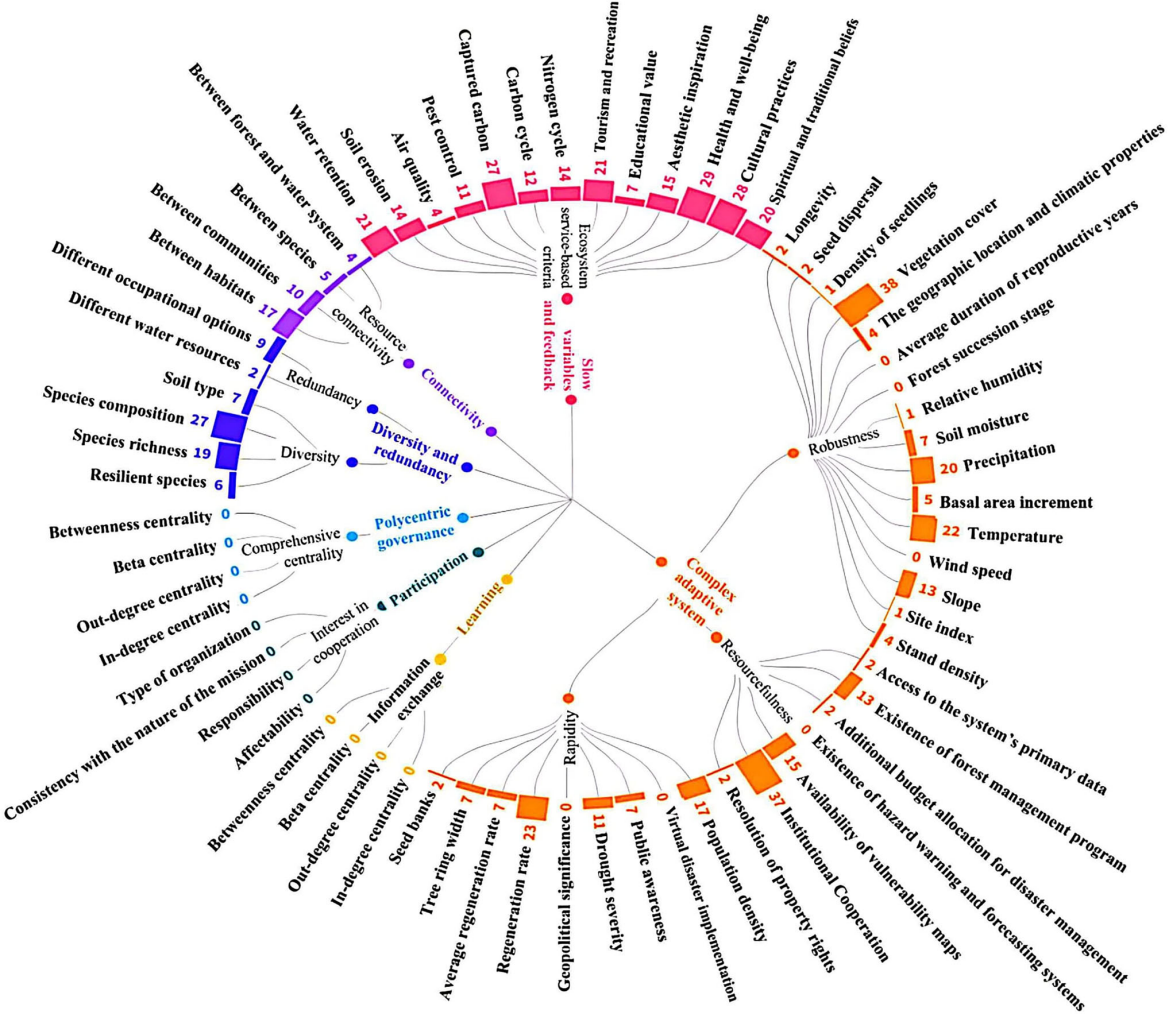
Number of publications that have utilised each resilience criterion and sub-criterion for all 7PsR, which are indicated by a different colour

To quantify resilience according to the details of the 7PsR, it is necessary to define various criteria and sub-criteria for each principle. The criteria for evaluating the resilience principles, extracted from the literature (Chapin et al. 2004; Newton 2011; Folke et al. 2016; Albrich et al. 2020; Moreno-Fernández et al. 2021; Nikinmaa et al. 2023), are depicted in Fig. 4. Different studies have used an array of these criteria and sub-criteria to evaluate 7PsR, and the most frequently used criteria for each principle are given below. According to Fig. 4, the majority of the literature focuses on defining criteria and quantifying the first four principles. In contrast, principles 5, 6, and 7 are not fully addressed. For example, social network analysis (SNA)-based criteria, such as various centrality^2^ criteria, are not taken into account.

Among the different criteria proposed to assess the first principle (P1), species richness and composition criteria have been used more frequently than other diversity/redundancy-based criteria to assess diversity and redundancy. For the connectivity principle (P2), the primary indicator has been the criterion about connectivity between the habitats, which investigates the existence of habitat corridors.

Examining the results obtained for the sub-criteria of the third principle (P3) revealed that the health and well-being criterion has been implemented more frequently. The principle of fostering complex adaptive systems (CAS) thinking (P4) consists of three main criteria of robustness (which refers to the intrinsic characteristics of the system that enable it to withstand shocks and disturbances), resourcefulness (the capacity of the system to mobilise resources in times of disturbance), and rapidity (the ability of the system to recover after facing disturbance) (Karamouz et al. 2018; Anamaghi et al. 2023; Behboudian et al. 2023, 2024). Vegetation cover, institutional cooperation and the average regeneration rate come top, respectively, among the sub-criteria of robustness, resourcefulness, and rapidity. Also, among all the criteria proposed for fostering CAS thinking based on previous work on resilience frameworks, some, such as the forest succession stage and the presence of hazard warning and forecasting systems, remain unexplored in the literature.

Another point worth mentioning is that most studies focusing on learning and experimentation (P5), participation (P6), and polycentric governance (P7) principles have not used stakeholder analysis and social network analysis and their relevant criteria, including centrality-based criteria as shown in Fig. 4. The studies aiming to to quantify these principles have examined the perspectives of various stakeholders on these principles qualitatively. Therefore, a significant gap exists in the assessment of forest resilience in terms of social principles and their quantification.

### Methods for resilience assessment

**Fig. 5:**
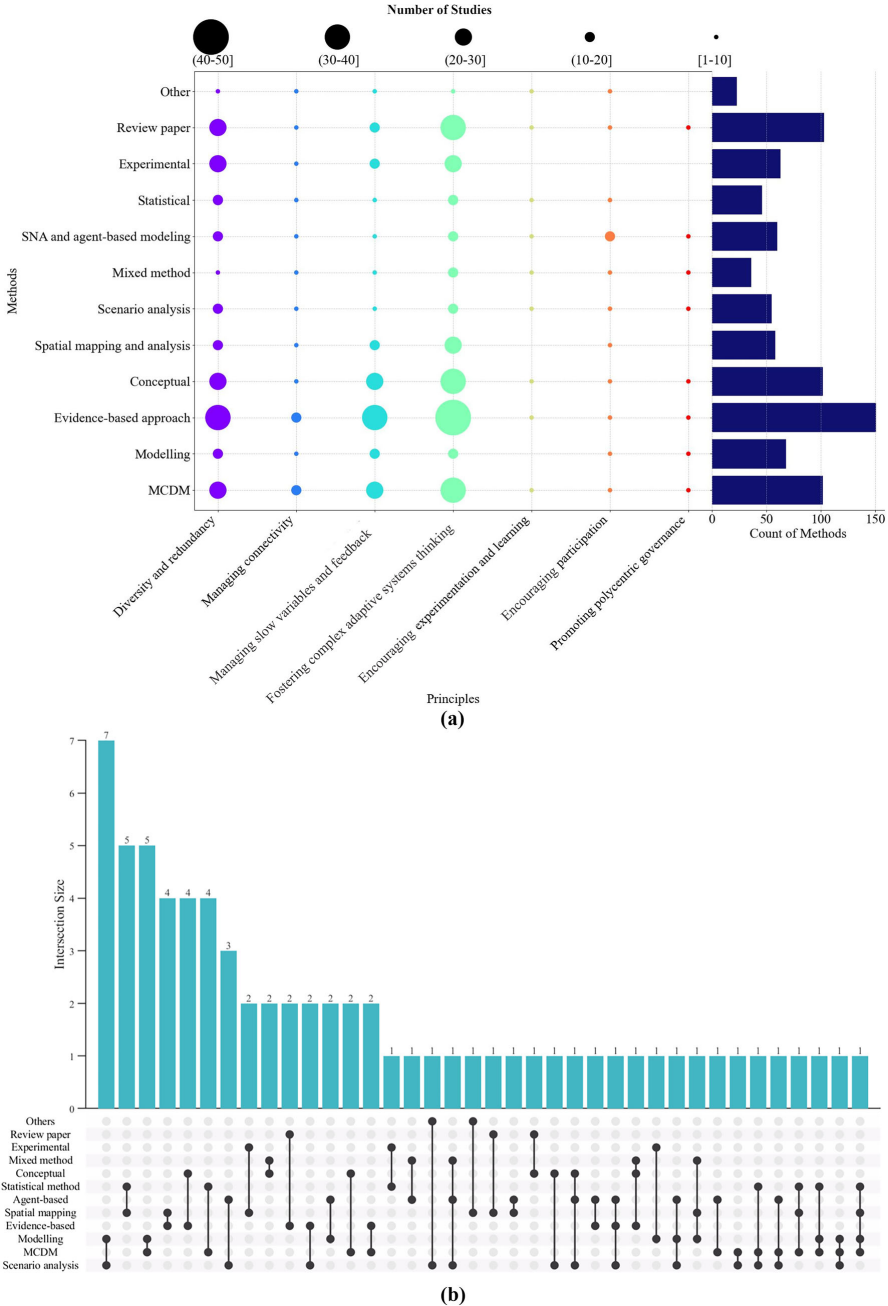
**a** Methods utilised to evaluate the resilience principles and their respective frequency in the included studies and **b** the number of studies that have used a combination of methods to assess forest resilience

As mentioned in Sect. “Screening process”, 12 different methods for resilience studies were identified. Figure 5a reveals that a significant body of literature (44%) has implemented an evidence-based approach. This method is a qualitative approach that uses and interprets existing evidence in the literature, historical records and historical knowledge to determine the mechanisms, policies, and practices that influence resilience. The second most prevalent method is the multi-criteria decision-making (MCDM) method. According to the results, species diversity and vegetation cover are used in 47% and 19% of the studies, making them the most common indices used in this approach.

When examining how each resilience principle was assessed using specific methods, it is clear that the evidence-based and MCDM methods consistently ranked first or second among the various methods, except for P5 to P7, which pertain to social principles. Encouraging learning and experimentation (P5), encouraging participation (P6), and promoting polycentric governance (P7) principles have mainly been assessed through agent-based modelling. While most studies using agent-based modelling have focused either on examining stakeholder perceptions and behavioural patterns regarding various ecosystem services or on collaborating with stakeholders to co-develop management scenarios, they have often overlooked the analysis of stakeholders and their networks. This type of analysis, which explores the interactions, impacts and decision-making processes between stakeholders using criteria such as density or centrality, is distinct from the approaches mentioned above, as illustrated in Fig. 5a. Furthermore, approximately 30% of the literature on forest resilience included in this review is devoted to review papers and conceptual studies.

Figure 5b depicts the studies that implemented a combination of different methods for evaluating resilience, and the methods that have been used together (i.e. 21% of all the included studies used a combination of different methods). The MCDM method has been widely adopted in conjunction with other methodologies, such as modelling, statistical approaches, and scenario analysis. Scenario analysis and modelling have also been employed in combination with various methods, such as statistical methods and agent-based methods.

### The co-occurrence of core resilience principles

**Fig. 6:**
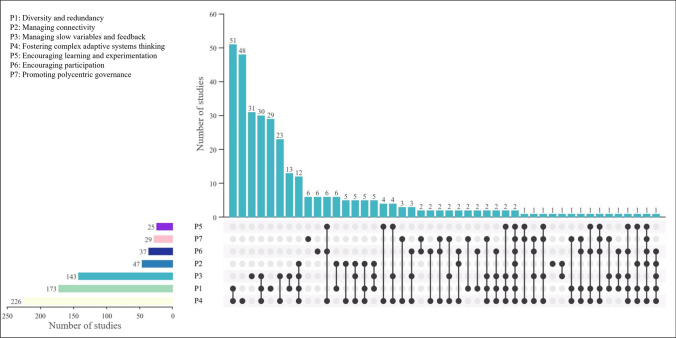
Plot representing the co-occurrence and usage of the resilience principles in the reviewed studies

As one of the main aims of this review, the co-occurrence and implementation of different resilience principles were scrutinised and are presented in Fig. 6. In this regard, fostering complex adaptive systems thinking (P4) has the highest record of implementation in different studies. However, not all related studies have attempted to consider different aspects of this principle, including all spatial and temporal aspects of the system as well as uncertainties associated with the system in the assessment process. If the studies have only used one criterion (e.g. robustness, rapidity) or sub-criterion of this principle (e.g. vegetation cover), the study has been considered to be using P4. As most studies have attempted to assess resilience through intrinsic characteristics of the forest or assess the forest recovery rate, the usage rate of P4 is higher than those of the other principles.

Furthermore, P4 has been considered in combination with almost all other principles. P4 and P1 have the highest record of co-occurrence. Delving deeper into the methods the studies have used to investigate P1 and P4 jointly reveals that experimental and fieldwork methods have been used particularly frequently (in approximately 50% of the studies). This aligns with the nature of these principles, as both refer to the ecological characteristics of forests, which can be scrutinised by collecting data through experimentation and field visits. Spatial mapping (e.g. using remote sensing data) has also been widely implemented to quantify these principles.

The joint use of P1, P3, and P4 ranks second in the co-occurrence of the principles. For the assessment of these principles, evidence-base methods were mostly used (in 20% of cases). The same number of studies, 20%, pertain to review or conceptual papers outlining the importance of the joint use of these principles in resilience assessment.

Social principles (P5-P7) appear in fewer studies overall, but still, they contribute to resilience discussion, especially via evidence-based methods such as interviews or agent-based modelling methods, where the main focus is on exploring the perception of a number of stakeholders about mechanisms contributing to resilience. Notably, only two studies have considered all 7PsR, both of which are review papers (i.e. Gillson et al. 2019; Shumi et al. 2024), which means that 7PsR have not been taken into account in the quantitative assessment of the social-ecological resilience of forests.

## Discussion

Despite the comprehensiveness of social-ecological resilience and introduction of 7PsR as a comprehensive framework to study resilience (Biggs et al. 2012), no studies have utilised all of these principles jointly for resilience assessment. Some studies have combined several principles effectively (Nikinmaa et al. 2023). However, methods for quantifying or applying these principles to assess forest resilience are still lacking. Thus, a gap exists in the quantification and use of 7PsR in forest resilience assessments.

One of the main issues in resilience research is inconsistent terminology. Here, only studies that addressed engineering, ecological, and social-ecological resilience were investigated as these concepts are widely used in forest resilience research (Nikinmaa et al. 2020). Despite using various keywords to filter and identify relevant studies, some may have been excluded because of differing terminology. Moreover, trade-offs between resilience principles were not analysed as an independent category. However, key trade-offs emerging from principles interactions are further discussed in the following subsection. Also, as the number of studies investigating forest resilience in the USA and China is higher than in the rest of the world, the results can be biased towards these countries.

### Resilience principles in the literature

**Fig. 7:**
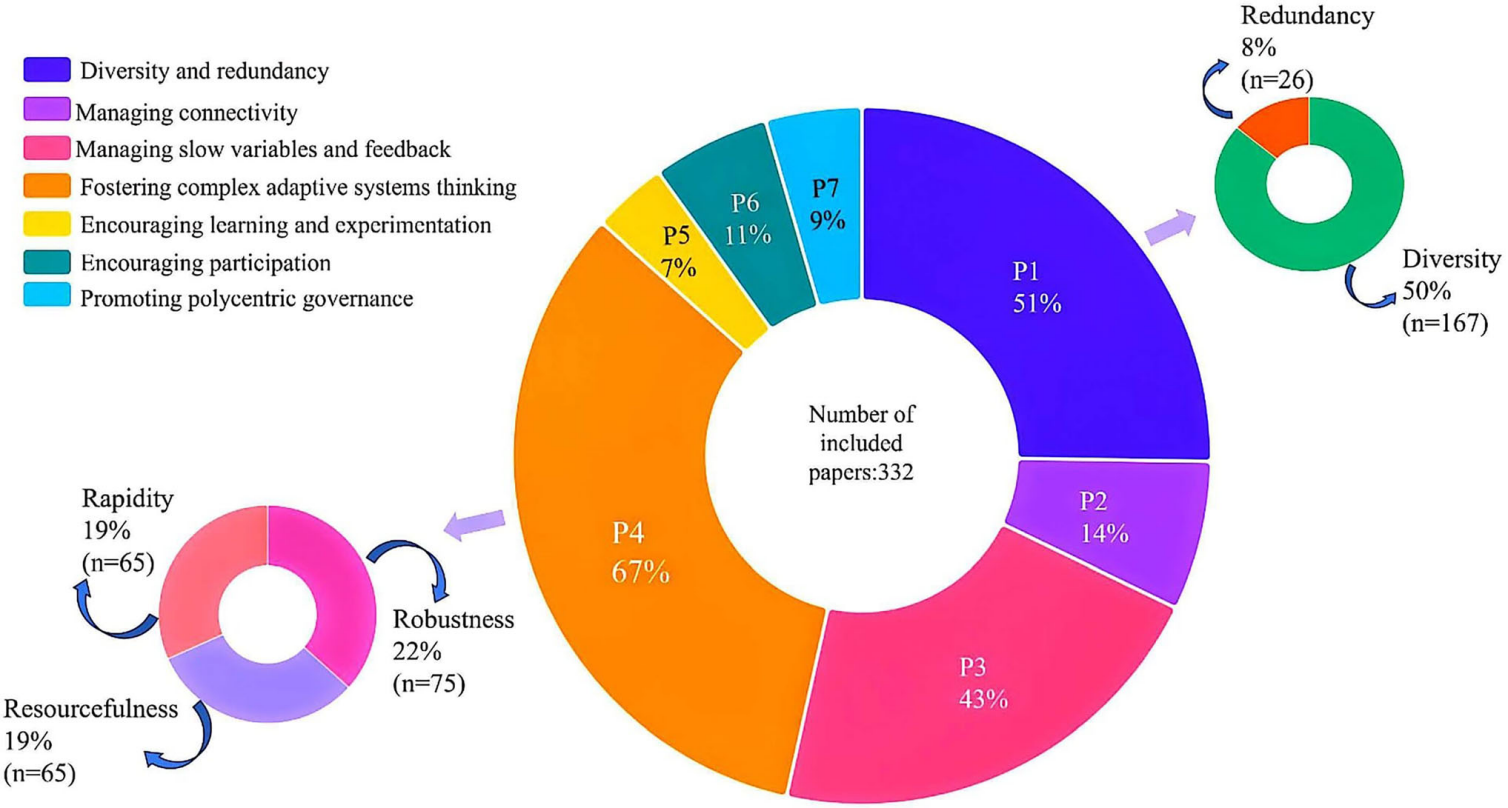
Implementation of resilience principles and the criteria of the most frequently used principles in the included studies

The most commonly used principle is fostering complex adaptive systems (CAS) thinking (P4), emerging in more than 67% of the included studies, followed by maintaining diversity and redundancy (P1), which appears in 51% of the papers analysed in this review. However, a closer look reveals that the overall use of diversity criteria far exceeds the individual use of any P4 criteria or the instances in which all P4 criteria were applied simultaneously (i.e. 3%) (Fig. 7). Therefore, not all the essential requirements of P4, which emphasises comprehensive management that includes all relevant temporal and spatial scales and considers associated uncertainty, were considered in all of the cases.

Social-related principles (i.e. P5, P6, and P7) are rarely included in peer-reviewed studies (7%, 11%, and 9%, respectively), indicating a gap regarding the inclusion of key stakeholders, their responsibilities and their beliefs in resilience research alongside environmental criteria and indices.

Furthermore, a significant number of studies have implemented the principles individually or just two or three of them jointly. Upon closer examination, it becomes clear that these studies have only partially addressed these principles (depicted in Fig. 4), meaning they have not fully incorporated the intent or application of the principles in the resilience assessment. Baird et al. (2024), who investigated the literature to find evidence of operationalising 7PsR, reported that out of more than 750 identified papers, only 23 have tried to operationalise the 7PsR, with only seven studies operationalizing all seven principles simultaneously. According to their results, these principles were used to study resilience in focal resource systems other than forests (e.g. watersheds, fisheries, cities, and freshwater systems). Hence, there is a significant gap in incorporating all the resilience principles for evaluating the resilience of forest systems.

In addition, it is important to note that these resilience principles are deeply intertwined, meaning that studying them in isolation is unlikely to provide a comprehensive tool for resilience assessment. These principles influence and reinforce one another. Diversity and redundancy can enhance resilience by providing multiple pathways for recovery and adaptation. Connectivity can facilitate the exchange of resources and information among diverse components, further supporting diversity (P1 and P2). Furthermore, when diverse perspectives are incorporated, adaptive learning becomes more effective, increasing the system’s ability to cope with change (P1 and P5). Participation fosters trust and inclusivity, which are vital for collaborative learning and experimentation (P5 and P6). Polycentric governance—where multiple authorities manage ecosystems at different scales—creates more opportunities for stakeholder participation. This, in turn, improves governance by integrating local knowledge and concerns (P5, P6, and P7). Recognising SES as complex adaptive systems highlights the need for continuous learning and flexible management. This approach also supports experimentation, which is essential for adapting to dynamic and uncertain conditions (P4 and P5). Monitoring slow variables helps detect gradual changes before they become crises. Polycentric governance improves the management of slow variables by allowing decision-making at different levels. Local authorities can monitor changes at smaller scales, while national or international bodies address broader systemic challenges (P3 and P7).

This interconnectedness of principles can also create trade-offs, which need to be carefully managed. Excessive connectivity may lead to homogenisation and facilitate the rapid spread of disturbances. Hence, a balance between diversity and connectivity should be maintained (P1 and P2). Although participation can increase learning opportunities, excessive inclusivity can slow decision-making processes, potentially delaying urgent adaptive responses (P6). This is also true about polycentricity. Redundant hierarchical governing structures and entities can create bureaucratic complexity, leading to coordination challenges or conflicting policies, which can further interfere with implementing long-term strategies for managing slow variables.

Furthermore, there is an overlap between the indicators used across the three concepts of resilience, namely engineering, ecological, and social-ecological (Fig. 3). This is logical since these concepts are nested within each other, and they form conceptual basis for one another (Nikinmaa et al. 2020). Engineering resilience, focusing on the system recovery, shares a conceptual basis with ecological resilience, which also analyses system response and considers the possibility of alternative stable states and regime shifts. Moreover, ecological resilience and social-ecological resilience have the same theoretical foundation regarding the ecological process, while the latter considers the impacts of social, institutional, and governmental factors on resilience. For more information on resilience concepts readers are referred to supplementary material (Note 10.1007/s13280-025-02243-4). As shown in Fig. 3, principles such as P1, P3, and P4 are commonly applied across all three resilience concepts, reflecting these conceptual linkages and overlaps in practice.

### Forest resilience evaluation methods

As resilience is a multidimensional concept, its assessment requires methods that capture the ecological, social, governance, and economic facets of resilience. In this review, excluding review papers, eleven methods for assessing forest resilience were identified, including the MCDM method, modelling, evidence-based approaches, conceptual studies, spatial mapping and analysis approaches, scenario analysis, mixed-methods, agent-based modelling, statistical approaches, experimental and field studies and others (e.g. workshop reports). The predominant reliance on qualitative approaches, particularly evidence-based approaches (45% of the reviewed studies), reveals the strong emphasis on assessing and understanding resilience mechanisms based on empirical findings from previous research, case studies, and practical experiences (Kelly et al. 2015; Fedele et al. 2017; Messier et al. 2022). Although these approaches provide insights into resilience patterns, their descriptive nature hinders their ability to predict future trends, restricting the provision of actionable insights under future environmental conditions (Kharrazi et al. 2016). In contrast, the MCDM method, the second most commonly used method, offers a robust framework to consider and assess multiple resilience criteria and principles quantitatively; however, it is often used to integrate environmental and ecological criteria, neglecting governance and social criteria critical for holistic resilience assessment (Sarkki et al. 2017; Bryant et al. 2019; Nikinmaa et al. 2023).

A significant body of literature (approximately 30%) was devoted to conceptual and review papers, reflecting the dynamic and evolving nature of forest resilience research. This also underscores the lack of a comprehensive and unified framework that enables the consistent assessment and operationalization of resilience across different forest ecosystems and governance contexts.

The small number of studies (almost 20%) that integrated multiple methods for assessments of forest resilience is another point to highlight. For example, scenario analysis is employed alongside other methods, such as modelling, spatial mapping, and MCDM methods (e.g. Dymond et al. (2014); Riva et al. (2018); Wyatt et al. (2021)). Although the combined use of scenario analysis can provide long-term projections, enable decision-makers to evaluate different policy and management strategies and explore possible future trajectories, their integration with other approaches remains scarce (less than 7%).

Furthermore, there are other drawbacks to the heavy reliance on single-method approaches. Although qualitative methods offer a good comprehension of existing patterns, they lack quantifiability and predictive power, making it difficult to assess resilience under changing socio-economic and environmental future conditions. In contrast, quantitative methods such as MCDM, statistical analysis, and modelling offer insights into the future, yet they fail to capture social and governance dimensions. All these can hinder the understanding necessary to address forest resilience issues across various spatial and temporal scales (Filatova et al. 2013; Reyer et al. 2015).

Therefore, given the increasing complexity of forest resilience challenges, there is a need for more studies employing multi-method approaches and combining qualitative and quantitative methods to increase the robustness of forest resilience assessments while accounting for the associated uncertainties, an issue that has also been highlighted by Reyer et al. (2015). Myllyviita et al. (2014) also highlighted the benefits of using a combination of quantitative and qualitative approaches, especially in natural resource planning.

## Conclusion

Since 2010, research on forest resilience has seen a notable increase, with many studies concentrated in the USA and China. However, despite this growing body of knowledge, global forest loss continues at an alarming rate, around 4.7 million hectares per year, highlighting a significant gap between research and practical implementation. This disconnect is compounded by inadequate monitoring systems and the escalating impacts of climate change. The review of resilience principles reveals that complex adaptive systems thinking (P4) and managing diversity and redundancy (P1) are the most investigated principles, reflecting a focus on system stability and ecological processes. Conversely, social principles such as encouraging learning, participation, and polycentric governance (P5, P6, and P7) remain underrepresented, with only a minority of studies exploring these aspects. Even when stakeholders are involved, their roles are limited mostly to perception surveys or scenario testing, indicating that human–environment interactions and institutional arrangements require more attention in resilience assessments.

From a methodological perspective, nearly half of the studies employ evidence-based approaches that provide valuable qualitative insights but fall short of offering predictive capabilities essential for strategic forest management. The use of multi-criteria decision-making (MCDM) methods, the second most common method, often emphasises ecological criteria and tends to neglect social considerations, limiting a comprehensive understanding of resilience. Few studies adopt multi-method approaches that integrate both qualitative and quantitative perspectives, which constrain their scalability and policy relevance. The absence of research explicitly including all seven resilience principles (7PsR) emphasises the need for future efforts to adopt a holistic, SES-based framework, especially since forests are intrinsically coupled social-ecological systems. Strengthening resilience assessments with mixed-methods and uncertainty analysis can bridge the gap between science and practice, enabling more effective policies and management strategies to combat ongoing forest degradation and build resilient forests for the future.

## Supplementary Information

Below is the link to the electronic supplementary material.

Supplementary file1 (PDF 858 kb)

## Data Availability

The categorised list of all included studies can be downloaded in Zenodo^3^.

## Footnotes

1
https://flourish.studio/.

2 Centrality, in general, refers to the number of stakeholders adjacent to a specific stakeholder. Centrality measures are used to identify influential stakeholders in the network (Ahmadi et al. 2019; Ahmadi et al. 2020).

## References

[CR1] Ahmadi, A., R. Kerachian, R. Rahimi, and M. J. E. Skardi. 2019. Comparing and combining social network analysis and stakeholder analysis for natural resource governance. *Environmental Development* 32: 100451.

[CR2] Ahmadi, A., R. Kerachian, M. J. E. Skardi, and A. Abdolhay. 2020. A stakeholder-based decision support system to manage water resources. *Journal of Hydrology* 589: 125138.

[CR3] Albrich, K., W. Rammer, M. G. Turner, Z. Ratajczak, K. H. Braziunas, W. D. Hansen, and R. Seidl. 2020. Simulating forest resilience: A review. *Global Ecology and Biogeography* 29: 2082–2096. 33380902 10.1111/geb.13197 PMC7756463

[CR4] Anamaghi, S., M. Behboudian, N. Mahjouri, and R. Kerachian. 2023. A resilience-based framework for evaluating the carrying capacity of water and environmental resources under the climate change. *Science of the Total Environment* 902: 165986. 37536587 10.1016/j.scitotenv.2023.165986

[CR5] Baho, D. L., C. R. Allen, A. S. Garmestani, H. B. Fried-Petersen, S. E. Renes, L. H. Gunderson, and D. G. Angeler. 2017. A quantitative framework for assessing ecological resilience. *Ecology and Society: A Journal of Integrative Science for Resilience and Sustainability* 22: 1. 10.5751/ES-09427-220317 PMC5759782 29333174

[CR6] Baird, J., J. L. Blythe, C. Murgu, and R. Plummer. 2024. A scoping review of how the seven principles for building social-ecological resilience have been operationalized. *Ecology and Society *29.

[CR7] Behboudian, M., S. Anamaghi, N. Mahjouri, and R. Kerachian. 2023. Enhancing the resilience of ecosystem services under extreme events in socio-hydrological systems: A spatio-temporal analysis. *Journal of Cleaner Production* 397: 136437.

[CR8] Behboudian, M., S. Anamaghi, R. Kerachian, and Z. Kalantari. 2024. Comparison of three group decision-making frameworks for evaluating resilience time series of water resources systems under uncertainty. *Ecological Indicators* 158: 111269.

[CR9] Biggs, R., M. Schlüter, D. Biggs, E. L. Bohensky, S. BurnSilver, G. Cundill, V. Dakos, T. M. Daw, et al. 2012. Toward principles for enhancing the resilience of ecosystem services. *Annual Review of Environment and Resources* 37: 421–448.

[CR10] Biggs, R., H. Clements, A. de Vos, C. Folke, A. Manyani, K. Maciejewski, B. Martín-López, R. Preiser, et al. 2021. What are social-ecological systems and social-ecological systems research? In *The Routledge handbook of research methods for social-ecological systems*, 3–26. London: Routledge.

[CR11] Bryant, T., K. Waring, A. S. Meador, and J. B. Bradford. 2019. A Framework for quantifying resilience to forest disturbance. *Frontiers in Forests and Global Change* 2: 56.

[CR12] Cantarello, E., A. C. Newton, P. A. Martin, P. M. Evans, A. Gosal, and M. S. Lucash. 2017. Quantifying resilience of multiple ecosystem services and biodiversity in a temperate forest landscape. *Ecology and Evolution* 7: 9661–9675. 29187998 10.1002/ece3.3491 PMC5696413

[CR13] Carpenter, S., B. Walker, J. M. Anderies, and N. Abel. 2001. From metaphor to measurement: Resilience of what to what? *Ecosystems* 4: 765–781.

[CR14] Chapin, F. S., G. Peterson, F. I. K. R. E. T. Berkes, T. V. Callaghan, P. Angelstam, M. Apps, C. Beier, Y. Bergeron, et al. 2004. Resilience and vulnerability of northern regions to social and environmental change. *Ambio* 33: 344–349. 10.1579/0044-7447-33.6.344. 10.1579/0044-7447-33.6.344 15387072

[CR15] Cohen, J. 1960. A coefficient of agreement for nominal scales. *Educational and Psychological Measurement* 20: 37–46.

[CR16] de Vos, A., R. Biggs, and R. Preiser. 2019. Methods for understanding social-ecological systems: A review of place-based studies [Review]. *Ecology and Society* 24: 19.

[CR17] Dennen, V. P., H. Choi, and K. Word. 2020. Social media, teenagers, and the school context: A scoping review of research in education and related fields. *Educational Technology Research and Development* 68: 1635–1658.

[CR18] Dymond, C. C., S. Tedder, D. L. Spittlehouse, B. Raymer, K. Hopkins, K. McCallion, and J. Sandland. 2014. Diversifying managed forests to increase resilience. *Canadian Journal of Forest Research* 44: 1196–1205.

[CR19] Falk, D. A., P. J. van Mantgem, J. E. Keeley, R. M. Gregg, C. H. Guiterman, A. J. Tepley, D. J. Young, and L. A. Marshall. 2022. Mechanisms of forest resilience. *Forest Ecology and Management* 512: 120129.

[CR20] Fedele, G., B. Locatelli, and H. Djoudi. 2017. Mechanisms mediating the contribution of ecosystem services to human well-being and resilience. *Ecosystem Services* 28: 43–54.

[CR21] Filatova, T., P. H. Verburg, D. C. Parker, and C. A. Stannard. 2013. Spatial agent-based models for socio-ecological systems: Challenges and prospects. *Environmental Modelling & Software* 45: 1–7.

[CR22] Folke, C. 2006. Resilience: The emergence of a perspective for social–ecological systems analyses. *Global Environmental Change* 16: 253–267.

[CR23] Folke, C., R. Biggs, A. V. Norström, B. Reyers, and J. Rockström. 2016. Social-ecological resilience and biosphere-based sustainability science. *Ecology and Society* 21: 16.

[CR24] Gillson, L., C. Whitlock, and G. Humphrey. 2019. Resilience and fire management in the Anthropocene. *Ecology and Society* 24: 14.

[CR25] Guz, J., and D. Kulakowski. 2020. Forests in the Anthropocene. *Annals of the American Association of Geographers* 111: 869–879.

[CR26] Hendrati, R. L. 2019. Environmental and social sustainability: The role of forest as the most influential ecosystem. In: *IOP conference series: Earth and environmental science*.

[CR27] Holling, C. S. 1973. Resilience and stability of ecological systems.

[CR28] Ibáñez, I., K. Acharya, E. Juno, C. Karounos, B. R. Lee, C. McCollum, S. Schaffer-Morrison, and J. Tourville. 2019. Forest resilience under global environmental change: Do we have the information we need? A systematic review. *PLoS ONE* 14: e0222207. 31513607 10.1371/journal.pone.0222207 PMC6742408

[CR29] Islam, M. A., D. J. Paull, A. L. Griffin, and S. Murshed. 2020. Assessing ecosystem resilience to a tropical cyclone based on ecosystem service supply proficiency using geospatial techniques and social responses in coastal Bangladesh. *International Journal of Disaster Risk Reduction* 49: 17.

[CR30] Italiano, S. S., J. J. Camarero, M. Colangelo, M. Borghetti, M. Castellaneta, M. Pizarro, and F. Ripullone. 2023. Assessing forest vulnerability to climate change combining remote sensing and tree-ring data: Issues, needs and avenues. *Forests* 14: 1138.

[CR31] Jia, G., E. Shevliakova, P. Artaxo, N. De Noblet-Ducoudré, R. Houghton, J. House, K. Kitajima, and C. Lennard. 2019. Land-climate interactions. In *Climate change and land: An IPCC special report on climate change, desertification, land degradation, sustainable land management, food security, and greenhouse gas fluxes in terrestrial ecosystems* (pp. 131–247). Cambridge University Press.

[CR32] Karamouz, M., E. Rasoulnia, M. Olyaei, and Z. Zahmatkesh. 2018. Prioritizing investments in improving flood resilience and reliability of wastewater treatment infrastructure. *Journal of Infrastructure Systems* 24: 04018021.

[CR33] Kelly, C., A. Ferrara, G. A. Wilson, F. Ripullone, A. Nolè, N. Harmer, and L. Salvati. 2015. Community resilience and land degradation in forest and shrubland socio-ecological systems: Evidence from Gorgoglione, Basilicata, Italy. *Land Use Policy* 46: 11–20.

[CR34] Kharrazi, A., B. D. Fath, and H. Katzmair. 2016. Advancing empirical approaches to the concept of resilience: A critical examination of panarchy, ecological information, and statistical evidence. *Sustainability* 8: 935.

[CR35] Landis, J. R., and G. G. Koch. 1977. The measurement of observer agreement for categorical data. *Biometrics* 33:159174. 843571

[CR36] le Polain de Waroux, Y. M. C. Carignan, O. del Giorgio, L. Diaz, E. Lucas, P. Jaureguiberry, M. L. Lipoma, and F. Mazzini. 2024. How do we study resilience? A systematic review. *People and Nature *6: 474–489.

[CR37] Ma, Y., Z. Kalantari, and G. Destouni. 2023. Infectious disease sensitivity to climate and other driver-pressure changes: Research effort and gaps for Lyme disease and cryptosporidiosis. *GeoHealth* 7: e2022GH000760. 10.1029/2022GH000760 PMC10251199 37303696

[CR38] Mayar, K., D. G. Carmichael, and X. Shen. 2022. Stability and resilience—a systematic approach. *Buildings* 12: 1242.

[CR39] Messier, C., J. Bauhus, R. Sousa-Silva, H. Auge, L. Baeten, N. Barsoum, H. Bruelheide, B. Caldwell, et al. 2022. For the sake of resilience and multifunctionality, let’s diversify planted forests! *Conservation Letters* 15: 8.

[CR40] Mina, M., C. Messier, M. J. Duveneck, M. J. Fortin, and N. Aquilué. 2022. Managing for the unexpected: Building resilient forest landscapes to cope with global change. *Global Change Biology* 28: 4323–4341. 35429213 10.1111/gcb.16197 PMC9541346

[CR41] Moreno-Fernández, D., M. A. Zavala, J. Madrigal-González, and F. Seijo. 2021. Resilience as a moving target: An evaluation of last century management strategies in a dry-edge maritime pine ecosystem. *Forests* 12: 1151.

[CR42] Myllyviita, T., T. Hujala, A. Kangas, K. Eyvindson, S. Sironen, P. Leskinen, and M. Kurttila. 2014. Mixing methods–assessment of potential benefits for natural resources planning. *Scandinavian Journal of Forest Research* 29: 20–29.

[CR43] Newton, A. C. 2011. Social-ecological resilience and biodiversity conservation in a 900-year-old protected area. *Ecology and Society* 16: 23.

[CR44] Nikinmaa, L., M. Lindner, E. Cantarello, A. S. Jump, R. Seidl, G. Winkel, and B. Muys. 2020. Reviewing the use of resilience concepts in forest sciences. *Current Forestry Reports* 6: 61–80. 35747899 10.1007/s40725-020-00110-x PMC7612878

[CR45] Nikinmaa, L., M. Lindner, E. Cantarello, B. Gardiner, J. B. Jacobsen, A. S. Jump, C. Parra, T. Plieninger, et al. 2023. A balancing act: Principles, criteria and indicator framework to operationalize social-ecological resilience of forests. *Journal of Environmental Management* 331: 14. 10.1016/j.jenvman.2022.117039 36701888

[CR46] Pérez, J., J. Díaz, J. Garcia-Martin, and B. Tabuenca. 2020. Systematic literature reviews in software engineering—enhancement of the study selection process using Cohen’s kappa statistic. *Journal of Systems and Software* 168: 110657.

[CR47] Randhir, T. O., and A. Erol. 2013. Emerging threats to forests: Resilience and strategies at system scale. *American Journal of Plant Sciences* 4: 739–748.

[CR48] Rani, N., and S. Sangwan. 2022. Advantages of ecosystem services to human being. *Current Journal of Applied Science and Technology* 41: 19–24.

[CR49] Reyer, C. P., N. Brouwers, A. Rammig, B. W. Brook, J. Epila, R. F. Grant, M. Holmgren, F. Langerwisch, et al. 2015. Forest resilience and tipping points at different spatio-temporal scales: Approaches and challenges. *Journal of Ecology* 103: 5–15.

[CR50] Riva, M. J., J. Baeza, S. Bautista, M. Christoforou, I. N. Daliakopoulos, D. Hadjimitsis, J. J. Keizer, H. Liniger, et al. 2018. How does land management contribute to the resilience of Mediterranean forests and rangelands? A participatory assessment. *Land Degradation & Development* 29: 3721–3735.

[CR51] Rokaya, P., S. Budhathoki, and K.-E. Lindenschmidt. 2018. Ice-jam flood research: A scoping review. *Natural Hazards* 94: 1439–1457.

[CR52] Sarkki, S., A. Ficko, F. E. Wielgolaski, E. M. Abraham, S. Bratanova-Doncheva, K. Grunewald, A. Hofgaard, F. K. Holtmeier, et al. 2017. Assessing the resilient provision of ecosystem services by social-ecological systems: Introduction and theory. *Climate Research* 73: 7–15.

[CR53] Schlüter, M., R. Biggs, M. L. Schoon, M. D. Robards, and J. M. Anderies, 2015. Reflections on building resilience—interactions among principles and implications for governance. *Principles for Building Resilience: Sustaining Ecosystem Services in Social-Ecological Systems*, 251–282.

[CR54] Seidl, R., T. A. Spies, D. L. Peterson, S. L. Stephens, and J. A. Hicke. 2016. Searching for resilience: Addressing the impacts of changing disturbance regimes on forest ecosystem services. *Journal of Applied Ecology* 53: 120–129. 26966320 10.1111/1365-2664.12511 PMC4780065

[CR55] Shumi, G., J. Loos, and J. Fischer. 2024. Applying social-ecological system resilience principles to the context of woody vegetation management in smallholder farming landscapes of the Global South [Review]. *Ecosystems and People* 20: 17.

[CR56] Tricco, A. C., E. Lillie, W. Zarin, K. K. O’Brien, H. Colquhoun, D. Levac, D. Moher, M. D. Peters, et al. 2018. PRISMA extension for scoping reviews (PRISMA-ScR): Checklist and explanation. *Annals of Internal Medicine* 169: 467–473. 30178033 10.7326/M18-0850

[CR57] Turner, B., T. Devisscher, N. Chabaneix, S. Woroniecki, C. Messier, and N. Seddon. 2022. The role of nature-based solutions in supporting social-ecological resilience for climate change adaptation. *Annual Review of Environment and Resources* 47: 123–148.

[CR58] Van Meerbeek, K., T. Jucker, and J. C. Svenning. 2021. Unifying the concepts of stability and resilience in ecology. *Journal of Ecology* 109: 3114–3132.

[CR59] Vigouroux, G., and G. Destouni. 2022. Gap identification in coastal eutrophication research–scoping review for the Baltic system case. *Science of the Total Environment* 839: 156240. 35644392 10.1016/j.scitotenv.2022.156240

[CR60] Wu, T., and Y. S. Kim. 2013. Pricing ecosystem resilience in frequent-fire ponderosa pine forests. *Forest Policy and Economics* 27: 8–12.

[CR61] Wyatt, K. H., K. K. Arkema, S. Wells-Moultrie, J. M. Silver, B. Lashley, A. Thomas, J. J. Kuiper, A. D. Guerry, et al. 2021. Integrated and innovative scenario approaches for sustainable development planning in the Bahamas. *Ecology & Society* 26: 18.

[CR62] Zarei, M., and G. Destouni. 2024. Research gaps and priorities for terrestrial water and earth system connections from catchment to global scale. *Earth’s Future* 12: e2023EF003792.

