# Supplementary material for: Research efforts and gaps in the assessment of forest system resilience: A scoping review
Source: Ambio. 2025 Sep 10;55(3):479–96. doi: 10.1007/s13280-025-02243-4 (PMC12868454; doi:10.1007/s13280-025-02243-4)
Supplement: Supplementary file 1 — Supplementary file1 (PDF 858 kb) [file 13280_2025_2243_MOESM1_ESM.pdf]

Supplementary Information

Research Efforts and Gaps in the Assessment of Forest System Resilience: A Scoping Review

Sara Anamaghi, Massoud Behboudian, Mohammad Javad Emami-Skardi, Elisie Kåresdotter, Carla Sofia Santos Ferreira, Georgia Destouni, Lan Wang-Erlandsson, Anna Tengberg, Fabian Stenzel, Ingo Fetzer, Najmeh Mahjouri, Reza Kerachian, Zahra Kalantari

Note S1

Resilience has been defined and interpreted in different ways based on the scope in which it is being implemented. Generally, resilience has been used to describe a system's characteristics, including its ability to withstand disturbances (i.e. resistance), return to its pre-disturbance state (i.e. recovery), and address changes (i.e. adaptive capacity) (Behboudian et al., 2023; Holling, 1973; Pimm, 1984; Yi and Jackson, 2021). According to Nikinmaa et al. (2020), the three main concepts of resilience in forest resilience studies are engineering resilience, ecological resilience, and social-ecological resilience, with scholars, depending on the nature of their research, focusing on one of them.

Engineering resilience mainly focuses on the system's recovery time after facing shocks and disturbances (Pimm, 1984). This concept assumes that the impacts of these disturbances on the system are temporary and will eventually return to its pre-disturbance state (i.e. equilibrium state or basin of attraction) (Hlásny et al., 2021). By incorporating this concept, Heinimann (2010) sought to clarify and develop adaptive ecosystem management concepts by integrating system theory concepts, control theory, and risk management to increase resilience.

Ecological resilience encompasses a more comprehensive definition than mere recovery time and emphasises on the system's ability to absorb changes and persist (Bryant et al., 2019). It is built on the premise that the disturbances can transition the system to a new stable state, reflecting the presence of multiple equilibria (Holling, 1973). Since the introduction of this concept, several studies have advocated examining the ecological resilience of forests (Chambers et al., 2023; Ekblom et al., 2012; Garate-Quispe et al., 2024; Li et al., 2022; Liao et al., 2023; Ma et al., 2023b; Pomara and Lee, 2021; Rocha, 2022; Rodríguez-Rodríguez et al., 2023; Yang et al., 2024). To investigate ecological resilience, Bryant et al. (2019) proposed and employed an index-based

framework in ponderosa pine and dry mixed conifer forests to fire, insects, and droughts. They also examined the impacts of management strategies on forest resilience. Mina et al. (2022) investigated the ecological resilience of forests in south-east Canada under different climate change and insect outbreak scenarios using the LANDIS-II landscape model. They highlighted the importance of functional and structural diversity and functional connectivity in enhancing forest resilience towards these disturbances. Various indicators have been developed to quantify forest resilience. Ma et al. (2023a) integrated ecological resilience and landscape pattern indicators of ecological sources to calculate the total ecological resilience value and examined its characteristics in the Yangtze River Delta region in China. Yang et al. (2024) introduced a new remote sensing method based on net ecosystem productivity (NEP) to quantify the long-term effects of ecological engineering projects on resilience in evergreen forest ecosystems in subtropical regions of China.

Macro Code:

```
Sub Checkwords()
    Dim textToSearch As String
    Dim wordToFind As String
    Dim found As Boolean
    Dim x1

x1 = Cells(Rows.Count, "D").End(xlUp).Row + 1
'MsgBox x1

For i = 1 To x1
```

```
textToSearch = Sheet1.Cells(i, "A")[1]

wordToFind = "The word you're looking for"[2]

found1 = InStr(1, textToSearch, wordToFind, vbTextCompare) > 0

If found1 Or found2 Or found3 Then

    Sheet1.Cells(i, "W") [3]= 1
    Sheet1.Cells(i, "W").Interior.Colour = RGB(0, 255, 0)
Else
End If
Next i
End Sub
```

[1] The letters in the following tree lines (e.g. A) denote the column in which the specific word is searched.
[2] The word being searched should be written here.
[3] The letter here (i.e. "w") refers to the column in which the outputs are shown.

S1. Resilience principles, criteria and sub-criteria

Principles and their definition	Criterion	Explanation	Sub-criterion	Explanation	Examples of the usage in literature	
					References	Explanation
Diversity and redundancy: Diversity refers to the multiplicity of different elements and features of the system that exhibit diverse responses to disturbances. Redundancy refers to alternative elements and features with the same function within the system that can replace each other when the system encounters disruptions or perturbations	Diversity	Diversity refers to a system's variety of elements or features that can respond to disturbances; in other words, elements that show different responses to disturbances provide diversity. According to Mori et al. (2017) sufficient level of diversity helps ecosystems withstand environmental changes and continue functioning, playing a key role in maintaining their resilience.	Resilient species	The existence of resilient species (i.e. species that have developed adaptable mechanisms to withstand shocks or extreme events) can enhance the system's overall resilience.	(Asbjornsen et al., 2014)	This paper discusses the impact of resilient, or perennial, species on resilience in ecosystems and that these species contribute to enhancing resilience.
					(Fang and Zhang, 2019)	This paper investigates the response of resilient species, here Juniper trees, to droughts. They use tree ring width as an indicator to assess their resilience.
					(Jackson et al., 2007)	This paper highlights that resilient species can handle certain stresses better, meaning that the overall ecosystem can perform well under adverse conditions, like drought or pest outbreak.
					(Ilunga et al., 2015)	The paper emphasizes the importance of choosing resilient species with traits such as strong spreading ability, durable root systems, and adaptable dispersal strategies for effective revegetation in metal-rich environments. This strategy helps restore ecosystem functions and promote biodiversity in degraded areas.
					(Klein et al., 2018)	This paper asserts that plants with specific traits adapted to withstand disturbances can bring about resilience in the face of disturbances.
					(Devisscher et al., 2021)	The paper highlights that building resilience involves addressing uncertainty and adopting alternative practices, such as selecting and managing resilient species, to adapt to future climate-related risks.

S1. Resilience principles, criteria and sub-criteria

Principles and their definition	Criterion	Explanation	Sub-criterion	Explanation	Examples of the usage in literature	
					References	Explanation
Diversity and redundancy	Diversity		Species composition	This criterion refers to the number of species and the relative abundance of organisms in each species.	(Hlásny et al., 2021)	This paper reveals that correlation between species composition, here referred as alpha diversity, with recovery rate after the disturbance.
					(Jansson and Polasky, 2010)	The paper argues that maintaining resilience in ecosystems requires a focus on both species composition and the functional traits, as well as the diversity within functional groups to ensure the continued generation of ecosystem services in the face of change.
					(Rosenfield et al., 2023)	The authors have pointed out the importance of species composition in ecological integrity and resilience of secondary forests
					(Seidl et al., 2016)	This paper underscores the fact that species diversity increases the response diversity to changing environmental conditions.
					(Jackson et al., 2007)	According to this paper, it is important to assess species richness and community composition to maximize both economic benefits and environmental quality which are conducive to resilience.
					(Mina et al., 2022)	In this paper, functional diversity as the component of Shannon diversity index relative above ground biomass of species to assess species composition.
			Species richness	The higher number of different species in an area ensures the existence of different responses to disturbance and can lead to higher levels of resilience.	(Turner et al., 2022)	This research emphasizes that sufficient level of species richness guarantees the presence of several species capable of fulfilling analogous ecological functions, hence augmenting the system's resilience and recovery from disruptions.

S1. Resilience principles, criteria and sub-criteria

Principles and their definition	Criterion	Explanation	Sub-criterion	Explanation	Examples of the usage in literature	
					References	Explanation
Diversity and redundancy	Diversity		Species richness		(Cantarello et al., 2017)	Used species richness criterion, calculated based on the data collected from field study and the outputs of the LANDIS-II model, to evaluate resilience.
					(Jackson et al., 2007)	This paper suggests that higher species richness can enhance ecosystem's resilience and its ability to recover from disturbances.
					(Arenas-Wong et al., 2023)	The authors highlight that biodiversity, particularly the variety of woody species, influences the forest's ability to withstand and recover from extreme climatic events.
					(Rosenfield et al., 2023)	This paper introduces species richness as one of the important indicators of ecological integrity and resilience.
			Soil type	Different soil types with different storage capacities of water and nutrients can provide growth conditions for a plethora of flora and habitats for fauna. According to de la Mora et al. (2021) soil characteristic is crucial for maintaining	(Suarez-Pardo et al., 2022)	The authors assert that the soil cover and type contribute to the diversity and redundancy of landscape species; hence can affect resilience.
					(Rosenfield et al., 2023)	This paper reports that soil physical and/or chemical parameters were used as resilience indicator in at least 11 studies.
					(de la Mora et al., 2021)	This paper mentions the trait and characteristics of the fertile soils in Mexican temperate forests
					(Li et al., 2022)	In this study, the authors assert that soil type is important for the recovery and vegetation and stability of the ecosystem and investigates which type of soils provided rapid recovery in a case study.

S1. Resilience principles, criteria and sub-criteria

Principles and their definition	Criterion	Explanation	Sub-criterion	Explanation	Examples of the usage in literature	
					References	Explanation
Diversity and redundancy	Redundancy	-	Soil type	-	(Wonkka et al., 2016)	Through field experiments, the authors determined the impact of soil texture on the ability of the ecosystem to absorb disturbances or recover from them.
		Redundancy refers to the existence of components with the same function within the system that can replace each other in times of disturbance. Jackson et al. (2007), also noted that species that exhibit functionality redundancy, can provide diverse responses to environmental fluctuations, so contributing to the stabilization of aggregate ecosystem functions over time.	Availability of different water resources	If the habitants of the forest have access to abundant water resources, their wellbeing can be maintained.	(Nikinmaa et al., 2020)	This review indicates that in 10% of the studies relevant to socio-ecological forest resilience, water and sanitation have been considered as resilience indicators.
					(Turner et al., 2022)	This study underscores that having access to multiple water sources, such as rivers, lakes, groundwater, and rainwater harvesting systems, provides a buffer against the impacts of climate change and variability, such as droughts and floods.
			Different occupational options	The existence of different occupational options to meet the economic needs of the people can reduce their vulnerability in the times of the disturbance and enhance redundancy. For example, investment in diverse ES-based activities (e.g. fishing, ecotourism) can enhance the resilience of associated livelihoods.	(Arenas-Wong et al., 2023)	The study suggests that a diversified economy may reduce the vulnerability of local people to extreme events.
					(Nikinmaa et al., 2020)	This review represents income and employment as indicators of social-ecological resilience.
					(Cotroneo et al., 2021)	The paper mentions that economic diversification can reduce the vulnerability of communities and bolster resilience in the face of forest degradation.
					(Turner et al., 2022)	This paper recognizes livelihood diversity as on ethe resilience mechanisms.
					(Suarez-Pardo et al., 2022)	This paper introduces diversified economic activities of households as one of the key variables of resilience.

S1. Resilience principles, criteria and sub-criteria

Principles and their definition	Criterion	Explanation	Sub-criterion	Explanation	Examples of the usage in literature	
					References	Explanation
Diversity and redundancy	Redundancy	-	Different occupational options	-	(Ferrara et al., 2016)	This study implies that economic activities impact resilience and proposes several criteria (e.g., density of enterprise local unit of production n/km^2) to investigate the economic capital of the society.
					(Newton, 2011)	This study notes that the shift from subsistence agriculture to other forms of economic activities has influenced the resilience of the New Forest in UK.
Managing connectivity: connectivity reflects the manner and degree to which resources, species, or social actors disperse, migrate, or interact across ecological and social landscapes.	Connectivity between habitats	Fragmentation of forests resulting from the construction of roads, residential or urban areas, and other factors can hinder the free flow of species and resources between different forest areas, thereby negatively affecting the forest's resilience and ecosystem services (ESs). Road construction within a forest, for instance, may impede the dispersal of seeds or be an obstacle for fauna, thereby restricting their access to food. Therefore, the presence of corridors[4] connecting habitats can facilitate the distribution of resources. According to Turner et al. (2022), this kind of connectivity facilitates the recovery from unexpected disturbances by providing the means for resource flow between different forest patches.	-	-	(Jansson and Polasky, 2010)	The study highlights the importance of spatial distribution and habitat connectivity for pollinator survival, suggesting that mapping declines in pollination potential can help guide management strategies to maintain resilience.
					(Rosenfield et al., 2023)	The paper mentions landscape heterogeneity and connectivity as resilience indicators.
					(Suarez-Pardo et al., 2022)	The authors assert that forest connectivity is one of the key variables in assessing resilience.
					(Sui et al., 2023)	In this paper, different indices such as patch density and edge density are used to evaluate connectivity. The results indicate that high connectivity can facilitate quicker recovery.
					(Soubry et al., 2021)	This study recognizes connectivity between adjacent similar patch types as one of the important attributes of forest health and resilience.

[4] A wildlife corridor, habitat corridor or green corridor is an area of habitat connecting wildlife populations separated by human activities or structures.

S1. Resilience principles, criteria and sub-criteria

Principles and their definition	Criterion	Explanation	Sub-criterion	Explanation	Examples of the usage in literature	
					References	Explanation
Managing connectivity	Connectivity between habitats	-	-	-	(Enoki et al., 2014)	The authors highlight the importance of connectivity between terrestrial ecosystems in forest resilience.
	Connectivity between human communities	Communities within forests should have access to each other. Existence of communication channels between human communities that are separated by forests can facilitate the flow of resources and information. This can also enhance the resilience of ESs and overall resilience of forests.	-	-	(Raj and Sharma, 2023)	The paper introduces road networks as an indicator of ecosystem resilience.
					(Kleinschroth and Healey, 2017)	In this paper, it is noted that roads can provide improved access to health care and education and therefore have an impact on resilience.
					(Enoki et al., 2014)	They underscore the importance of connectivity between adjacent habitats in forest systems resilience.
					(Beier et al., 2008)	The authors use road distance as a criterion to assess vulnerability and resilience in managed ecosystems.
					(Wang et al., 2021)	This study uses road data to investigate the resilience of ecological networks.
	Interaction between species	Species interactions can harm both species involved (competition), benefit both involved (mutualism), or benefit one and harm the other (predation, parasitism, herbivory). For example, the preference of predators for a specific species can result in extinction of that species and a decrease in biodiversity or disturbance in ESs. Hence, an adequate level of interaction between species should be maintained.	-	-	(Soubry et al., 2021)	The authors assert that according to literature review, biotic interaction is one of the most important attributes when examining forest and grassland health and resilience.

S1. Resilience principles, criteria and sub-criteria

Principles and their definition	Criterion	Explanation	Sub-criterion	Explanation	Examples of the usage in literature	
					References	Explanation
Managing connectivity	Interaction between species	-	-	-	(Haberstroh and Werner, 2022)	The paper highlights that Positive interactions like facilitation and complementarity can enhance resilience under mild to moderate droughts, while negative interactions like competition can reduce resilience under severe to extreme droughts. These interactions can be understood through tree sap flow, transpiration, stable water isotopes, and leaf water potential.
					(Samani et al., 2023)	The study explores how community-centered ecotourism planning, involving local participation, can improve the management of the Bossou Forest Reserve and boost social-ecological resilience. For this means one of the indicators they use is the interactions between human communities and chimpanzees.
					(Chazdon and Guariguata, 2016)	The paper emphasizes that mutualistic species interactions boost biodiversity and ecological diversity, strengthening forest resilience by supporting adaptation, maintaining ecosystem functions, and aiding recovery from disturbances.
					(Cottrell et al., 2020)	They highlight how bark beetles, such as the mountain pine beetles, influence forest structure and function by regulating aspects like primary production, nutrient cycling, and ecological succession.

S1. Resilience principles, criteria and sub-criteria

Principles and their definition	Criterion	Explanation	Sub-criterion	Explanation	Examples of the usage in literature	
					References	Explanation
Managing connectivity	Interaction between species	-	-	-	(Martínez and García, 2017)	The study emphasizes the vital role of frugivorous birds and scrub vegetation in seed dispersal, seedling protection, and tree establishment, underscoring their combined importance for forest resilience and natural regeneration.
	Connectivity between forests and water bodies[5]	-	-	-	(Soubry et al., 2021)	The paper underscores that the degree of connectivity with adjacent systems, including aquatic systems, can influence resilience.
					(Enoki et al., 2014)	The paper states that the connectivity between forest and river or other water bodies plays an important role in constructing the community and providing a means for material flow within the system.
					(Dutra et al., 2022)	Forest fragmentation within the context of the basin can significantly reduce water availability, weaken environmental resilience.

[5] If the forest is in the vicinity of a water body, this criterion may be applicable, otherwise it can be neglected.

S1. Resilience principles, criteria and sub-criteria

Principles and their definition	Criterion	Explanation	Sub-criterion	Explanation	Examples of the usage in literature	
					References	Explanation
Slow variables and feedback: slow variables determine the underlying structure of the system evolving gradually over time. However, if these variables exceed a specific threshold, they can cause a significant shift in the system's regime. Feedback occurs when a change in a particular variable or process either amplifies or dampens subsequent changes of a similar nature in the system. Hence, these variables and feedback must be monitored closely.	Ecosystem-based criteria	By managing and monitoring forests for multiple values, including ecosystem services such as water protection, habitat quality, and recreation, the resilience of forests to disturbances can be enhanced (Devisscher et al., 2021).	Water quality or water retention	Clean water plays an important role in providing ESs such as drinking water and recreation. Moreover, pollution can harm forests and communities adjacent to forests.	(Kelly et al., 2015)	The paper highlights that water retention is one of the most important ecosystem services and is vital for forest resilience as it helps maintain soil stability, regulate hydrological cycles, reduce run-off, and support vegetation growth, all of which contribute to the overall health and sustainability of forest ecosystems.
					(Ma et al., 2023a)	The study considers annual water yield which is calculated based on average annual precipitation and evapotranspiration to assess the resilience of different landscapes, including forest.
					(Seddon et al., 2016)	In this paper, the ratio of actual to potential evapotranspiration is used as an indicator of water availability, which is believed to influence the resilience of terrestrial ecosystems.
					(Suarez-Pardo et al., 2022)	This study mentions available water retention capacity is one of the key variables of resilience and can be assessed as water storage capacity of the soil.
					(Penaluna et al., 2017)	The paper highlights how forests support water retention, influencing water quality, biodiversity, and resilience.
			Soil erosion rate	Soil erosion can decrease the overall survival opportunities of the forest system.	(Ma et al., 2023a)	They used the outputs of the RULSE model to estimate this criterion, for assessing the resilience of different landscape types.
					(Soubry et al., 2021)	According to this review paper, soil erosion is one of the important factors in assessing ecosystem health and resilience.

S1. Resilience principles, criteria and sub-criteria

Principles and their definition	Criterion	Explanation	Sub-criterion	Explanation	Examples of the usage in literature	
					References	Explanation
Slow variables and feedback	Ecosystem-based criteria	-	Soil erosion rate	-	(Rosenfield et al., 2023)	Highlights this criterion as an important and frequently assessed one in forest resilience studies.
					(Kelly et al., 2015)	The authors assert that soil erosion is a critical factor affecting forest resilience. They highlight that addressing soil erosion through sustainable land management practices and reforestation efforts is essential for maintaining the health and resilience of forest ecosystems.
					(Raj and Sharma, 2023; Seidl et al., 2016)	These papers, also introduce this criterion influential in assessing resilience.
			Habitat air quality	The emergence of dust-prone zones as a result of deforestation can reduce air quality. Particulate matter can disrupt photosynthesis and have adverse impacts on vegetation cover. Furthermore, it can have adverse impacts on the health and well-being of humans.	(Drillet et al., 2020)	The paper mentions the importance of air quality as a regulating service, mainly provided by trees which can directly influence the people's quality of life in the adjacent area.
					(Sui et al., 2023)	They used the habitat quality module in the InVEST model to evaluate this criterion. They mention habitat quality is an essential criterion for measuring the well-functioning of the environment.
					(Rosenfield et al., 2023)	The paper notes that air quality, as an ecosystem service, can be assessed using indicators like "Tree size and biomass" and "Canopy cover and structure". These indicators help evaluate secondary forest ecological integrity and resilience.

S1. Resilience principles, criteria and sub-criteria

Principles and their definition	Criterion	Explanation	Sub-criterion	Explanation	Examples of the usage in literature	
					References	Explanation
Slow variables and feedback	Ecosystem-based criteria	-	Pest control	Monitoring the pest population is of great importance.	(Soubry et al., 2021)	The paper identifies insect infestation as the second biggest ecological stressor for forests after climate change, highlighting the need for pest management and standardized monitoring tools like remote sensing and GIS to maintain forest health and resilience.
					(Rosenfield et al., 2023)	According to this study, pest control is an important aspect of ecosystem services that supports the resilience of tropical secondary forests by helping to maintain ecological balance and health.
					(Takahashi and Liang, 2016)	The paper emphasises the importance of traditional practices to help maintain and enhance resilience against environmental changes and pest outbreaks.
					(Tscharntke et al., 2011)	The paper highlights that shade trees enhance resilience by supporting natural pest control, reducing pesticide use, and mitigating pest outbreaks.
			Carbon sequestration	Forests are one of the major sources of the carbon sequestration. The demise of vegetative cover can lead to the increase of the atmospheric carbon and add to the detrimental impacts of the global warming. Moreover, the relevance of carbon storage and forest resilience lies in their combined role in maintaining ecosystem health, supporting climate change mitigation efforts, and informing adaptive forest management practices.	(Ma et al., 2023a)	In this study, carbon sequestration rate and the average market transaction prices of carbon sequestration are used to assess the resilience of forest landscape.

S1. Resilience principles, criteria and sub-criteria

Principles and their definition	Criterion	Explanation	Sub-criterion	Explanation	Examples of the usage in literature	
					References	Explanation
Slow variables and feedback	Ecosystem-based criteria	-	Carbon sequestration	-	(Mina et al., 2022)	The authors use Net Primary Productivity (NPP) as direct indicators of carbon accumulation.
					(Rosenfield et al., 2023)	Introduces carbon sequestration and productivity as an indicator of resilience.
					(Gauthier et al., 2015)	This paper asserts that the resilience of boreal forests is directly linked to their capacity to capture and store carbon, which is essential for climate regulation and mitigating the impacts of climate change.
					(Yang et al., 2024)	In this paper carbon stock resilience has been introduces as a criterion to investigate the subtropical forest resilience.
			Nutrient cycles (carbon cycle, Nitrogen cycle)	Soil quality is an important factor in social-ecological frameworks as it can impact the productivity of ecosystems and the provision of ESs.	(Hlásny et al., 2021)	The soil nitrogen is considered as the part of the environmental variables in model simulation to simulate the resilience of aboveground biomass.
					(de la Mora et al., 2021)	Considers soil quality and mineral storage as one of the criteria used for assessing sustainability and resilience.
					(Soubry et al., 2021)	The study introduces soil chemistry and structure as one of the factors that influence forest resilience.
					(Ferrara et al., 2016)	The authors consider soil quality as one of the forest resilience influential variables.
					(Meacham et al., 2016)	The nitrogen and phosphorous cycles are measured as the average fraction of 1-(net nutrient load/Gross nutrient load) in this study.
			Tourism and recreation	Given the circumstances of the site, the tourism industry can ameliorate the status of the area by providing extra attention to maintenance and wellbeing of the area.	(Cantarello et al., 2017)	The paper highlights the importance of different ecosystem services in the context of forest resilience and assess its value through a survey.

S1. Resilience principles, criteria and sub-criteria

Principles and their definition	Criterion	Explanation	Sub-criterion	Explanation	Examples of the usage in literature	
					References	Explanation
Slow variables and feedback	Ecosystem-based criteria	-	Tourism and recreation	-	(Meacham et al., 2016)	Moose haunting, outdoor recreation, summer cottages, horseback riding, cross country skiing are used as indicators of tourism.
					(Rosenfield et al., 2023; Seidl et al., 2016)	These papers highlight the importance of tourism and recreation in forests.
			Educational value	This ES refers to the features of the ecosystem that could be used to earn knowledge about natural environment	(Johnson et al., 2021)	The document emphasizes the role of forest-based educational activities, such as culture classes and camps, in fostering intergenerational knowledge transfer, cultural identity, and environmental stewardship, which collectively enhance community and forest resilience.
					(Souza-Alonso et al., 2022)	The paper brings example on how educational activities tied to forests enhance resilience by preserving cultural knowledge, fostering intergenerational learning, promoting environmental stewardship, and strengthening community cohesion.
					(Nevzati et al., 2023)	The paper highlights the educational value, here referred to as "cognitive value" of landscapes, emphasizing their role in promoting learning, curiosity, and intellectual engagement, with Coastal Forest, Mixed Forest and Wetland, and Mixed Forest noted for their strong educational potential.
			Aesthetic inspiration for culture and art	Natural sceneries are a great source of inspiration. Thus, degradation of these sceneries can impact art works.	(Drillet et al., 2020; Rosenfield et al., 2023)	These papers, introduce this ecosystem service as a significant cultural ecosystem service in the forests.
					(Johnson et al., 2021)	Aesthetic values contribute to resilience by fostering cultural identity, community cohesion, and spiritual well-being through artistic creations.

S1. Resilience principles, criteria and sub-criteria

Principles and their definition	Criterion	Explanation	Sub-criterion	Explanation	Examples of the usage in literature	
					References	Explanation
Slow variables and feedback	Ecosystem-based criteria	-	Health and well-being	Provision of forest-based ESs can promote human health and wellbeing.	(Pomara and Lee, 2021)	The paper highlights that forests support a wide range of ecosystem services that are integral to human health and well-being. Also, it underscores that forests offer recreational opportunities that promote physical activity, mental health, and overall well-being.
					(Nikinmaa et al., 2020)	This paper mentions that individual health and well-being is one of the frequently used criteria to evaluate social-ecological resilience of forest.
					(Rosenfield et al., 2023; Seidl et al., 2016)	These studies also underscore the role of forests in promoting mental health and well-being.
			Cultural practices	Existence of indigenous cultures that provide forest protection measures can enhance the resilience of forests in the face of disturbance.	(Valipour et al., 2014)	Cultural practices enhance forest resilience by promoting sustainable resource use, conserving biodiversity, fostering community involvement, adapting to environmental changes, and mitigating conflicts.
					(Ruiz-Mallén and Corbera, 2013)	This paper highlights the role of traditional ecological knowledge and implementing cultural practices in enhancing forest resilience. It mentions that ecological knowledge enhances resilience by enabling informed decision-making, adaptive management, cultural integration, institutional flexibility, social cohesion, effective leadership, and cross-scale collaboration, ensuring sustainable practices and ecosystem stability.

S1. Resilience principles, criteria and sub-criteria

Principles and their definition	Criterion	Explanation	Sub-criterion	Explanation	Examples of the usage in literature	
					References	Explanation
Slow variables and feedback	Ecosystem-based criteria	-	Cultural practices	-	(Kelly et al., 2015)	According to this paper, cultural practices are crucial for forest resilience as they influence how forests are managed and valued by the community. Maintaining these practices and integrating them into modern forest management can enhance the resilience of forest ecosystems.
			Spiritual values	The importance of belief in life and feeling spiritual in forests can provide protective measures for forest.	(Cottrell et al., 2020)	They highlight that incorporating spiritual, aesthetic, and recreational forest values into resource management enhances social-ecological resilience by aligning strategies with community needs, fostering support, and improving adaptation to disturbances.
					(Turner et al., 2022)	This study introduces the “attachment to place” criterion as one of the resilience mechanisms.
Complex adaptive systems (CAS) thinking: this principle posits that resilience enhancement is contingent upon an integrative and comprehensive management approach that considers various spatial and temporal scales and associated uncertainties. Such a management process focuses on the capacity of the system to adapt in the presence of uncertainty, disturbances and shocks, rather than on eliminating these.	Robustness	Robustness refers to the intrinsic characteristics of the system that enables it to withstand disturbances.	Longevity	This sub-criterion refers to the average age of the forest. Older forests generally have gone through several disturbances and as a result are more resilient in the face of climate change.	(Rosenfield et al., 2023)	Overall, forest age plays a crucial role in determining the resilience of secondary forests, with older forests generally exhibiting higher resilience due to increased structural complexity, biodiversity, and ecological functions.

S1. Resilience principles, criteria and sub-criteria

Principles and their definition	Criterion	Explanation	Sub-criterion	Explanation	Examples of the usage in literature	
					References	Explanation
Complex adaptive systems (CAS) thinking	Robustness		Longevity		(Reid et al., 2017)	The longevity of restored ecosystems, including forests, is important for their capacity to support biodiversity and provide ecosystem services, which are key components of resilience.
			Seed dispersal distance	Trees capable of dispersing their seeds over greater distances have better survival chances in places with more desirable conditions.	(Chazdon and Guariguata, 2016)	The paper highlights the crucial role of seed dispersal by fauna in forest regeneration, emphasizing how modular and nested seed dispersal networks contribute to stability and resilience of ecosystems.
					(Martínez and García, 2017)	The study shows that seed dispersal by frugivorous birds is a key ecological process that supports forest regeneration, enhances genetic diversity, and contributes to the resilience of forest ecosystems through a mixture of field surveys and GIS mapping.
			Density of seedlings	Seedlings are more vulnerable to disturbances and reduce the resilience level of the forest	(Nikinmaa et al., 2020)	This study introduces density of seedlings as one of the indicators for assessing engineering resilience.
					(Stevens-Rumann et al., 2018)	According to this study, seedling density is crucial for forest resilience, reflecting its ability to regenerate and maintain stability, with declines signaling reduced recovery and potential shifts to non-forested states under climate stress.
			Vegetation cover	Degradation of vegetation covers can result in disruption in provisioning ESs (e.g. food), regulating ESs (e.g. flood), and cultural ESs (e.g. ecotourism). The denser the vegetation cover, the more robust the system.	(Li et al., 2022)	This paper states that the presence of a robust vegetation cover before the fire can provide a source of seeds and organic matter that support post-fire recovery.
					(Frazier et al., 2013)	Introduces an index for assessing ecosystem resilience based on NDVI (Normalized Difference Vegetation Index).
					(Ferrara et al., 2016)	This study considers vegetation quality index

S1. Resilience principles, criteria and sub-criteria

Principles and their definition	Criterion	Explanation	Sub-criterion	Explanation	Examples of the usage in literature	
					References	Explanation
Complex adaptive systems (CAS) thinking	Robustness	-	Vegetation cover	-	(Beier et al., 2008)	This study highlights that the density and structure of vegetation can influence resilience, mentioning that intensive logging and even-aged forest management practices that simplify vegetation structure can reduce resilience, making ecosystems more vulnerable to disturbances and less capable of sustaining ecosystem services.
					(Chazdon and Guariguata, 2016)	The paper underscores that diverse and well-developed vegetation cover is crucial for building resilient forest ecosystems, as it supports a variety of ecosystem functions and enhances the forest's ability to recover from disturbances.
					(Seddon et al., 2016)	The vegetation cover based on the EVI index (Enhanced Vegetation Index) is introduced as one of the factors affecting terrestrial ecosystem resilience.
			The geographic location and climatic properties of the forest	Forests located in plains are more resilient than mountainous forests, and forests with humid climate are more resilient than forests in arid regions.	(Hlásny et al., 2021)	This study investigates the impacts of changes in the climatic properties of the regions in the form of climate scenarios on the resilience.
					(de la Mora et al., 2021)	Mentions that the location of the forest is an important factor in implementing forest management practices that are conducive to more sustainability and resilience.
					(Freudenberger et al., 2012)	This paper highlights that geographic location, and climatic traits play crucial roles in determining the resilience of ecosystems by influencing their complexity, biodiversity, and ability to adapt to environmental changes.

S1. Resilience principles, criteria and sub-criteria

Principles and their definition	Criterion	Explanation	Sub-criterion	Explanation	Examples of the usage in literature	
					References	Explanation
Complex adaptive system (CAS) thinking	Robustness		Average duration of reproductive years	Longer reproductive years can ensure repopulation in the case of disturbance and enhance system's robustness.	-	-
			Forest succession stage	Forests can transition into savannahs due to changes in slow variables. Changes in precipitation, temperature, soil fertility and fire regime can gradually cause a nonlinear transition into an alternative stable state – in this case a savannah. Anthropogenic factors such as land-use change, habitat fragmentation, over-extraction of resources and the introduction of invasive species can increase the likelihood and speed of such transitions. Forests that are subjected to such changes for prolonged period (i.e. are at their tipping point) are less robust.	-	-
			Soil moisture	Forests with a higher soil moisture content are more resilient.	(Hlásny et al., 2021)	In this study, soil water availability, extracted from the iLand model is considered to investigate forest resilience.
					(Ferrara et al., 2016)	This paper has recognized soil moisture as one of the variables that can impact resilience.
					(Bryant et al., 2019)	The authors use a topographic moisture index as an indicator to assess resilience against drought. They set up specific thresholds to determine whether the forest is resilient or not.
					(Chambers et al., 2023)	In this study, annual mean length of dry soil interval as an indicator of drought resilience.
					(Danneyrolles et al., 2020)	The authors assert that site drainage, which is related to soil moisture, had an important effect on the probability of changes in forest cover.

S1. Resilience principles, criteria and sub-criteria

Principles and their definition	Criterion	Explanation	Sub-criterion	Explanation	Examples of the usage in literature	
					References	Explanation
Complex adaptive system (CAS) thinking	Robustness		Precipitation	Precipitation time series have a direct influence on system robustness. Precipitation is among the various factors that influences forest resilience (Hlásny et al., 2021). According to Ma et al. (2023a) physical conditions such as topography and precipitation along side biodiversity and human social impacts are core components of evaluating ecological resilience.	(Ma et al., 2023a)	The document indicates that precipitation (average annual precipitation (mm)) is a significant factor affecting ecological resilience
					(Chambers et al., 2023)	This study uses summer and driest month precipitation and annual precipitation as indicators of drought resilience.
					(Alfaro-Sánchez et al., 2019)	In this study, monthly mean precipitation was considered as one of the influential factors affecting recently established forests.
					(Rastetter et al., 2021)	The authors consider annual precipitation variability as an indicator for evaluating resilience. According to this paper, Overall, the variability, distribution, and intensity of precipitation events play a significant role in determining the resilience of ecosystems by influencing water availability, growth patterns, and the ability of species to withstand and recover from disturbances.
					The following studies use the mention criterion to assess precipitation in resilience studies:	
					(Hlásny et al., 2021)	Altered precipitation regimes
					(Frei et al., 2020)	The mean precipitation during the growing season
					(Huang et al., 2021)	Total annual precipitation
					(Wu et al., 2023)	The difference in precipitation between the drought year and the mean of three years before/after
					(Che et al., 2023)	The comparisons between the average monthly temperature and precipitation in extreme drought years
					(Liao et al., 2023)	Annual precipitation change rate

S1. Resilience principles, criteria and sub-criteria

Principles and their definition	Criterion	Explanation	Sub-criterion	Explanation	Examples of the usage in literature	
					References	Explanation
Complex adaptive system (CAS) thinking	Robustness		Basal area increment	This sub-criterion refers to the total cross-sectional area of tree stems at a 1.3 m height from the ground, used to indicate forest productivity and timber volume. Very dense or sparse vegetation cover can adversely impact resilience.	(Arenas-Wong et al., 2023)	The study highlights that old-growth forests have a higher basal area compared to secondary forests, which contributes to their greater resilience to extreme climatic events.
					(Wu and Kim, 2013)	The study uses basal area to assess forest resilience to fire, finding an inverse relationship between the two. Higher basal area increases competition and makes forests more vulnerable to fire, reducing resilience.
					(Bryant et al., 2019)	They used this criterion as one of the indicators to evaluate resilience.
					(Alfaro-Sánchez et al., 2019)	They found that recently established forests, with higher basal area growth and climate sensitivity, demonstrate similar or even greater recovery, resistance, and resilience to drought. Enhanced growth during wet periods helps offset the impacts of drought.
					(Rastetter et al., 2021)	This paper considers basal area increment (BAI) as one of the forest resilience indicators. They mention that variations in BAI due to changes in rainfall distribution and intensity can lead to shifts in species composition and overall forest structure, influencing the long-term resilience of these ecosystems.
			Temperature	Fluctuations in temperature can affect the system's robustness inversely and reduce its resilience.	(Hlásny et al., 2021)	The paper considers temperature as a significant factor influencing the resilience of forest aboveground biomass to disturbances.
					(Li et al., 2022)	The study highlights the importance of temperature in forest resilience, as it shapes ecological processes essential for vegetation recovery.

S1. Resilience principles, criteria and sub-criteria

Principles and their definition	Criterion	Explanation	Sub-criterion	Explanation	Examples of the usage in literature	
					References	Explanation
Complex adaptive system (CAS) thinking	Robustness	-	Temperature	-	(Chambers et al., 2023)	In this study, annual mean air temperature, mean air temperature of the coldest month, and Mean of the daily range of air temperature were used as indicators to assess resilience.
					(Soubry et al., 2021)	The authors state that air temperature, water and soil temperature can influence forest resilience.
					(Alfaro-Sánchez et al., 2019)	According to this paper, temperature variability is among the critical factors that newly stablished forests are sensitive and affects the growth and recovery rate of forests.
					(Semeraro et al., 2020)	The study uses LST (land surface temperature) analysis to evaluate resilience after an ENSO event.
			Wind speed	Areas that are exposed to high winds are more vulnerable and less robust. These areas are also more prone to soil erosion.	-	-
			Slope	Slope is relevant to forest resilience because it affects soil stability, water retention, and erosion, all of which influence vegetation recovery after a disturbance. For example, steep slopes typically have lower water storage capacity.	(Arenas-Wong et al., 2023)	According to this study, slope and topography play a significant role in forest resilience to extreme climatic events.
					(Li et al., 2022)	The study investigates the relationship of slope and recovery of disaster-hit forest and shows that non-recovery areas have steeper slope.
					(de la Barrera et al., 2018)	The study notes that certain locations, due to their topography and vegetation, are more susceptible to major wildfires. Steep mountainous terrain can also affect ecosystem resilience, influencing the extent of burned areas and the recovery process.
					(Hlásny et al., 2021)	Considered 18 slope categories.

S1. Resilience principles, criteria and sub-criteria

Principles and their definition	Criterion	Explanation	Sub-criterion	Explanation	Examples of the usage in literature	
					References	Explanation
Complex adaptive system (CAS) thinking	Robustness	-	Slope	-	(Sui et al., 2023)	They also included terrain feature like slope and elevation in their analysis.
					(Bryant et al., 2019)	Slope is relevant to resilience because it influences moisture retention, as reflected in the Topographic Moisture Potential Index (TMPI), which assesses a site's potential to withstand drought based on its topographic features, including slope.
			Site index	Site index is a measure of productivity of the site at a given age. The higher the productivity of a given site, the greater its resilience. This index suggests that sites with lower productivity experience greater drought stress, while higher productive sites are better at recruiting new trees and preserving the pre-disturbance forest type.	(Bryant et al., 2019)	Site index was used as one of the indicators to assess resilience in the face of drought.
			Stand density	Stand density is a measure of the competition for resources based on the average diameter and number of stems in an area. If the competition for resources is not intense among the species, the forest is more resilient.	(Bryant et al., 2019)	Stand density is a crucial factor in assessing forest resilience. It is measured using the Stand Density Index (SDI), which quantifies the level of competition among trees for resources such as light, water, and nutrients. This paper used the criterion to assess resilience in the face of drought.
					(Alfaro-Sánchez et al., 2019)	Considered competition index which is based on the ratio of focal basal area to neighbouring basal area as one of the factors influencing forest sensitivity and resilience.

S1. Resilience principles, criteria and sub-criteria

Principles and their definition	Criterion	Explanation	Sub-criterion	Explanation	Examples of the usage in literature	
					References	Explanation
Complex adaptive system (CAS) thinking	Robustness	-	Stand density	-	(Valipour et al., 2014)	The paper calculates stand density using stem numbers, basal area, and crown cover, noting its indirect role in resilience by balancing tree and ground vegetation productivity in traditionally managed forests, which supports ecosystem sustainability and resistance to environmental pressures.
	Resourcefulness	Resourcefulness is the capacity of the system to identify problems, mobilise resources and establish priorities in times of disturbance.	Access to the system's primary data	Access to regional data (e.g. hydrological data) is a powerful tool for resilience enhancement.	(Turner et al., 2022)	They mention that access to technical assets is one of the key resilience mechanisms.
					(Enoki et al., 2014)	The paper highlights that access to long-term monitoring data enables the evaluation of disturbance impacts and supports the development of effective management and resilience strategies.
			Existence of forest management programmes	These programmes can improve a forest's condition in terms of resilience.	(Raj and Sharma, 2023)	The authors assert that that existence of protected forest areas can be an indicator of resilience.
					(Enoki et al., 2014)	They highlight that the existence of forest management programs, supported by long-term ecological research and monitoring, plays a critical role in enhancing forest resilience by providing valuable data and insights for informed decision-making and effective ecosystem management.
					(de la Mora et al., 2021)	The paper emphasizes the need for a holistic forest management approach that integrates the interactions between social and ecological systems to promote sustainability and resilience.

S1. Resilience principles, criteria and sub-criteria

Principles and their definition	Criterion	Explanation	Sub-criterion	Explanation	Examples of the usage in literature	
					References	Explanation
Complex adaptive systems (CAS) thinking	Resourcefulness	-	Existence of forest management programmes	-	(Schoennagel et al., 2017)	The paper highlights the importance of the forest management practices in increasing the adaptive capacity and resilience of forest in the face of forest fires.
					(Gauthier et al., 2015)	The paper discusses the role of management practice on forest health and resilience, and states that intensive forest management, such as homogenization of forests and fire suppression, can reduce resilience by decreasing biodiversity and altering natural disturbance regimes.
					(Devisscher et al., 2021)	The paper highlights forest management practices that enhance resilience, including age and species diversification, introducing drought-tolerant species, systematic long-term monitoring, wildfire risk management, and improved silviculture techniques.
					(Yang et al., 2024)	This paper investigates the impact of different ecological-engineering project on resilience of subtropical forests in China and underscores the need for management practices to incorporate resilience metrics to ensure sustainability.
			Additional budget allocation for disaster management	Considering such budgets in times of disturbance can increase resourcefulness, provided that the budget is not spent elsewhere.	(Reid et al., 2017)	They underscore the importance of long-term budget allocation and sufficient resources in supporting the resilience and longevity of restored ecosystems.
					(McMillen et al., 2016)	The paper discusses the importance of funding on perpetuation of management plans and how funding is essential for long-term sustainability.

S1. Resilience principles, criteria and sub-criteria

Principles and their definition	Criterion	Explanation	Sub-criterion	Explanation	Examples of the usage in literature	
					References	Explanation
Complex adaptive systems (CAS) thinking	Resourcefulness	-	Existence of hazard (e.g. drought, flood, storm) warning and forecasting systems	These types of systems, if accurate, have a positive effect on resourcefulness.	-	-
			Availability of drought/flood vulnerability maps	The existence of these vulnerability maps can be used to improve resilience more effectively	(Islam et al., 2020)	Landcover maps and human perception were used to create maps for assessing the resilience of ecosystem services to cyclones.
					(Wu and Kim, 2013)	The study employs a fire hazard model, incorporating tree density and fuel loads, to evaluate the likelihood of high-severity wildfires, providing insights into forest resilience and potential ecological regime shifts.
					(de la Barrera et al., 2018)	The paper applies multicriteria analysis to create hazard maps, which enhance resilience by guiding restoration efforts, resource allocation, and impact mitigation, thus aiding in preparation for future hazards.
					(Vega et al., 2016)	The paper emphasizes the importance of understanding fire severity and its impact on forest resilience, particularly by linking vulnerable areas to overall resilience.
					(Haberstroh and Werner, 2022)	The paper suggests that understanding where negative species interaction or severe drought impacts occur could be important to enhance resilience.
			Cooperation between different institutions and organisations in the face of disturbance	The existence of cooperation can improve resourcefulness and resilience.	(Chazdon and Guariguata, 2016)	The paper emphasizes the critical role of diverse stakeholder cooperation, including government, landowners, and community leaders, in initiatives like Brazil's Atlantic Forest Restoration Pact, to create policies, secure land tenure, and provide financial incentives for fostering natural forest regeneration.

S1. Resilience principles, criteria and sub-criteria

Principles and their definition	Criterion	Explanation	Sub-criterion	Explanation	Examples of the usage in literature	
					References	Explanation
Complex adaptive system (CAS) thinking	Resourcefulness	-	Cooperation between different institutions and organisations in the face of disturbance	-	(Nikinmaa et al., 2020)	This paper introduces networks and participation in community organizations as important indicators of social-ecological resilience.
					(Stevens-Rumann et al., 2018)	The paper suggests that adaptive management and involvement of different stakeholders in the management process can help address resilience challenges posed by environmental changes.
					(Nikinmaa et al., 2023)	The paper highlights that stakeholder engagement and cooperation is crucial in resilience assessment as it can provide the means for a more accurate and holistic view of the system which can lead to better decision-making and management outcomes.
					(Steenberg et al., 2019)	The paper discusses the importance of cooperation within different departments and between different governance levels to reach sustainable and resilient urban forests.
					(Charnley et al., 2017)	The paper emphasizes the importance of collaboration with academic, governmental, and private sector partners, particularly the Canadian Forest Service, in establishing and monitoring research plots to inform policy and forest management for enhancing sustainability and resilience in forest ecosystems.

S1. Resilience principles, criteria and sub-criteria

Principles and their definition	Criterion	Explanation	Sub-criterion	Explanation	Examples of the usage in literature	
					References	Explanation
Complex adaptive system (CAS) thinking	Resourcefulness	-	Resolution of property rights	Giving property rights to indigenous communities leads to a resurgence of pride in traditional values and a focus on management of the subsistence resources on which these communities depend. For example, giving priority to local habitants over commercial users can lead to insurance of their livelihood.	(Turner et al., 2022)	Rights and ownership is introduced as one of the resilience mechanisms.
					(Mansourian et al., 2020)	The paper highlights that secure land tenure and property rights are foundational for sustainable management and enhancing resilience.
	Rapidity	Mechanisms that affect the system's rate of recovery can be associated with rapidity. A greater recovery rate correlates with diminished effects of the disruption.	The density of the affected population/species of the system	The higher the density of the system's population, the lower the pace of recovery	(Raj and Sharma, 2023)	The authors underscore that higher population density can significantly impact the resilience of ecosystems by increasing resource pressure, driving land use changes, contributing to pollution, escalating human-wildlife conflicts, and exacerbating climate change.
					(Nikinmaa et al., 2020)	This review paper introduces population density as one of the common indicators for assessing social-ecological resilience.
					(Ferrara et al., 2016)	Population density and population growth rate are considered as factors influencing resilience.
					(Moreno-Fernández et al., 2021)	According to this paper, population dynamics are indeed influential factors in determining the resilience of the forest ecosystem, affecting economic activities, labour availability, community engagement, and the balance between human needs and conservation goals.

S1. Resilience principles, criteria and sub-criteria

Principles and their definition	Criterion	Explanation	Sub-criterion	Explanation	Examples of the usage in literature	
					References	Explanation
Complex adaptive system (CAS) thinking	Rapidity	-	The density of the affected population/species of the system	-	(Cowles et al., 2021)	The paper highlights that increased human density and urbanization introduce unique challenges and disturbances that can impact the resilience of ecosystems.
			Creating a platform for virtual disaster implementation	Virtual disaster implementation and social preparedness in the face of hazards can increase rapidity.	-	-
			Public awareness about environmental issues and demand management	Increasing people's knowledge about adverse impacts of resource scarcity and methods for conserving natural resources can accelerate the rapidity of the system.	(Suarez-Pardo et al., 2022)	The paper considers education and people's ability to access information, which enables adaptation and supports decision-making, as key factors influencing resilience.
					(Nikinmaa et al., 2020)	This paper mentions that education and public awareness is one of the important social-ecological indicators.
					(Turner et al., 2022)	In this study, access to information and education is considered as one of the influential resilience mechanisms.
					(Kelly et al., 2015)	The paper underscores that enhancing public awareness about land degradation and its broader implications can help build consensus, influence political will, encourage sustainable practices, and ultimately strengthen community and ecosystem resilience.
					(Ruiz-Mallén and Corbera, 2013)	According to the findings of the paper it can be inferred that knowledge, awareness, and the intergenerational transfer of ecological wisdom are foundational to maintaining and enhancing resilience within social-ecological systems.

S1. Resilience principles, criteria and sub-criteria

Principles and their definition	Criterion	Explanation	Sub-criterion	Explanation	Examples of the usage in literature	
					References	Explanation
Complex adaptive system (CAS) thinking	Rapidity	-	Hazard severity	Severe droughts can have adverse effects on the system. Under severe droughts, the system can hardly return to its initial state	(Rosenfield et al., 2023)	This paper introduces hazard severity as one of the indicators of resilience.
					(Haberstroh and Werner, 2022)	The paper underscores the importance of understanding how species interactions change with varying drought severity to better manage and enhance forest resilience in the face of increasing drought events due to climate change.
					(Raj and Sharma, 2023)	They used the climate water deficit criterion as part of the resilience and vulnerability assessment process.
					(Guz and Kulakowski, 2020)	In this review, the authors argue that that exposure to past disturbances can enhance the resilience of forests.
			The geopolitical significance of the area	The study area's geopolitical significance can accelerate and retard the pace of recovery. Greater political significance of a given area (e.g. forests at borders of countries) leads to an increased focus on a system's recovery, which in return may increase system rapidity.	-	-
			Average regeneration rate of vegetative cover	Higher regeneration rate can directly improve rapidity.	(Rosenfield et al., 2023)	The paper introduces the recovery rate of forests as a resilience indicator, defined by the speed at which a forest restores its structure, species composition, and ecosystem functions following a disturbance.
					(Chazdon and Guariguata, 2016)	The paper emphasizes that the rate of natural regeneration and the resulting forest structure and composition are crucial for building resilient forest ecosystems.

S1. Resilience principles, criteria and sub-criteria

Principles and their definition	Criterion	Explanation	Sub-criterion	Explanation	Examples of the usage in literature	
					References	Explanation
Complex adaptive systems (CAS) thinking	Rapidity	-	Average growth rate of vegetative cover	Higher growth rate can directly improve rapidity. According to Albiero et al. (2019) growth rate is relevant to resilience as it reflects trees' ability to recover from disturbances.	(Stevens-Rumann et al., 2018)	Regeneration is said to be directly tied to forest resilience because it determines the forests capacity to recover and adapt to disturbances, through sampling the density of seedlings this indicator is measured.
					(Valipour et al., 2014)	According to this study, the low regeneration rate in managed forests negatively impacts their resilience by reducing species richness, creating structural imbalances, and limiting genetic diversity.
					(Vega et al., 2016)	The paper underscores the relevance of regeneration and resilience in the context of understanding and managing Mediterranean terrestrial ecosystems, particularly Aleppo pine forests, after wildfires.
					(Waldron et al., 2023)	the authors highlight that measures of post-disturbance recovery like regeneration rate are commonly used to quantify ecosystem resilience to disturbances
					(de la Mora et al., 2021)	The paper integrates growth rate and resilience into its conceptual model to understand the interactions between social and ecological components and to promote sustainable forest management practices that support ecosystem services and community livelihoods.

S1. Resilience principles, criteria and sub-criteria

Principles and their definition	Criterion	Explanation	Sub-criterion	Explanation	Examples of the usage in literature	
					References	Explanation
Complex adaptive system (CAS) thinking	Rapidity		Average growth rate of vegetative cover	-	(Valipour et al., 2014)	In summary, the growth rate in managed forests directly influences resilience by affecting tree height, basal area, crown cover, size structure, and regeneration. Lower growth rates and poor regeneration hinder the forest's ability to recover from disturbances, maintain ecosystem functions, and adapt to environmental changes, ultimately threatening long-term sustainability.
					(Alfaro-Sánchez et al., 2019)	The paper findings reveal that while higher growth rates can enhance short-term resilience by enabling quick recovery, they can also increase long-term vulnerability to climatic extremes, questioning the sustainability of these forests' ecosystem services, including carbon sequestration.
					(Rosenfield et al., 2023)	The paper considers tree growth as one of the influential indicators for assessing ecological integrity and resilience of secondary forests.
			Tree ring width	Tree rings are indicator of weather conditions, with wider rings indicating humid climate and narrow ones indicating arid climate. Trees having a variety of ring width can exhibit that they have experienced different climatic conditions. Hence, they are more resilient and have a higher recovery rate.	(Albiero et al., 2019)	Tree rings are vital for studying resilience, offering historical insights into tree growth and responses to disturbances like fragmentation and edge effects, helping identify stress and recovery periods.
					(Pardos et al., 2021)	The study uses a large tree ring data base to evaluate different indices including resilience.
					(Che et al., 2023)	The study uses different field data and dendroecological analysis, including tree ring, to evaluate the response and recovery capacity of mixed and pure stands to severe droughts.

S1. Resilience principles, criteria and sub-criteria

Principles and their definition	Criterion	Explanation	Sub-criterion	Explanation	Examples of the usage in literature	
					References	Explanation
Complex adaptive system (CAS) thinking	Rapidity	-	Tree ring width	-	(Anderegg et al., 2020)	The study uses tree-ring data from 1,208 growth chronologies (1900–2015) to analyse how repeated droughts impact tree growth and forest resilience, focusing on growth declines measured by ring-width indices.
					(Gazol et al., 2022)	The paper uses tree-ring analysis to understand the growth trajectories and resilience of Douglas fir under different climatic conditions, particularly in seasonally dry environments.
			Seed banks	Existence of abundant seed banks can ensure the recovery of the forest after disturbance.	(Sun et al., 2013)	The paper highlights the vital role of seed banks for forest resilience as they provide a diverse and abundant seed supply to support vegetation regeneration and recovery after disturbances, and to ensuring ecosystem stability.
					(de Medeiros-Sarmento et al., 2021)	The paper highlights the soil seed bank's role in forest resilience by facilitating natural regeneration, maintaining biodiversity, and enhancing ecosystem services.

S1. Resilience principles, criteria and sub-criteria

Principles and their definition	Criterion	Explanation	Sub-criterion	Explanation	Examples of the usage in literature	
					References	Explanation
Experimentation and learning[6]: the dynamic nature of systems and their inherent uncertainties necessitate a continuous updating of knowledge through experimentation and systematic monitoring. This ongoing learning equips us to adapt effectively to changes and improve our capacity to respond to future challenges.	Information exchange	This criterion investigates the rate that information is exchanged between different stakeholders. In different governing systems stakeholders with greater power and interest have access to more information.	In-degree centrality[7]	Determines the total received intensity of each node. The higher the value of the in-degree centrality, the more accessibility of the given node to the other nodes.	(Suarez-Pardo et al., 2022)	The paper recognizes information distribution as a key variable of resilience, describing it as the mechanisms through which different entities access and provide relevant information.
			Out-degree centrality	Determines the total intensity started from each node. The higher the value of the out-degree centrality of a node, the more significant the centrality role of the node.	(Charnley et al., 2017)	According to the paper, information exchange boosts resilience by enabling learning through the comparison of various natural resource management approaches, helping identify effective strategies. It also supports adaptive management by fostering communication, coordination, and collaboration among stakeholders, strengthening social networks for collective action, and promoting the sharing of research findings with local actors.
			Beta centrality	The centrality of each node (stakeholders) is calculated using the centrality of its adjacent nodes.		
			Betweenness centrality	Determines the mediating role of each stakeholder.		
Encouraging participation: This principle states that to enhance resilience, it is necessary to have a decision-making process that involves the active participation of key stakeholders and incorporates their perspectives.	Interest in cooperation	Stakeholders who are very interested in cooperating (i.e. benefit from cooperation) can enhance the resilience of the system through the active involvement in decision-making process.	Affectability	Indicates how effective stakeholders are when a certain management practice is implemented.	(Cotroneo et al., 2021)	The paper emphasizes that collaboration among organizations is vital for building socio-ecological resilience by fostering adaptive strategies, resolving land conflicts, and shaping public policies.

[6] The criteria mentioned for the following social principles (learning and experimentation, encouraging participation and promoting polycentric governance) are proposed based on the previous works (Ahmadi et al., 2019; Ahmadi et al., 2020; Anamaghi et al., 2023; Behboudian et al., 2024; Behboudian et al., 2023), the proposed criteria have not been implemented to investigate social aspects of the forest resilience. Hence, the provided references merely highlight the importance of the principles themselves in the literature.

[7] Centrality refers to the number of nodes (i.e. stakeholders) adjacent to specific node. In-degree centrality refers to the number of stakeholders that have chosen a specific stakeholder with whom to have an interaction.

S1. Resilience principles, criteria and sub-criteria

Principles and their definition	Criterion	Explanation	Sub-criterion	Explanation	Examples of the usage in literature	
					References	Explanation
Encouraging participation	Interest in cooperation	-	Responsibility	Indicates how responsible stakeholders are for implementing a management scenario.	(Samani et al., 2023)	Overall, the document underscores the importance of collaboration between local communities, conservation organizations, and government agencies to achieve sustainable ecotourism and conservation outcomes in a forest in West Africa.
			Consistency with the nature of the mission	Indicates the consistency of implementing a scenario with stakeholders' mission in the study area.	(Dutra et al., 2022)	It emphasizes that the involvement of stakeholders in the creation and implementation of environmental protection instruments is crucial for enhancing forest resilience.
			Type of organisation	The institutions are classified into three types: protective, developing and intermediate stakeholders.	(Reid et al., 2017)	They emphasize that the interest and active participation of various stakeholders, including organizations, are essential for the long-term success and resilience of restoration projects.
					(Valipour et al., 2014)	The paper highlights the crucial role of local community involvement and traditional knowledge in forest management and suggests ways to integrate these elements into formal conservation efforts to enhance stakeholder participation and forest resilience.
					(Mansourian et al., 2020)	The paper states that active and equitable collaboration across geographic, administrative, and generational scales is seen as key to improving FLR (Forest Landscape Restoration) implementation and enhancing resilience.

S1. Resilience principles, criteria and sub-criteria

Principles and their definition	Criterion	Explanation	Sub-criterion	Explanation	Examples of the usage in literature	
					References	Explanation
Promoting polycentric governance: Polycentricity denotes a governance system characterised by the presence of multiple governing authorities or decision-making centres operating at differing scales.	Comprehensive centrality	If several stakeholders have central roles (i.e. have more power and interest and can influence other stakeholders), the governing system is more polycentric.<br>This principle emphasizes the role of governance systems in sustainable forest management, particularly focusing on the decision-making processes involving internal and external stakeholders at national, regional, and local levels (de la Mora et al., 2021).	Beta centrality	The definitions are the same as the information exchange sub-criteria.	(de la Mora et al., 2021)	This paper mentions that governance enhances resilience in socio-ecological forest systems by promoting adaptive management, inclusive decision-making, and community participation to sustain ecosystems and socio-economic balance
			Betweenness centrality		(Newton, 2011)	According to this paper, institutional diversity can enhance the adaptive capacity of the system, as it has in the case study of new forest in England.
			In-degree centrality		(Reid et al., 2017)	The paper outlines a polycentric governance system where multiple decision-making authorities collaboratively influence ecosystem restoration, enabling diverse inputs and adaptive management to enhance resilience and sustainability.
			Out-degree centrality		(Mansourian et al., 2020)	The paper asserts that polycentric governance facilitates a more inclusive and participatory decision-making process, which is crucial for addressing the trade-offs between ecological integrity and human well-being, thereby enhancing the resilience of both social and ecological systems.

Table S2. Preferred Reporting Items for Systematic reviews and Meta-Analyses extension for Scoping Reviews (PRISMA-ScR) Checklist

SECTION	ITEM	PRISMA-ScR CHECKLIST ITEM	REPORTED ON PAGE #
**TITLE**			
Title	1	Identify the report as a scoping review.	Done
**ABSTRACT**			
Structured summary	2	Provide a structured summary that includes (as applicable): background, objectives, eligibility criteria, sources of evidence, charting methods, results and conclusions that relate to the review questions and objectives.	Abstract
**INTRODUCTION**			
Rationale	3	Describe the rationale for the review in the context of what is already known. Explain why the review questions/objectives lend themselves to a scoping review approach.	Paragraphs 4-8 of the Introduction
Objectives	4	Provide an explicit statement of the questions and objectives being addressed with reference to their key elements (e.g. population or participants, concepts, and context) or other relevant key elements used to conceptualise the review questions and/or objectives.	Last paragraph of Introduction
**METHODS**			
Protocol and registration	5	Indicate whether a review protocol exists; state if and where it can be accessed (e.g. a Web address) and, if available, provide registration information, including the registration number.	-
Eligibility criteria	6	Specify characteristics of the sources of evidence used as eligibility criteria (e.g. years considered, language, and publication status), and provide a rationale.	Section 2.2
Information sources*	7	Describe all information sources in the search (e.g. databases with dates of coverage and contact with authors to identify additional sources), as well as the date the most recent search was executed.	Section 2.1
Search	8	Present the full electronic search strategy for at least one database, including any limits used, such that it could be repeated.	Section 2.1
Selection of sources of evidence†	9	State the process for selecting sources of evidence (i.e. screening and eligibility) included in the scoping review.	Section 2.2
Data charting process‡	10	Describe the methods of charting data from the included sources of evidence (e.g. calibrated forms or forms that have been tested by the team before their use, and whether data charting was done independently or in duplicate) and any processes for obtaining and confirming data from investigators.	Section 2.1
Data items	11	List and define all variables for which data were sought and any assumptions and simplifications made.	Section 2.1

Table S2. Preferred Reporting Items for Systematic reviews and Meta-Analyses extension for Scoping Reviews (PRISMA-ScR) Checklist

SECTION	ITEM	PRISMA-ScR CHECKLIST ITEM	REPORTED ON PAGE #
Critical appraisal of individual sources of evidence§	12	If done, provide a rationale for conducting a critical appraisal of included sources of evidence; describe the methods used and how this information was used in any data synthesis (if appropriate).	Section 2.4
Synthesis of results	13	Describe the methods of handling and summarising the data that were charted.	Section 2.3
**RESULTS**			
Selection of sources of evidence	14	Give numbers of sources of evidence screened, assessed for eligibility and included in the review, with reasons for exclusions at each stage, ideally using a flow diagram.	First paragraph of Results+ figure 1
Characteristics of sources of evidence	15	For each source of evidence, present characteristics for which data were charted and provide the citations.	Sections 3.1-3.5
Critical appraisal within sources of evidence	16	If done, present data on critical appraisal of included sources of evidence (see item 12).	-
Results of individual sources of evidence	17	For each included source of evidence, present the relevant data that were charted that relate to the review questions and objectives.	Sections 3.1-3.5
Synthesis of results	18	Summarise and/or present the charting results as they relate to the review questions and objectives.	Sections 3.1-3.5
**DISCUSSION**			
Summary of evidence	19	Summarise the main results (including an overview of concepts, themes, and types of evidence available), link to the review questions and objectives, and consider the relevance to key groups.	First paragraphs of Discussion and sub-sections 4.1 and 4.2
Limitations	20	Discuss the limitations of the scoping review process.	Second paragraph of the Discussion
Conclusions	21	Provide a general interpretation of the results with respect to the review questions and objectives, as well as potential implications and/or next steps.	Conclusion section
**FUNDING**			
Funding	22	Describe sources of funding for the included sources of evidence, as well as sources of funding for the scoping review. Describe the role of the funders of the scoping review.	Funding

JBI = Joanna Briggs Institute; PRISMA-ScR = Preferred Reporting Items for Systematic reviews and Meta-Analyses extension for Scoping Reviews.

* Where *sources of evidence* (see second footnote) are compiled from, such as bibliographic databases, social media platforms, and websites.

† A more inclusive/heterogeneous term used to account for the different types of evidence or data sources (e.g. quantitative and/or qualitative research, expert opinion, and policy documents) that may be eligible in a scoping review as opposed to only studies. This is not to be confused with *information sources* (see first footnote).

‡ The frameworks by Arksey and O'Malley (6) and Levac and colleagues (7) and the JBI guidance (4, 5) refer to the process of data extraction in a scoping review as data charting.

§ The process of systematically examining research evidence to assess its validity, results and relevance before using it to inform a decision. This term is used for items 12 and 19 instead of "risk of bias" (which is more applicable to systematic reviews of interventions) to include and acknowledge the various sources of evidence that may be used in a scoping review (e.g. quantitative and/or qualitative research, expert opinion and policy document).

From: Tricco AC, Lillie E, Zarin W, O'Brien KK, Colquhoun H, Levac D, et al. PRISMA Extension for Scoping Reviews (PRISMAScR): Checklist and Explanation. Ann Intern Med. 2018;169:467–473. doi: 10.7326/M18-0850.

List of reviewed studies

Abdullah, M. M., Al-Ali, Z. M., Blanton, A., Charabi, Y., Abulibdeh, A., Al-Awadhi, T., Srinivasan, S., Fadda, E., and Mohan, M. (2024). UAVs for improving seasonal vegetation assessment in arid environments. Frontiers in Environmental Science, 12, 5, Article 1366712.

Akamani, K.and Hall, T. E. (2019). Scale and co-management outcomes: assessing the impact of collaborative forest management on community and household resilience in Ghana. Heliyon, 5(1), 29, Article e01125.

Albiero, A., Venegas-González, A., Botosso, P. C., Roig, F. A., Camargo, J. L. C., and Tomazello, M. (2019). What is the temporal extension of edge effects on tree growth dynamics? A dendrochronological approach model using Scleronema micranthum (Ducke) Ducke trees of a fragmented forest in the Central Amazon. Ecological Indicators, 101, 133-142.

Alfaro-Sánchez, R., Jump, A. S., Pino, J., Díez-Nogales, O., and Espelta, J. M. (2019). Land use legacies drive higher growth, lower wood density and enhanced climatic sensitivity in recently established forests. Agricultural and Forest Meteorology, 276, 11, Article 107630.

Almenar, J. B., Petucco, C., Sonnemann, G., Geneletti, D., Elliot, T., and Rugani, B. (2023). Modelling the net environmental and economic impacts of urban nature-based solutions by combining ecosystem services, system dynamics and life cycle thinking: An application to urban forests. Ecosystem Services, 60, 21, Article 101506.

Altamirano, A., Gonzalez-Suhr, C., Marien, C., Catalan, G., Miranda, A., Prado, M., Tits, L., Vieli, L., and Meli, P. (2020). Landscape Disturbance Gradients: The Importance of the Type of Scene When Evaluating Landscape Preferences and Perceptions. Land, 9(9), 23, Article 306.

Anderegg, W. R. L., Konings, A. G., Trugman, A. T., Yu, K. L., Bowling, D. R., Gabbitas, R., Karp, D. S., Pacala, S., Sperry, J. S., Sulman, B. N., and Zenes, N. (2018). Hydraulic diversity of forests regulates ecosystem resilience during drought. Nature, 561(7724), 538-+.

Anderegg, W. R. L., Trugman, A. T., Badgley, G., Konings, A. G., and Shaw, J. (2020). Divergent forest sensitivity to repeated extreme droughts. Nature Climate Change, 10(12), 1091-U1019.

Antwi, E. K., Mensah, R., Attua, E. M., Yiran, G., Boakye-Danquah, J., Ametepe, R., and Boadi, D. A. (2014, Aug 06-07). Assessing Land and Ecosystem Management at the Local Level in the Savannah Ecological Zone and the Implications for Sustainability.Science for Sustainable Societies [Strategies for building resilience against climate and ecosystem changes in sub-saharan africa]. 1st International Conference on Enhancing Resilience to Climate and Ecosystem Changes in Semi-Arid Africa, Univ Dev Studies, Tamale, GHANA.

Arenas-Wong, R. A., Robles-Morúa, A., Bojórquez, A., Martínez-Yrízar, A., Yépez, E. A., and Alvarez-Yépiz, J. C. (2023). Climate-induced changes to provisioning ecosystem services in rural socioecosystems in Mexico. Weather and Climate Extremes, 41, 13, Article 100583.

Arnold, J. S., Koro-Ljungberg, M., and Bartels, W. L. (2012). Power and Conflict in Adaptive Management: Analyzing the Discourse of Riparian Management on Public Lands. Ecology and Society, 17(1), 12, Article 19.

Arroyo-Rodríguez, V., Rito, K. F., Farfán, M., Navía, I. C., Mora, F., Arreola-Villa, F., Balvanera, P., Bongers, F., Castellanos-Castro, C., Catharino, E. L. M., Chazdon, R. L., Dupuy-Rada, J. M., Ferguson, B. G., Foster, P. F., González-Valdivia, N., Griffith, D. M., Hernández-Stefanoni, J. L., Jakovac, C. C., Junqueira, A. B., Jong, B. H. J., Letcher, S. G., May-Pat, F., Meave, J. A., Ochoa-Gaona, S., Meirelles, G. S., Muñiz-Castro, M. A., Muñoz, R., Powers, J. S., Rocha, G. P. E., Rosário, R. P. G., Santos, B. A., Simon, M. F., Tabarelli, M., Tun-Dzul, F., van den Berg, E., Vieira, D. L. M., Williams-Linera, G., and Martínez-Ramos, M. (2023). Landscape-scale forest cover drives the predictability of forest regeneration across the Neotropics. Proceedings of the Royal Society B-Biological Sciences, 290(1990), 9, Article 20222203.

Asbjornsen, H., Hernandez-Santana, V., Liebman, M., Bayala, J., Chen, J., Helmers, M., Ong, C. K., and Schulte, L. A. (2014). Targeting perennial vegetation in agricultural landscapes for enhancing ecosystem services [Review]. Renewable Agriculture and Food Systems, 29(2), 101-125.

Atchadé, A. J., Kanda, M., Folega, F., Yédomonhan, H., Dourma, M., Wala, K., and Akpagana, K. (2023). Trees Diversity and Species with High Ecological Importance for a Resilient Urban Area: Evidence from Cotonou City (West Africa). Climate, 11(9), 16, Article 182.

Atchison, R. A.and Lucky, A. (2022). Diversity and Resilience of Seed-Removing Ant Species in Longleaf Sandhill to Frequent Fire. Diversity-Basel, 14(12), 20, Article 1012.

Atwell, R. C., Schulte, L. A., and Westphal, L. M. (2011). Tweak, Adapt, or Transform: Policy Scenarios in Response to Emerging Bioenergy Markets in the US Corn Belt. Ecology and Society, 16(1), 15, Article 10.

Baltzer, J. L., Day, N. J., Walker, X. J., Greene, D., Mack, M. C., Alexander, H. D., Arseneault, D., Barnes, J., Bergeron, Y., Boucher, Y., Bourgeau-Chavez, L., Brown, C. D., Carrière, S., Howard, B. K., Gauthier, S., Parisien, M. A., Reid, K. A., Rogers, B. M., Roland, C., Sirois, L., Stehn, S., Thompson, D. K., Turetsky, M. R., Veraverbeke, S., Whitman, E., Yang, J., and Johnstone, J. F. (2021). Increasing fire

and the decline of fire adapted black spruce in the boreal forest. Proceedings of the National Academy of Sciences of the United States of America, 118(45), 9, Article e2024872118.

Bamwesigye, D., Fialova, J., Kupec, P., Lukaszkiewicz, J., and Fortuna-Antoszkiewicz, B. (2021). Forest Recreational Services in the Face of COVID-19 Pandemic Stress. Land, 10(12), 19, Article 1347.

Beier, C. M., Patterson, T. M., and Chapin, F. S. (2008). Ecosystem services and emergent vulnerability in managed ecosystems: A geospatial decision-support tool. Ecosystems, 11(6), 923-938.

Benítez, G., Ruelas-Monjardín, L. C., Von Thaden, J., Acosta-Rosado, I., Alvarado-Castillo, G., and Equihua, M. (2023). Carbon storage in a peri-urban neotropical forest: Assessing its potential and patterns of change over half a century. Urban Forestry & Urban Greening, 86, 11, Article 128009.

Besi, E. E., Mustafa, M., Yong, C. S. Y., and Go, R. (2023). Deforestation Impacts on Diversity of Orchids with Inference on the Conservation Initiatives: Malaysia Case Study. Botanical Review, 89(4), 386-420.

Biagioni, S., Wündsch, M., Haberzettl, T., and Behling, H. (2015). Assessing resilience/sensitivity of tropical mountain rainforests towards climate variability of the last 1500 years: The long-term perspective at Lake Kalimpaa (Sulawesi, Indonesia). Review of Palaeobotany and Palynology, 213, 42-53.

Bieling, C., Plieninger, T., and Schaich, H. (2013). Patterns and causes of land change: Empirical results and conceptual considerations derived from a case study in the Swabian Alb, Germany. Land Use Policy, 35, 192-203.

Biggs, R., Clements, H. S., Cumming, G. S., Cundill, G., De Vos, A., Hamann, M., Luvuno, L., Roux, D. J., Selomane, O., Blanchard, R., Cockburn, J., Dziba, L., Esler, K. J., Fabricius, C., Henriksson, R., Kotschy, K., Lindborg, R., Masterson, V. A., Nel, J. L., O'Farrell, P., Palmer, C. G., Pereira, L., Pollard, S., Preiser, R., Scholes, R. J., Shackleton, C., Shackleton, S., Sitas, N., Slingsby, J. A., Spierenburg, M., Tengö, M., and Reyers, B. (2022). Social-ecological change: insights from the Southern African Program on Ecosystem Change and Society. Ecosystems and People, 18(1), 447-468.

Bone, C., Moseley, C., Vinyeta, K., and Bixler, R. P. (2016). Employing resilience in the United States Forest Service. Land Use Policy, 52, 430-438.

Borghi, C., Francini, S., McRoberts, R. E., Parisi, F., Lombardi, F., Nocentini, S., Maltoni, A., Travaglini, D., and Chirici, G. (2024). Country-wide assessment of biodiversity, naturalness and old-growth status using national forest inventory data. European Journal of Forest Research, 143(1), 271-303.

Brando, P. M., Silverio, D., Maracahipes-Santos, L., Oliveira-Santos, C., Levick, S. R., Coe, M. T., Migliavacca, M., Balch, J. K., Macedo, M. N., Nepstad, D. C., Maracahipes, L., Davidson, E., Asner, G., Kolle, O., and Trumbore, S. (2019). Prolonged tropical forest degradation due to compounding disturbances: Implications for CO_2 and H_2O fluxes. Global Change Biology, 25(9), 2855-2868.

Brosi, B. J., Daily, G. C., Shih, T. M., Oviedo, F., and Durán, G. (2008). The effects of forest fragmentation on bee communities in tropical countryside. Journal of Applied Ecology, 45(3), 773-783.

Bryant, T., Waring, K., Meador, A. S., and Bradford, J. B. (2019). A Framework for Quantifying Resilience to Forest Disturbance. Frontiers in Forests and Global Change, 2, 14, Article 56.

Buma, B.and Wessman, C. A. (2013). Forest resilience, climate change, and opportunities for adaptation: A specific case of a general problem. Forest Ecology and Management, 306, 216-225.

Butler, J. R. A., Wong, G. Y., Metcalfe, D. J., Honzák, M., Pert, P. L., Rao, N., van Grieken, M. E., Lawson, T., Bruce, C., Kroon, F. J., and Brodie, J. E. (2013). An analysis of trade-offs between multiple ecosystem services and stakeholders linked to land use and water quality management in the Great Barrier Reef, Australia. Agriculture Ecosystems & Environment, 180, 176-191.

Cajaiba, R. L., Périco, E., da Silva, W. B., Caron, E., Buss, B. C., Dalzochio, M., and Santos, M. (2020). Are primary forests irreplaceable for sustaining Neotropical landscapes' biodiversity and functioning? Contributions for restoration using ecological indicators. Land Degradation & Development, 31(4), 508-517.

Campbell, L. K., Svendsen, E. S., Sonti, N. F., and Johnson, M. L. (2016). A social assessment of urban parkland: Analyzing park use and meaning to inform management and resilience planning. Environmental Science & Policy, 62, 34-44.

Cannon, J. B., Gannon, B. M., Feinstein, J. A., Padley, E. A., and Metz, L. J. (2020). Simulating spatial complexity in dry conifer forest restoration: implications for conservation prioritization and scenario evaluation. Landscape Ecology, 35(10), 2301-2319.

Cantarello, E., Jacobsen, J. B., Lloret, F., and Lindner, M. (2024). Shaping and enhancing resilient forests for a resilient society. Ambio, 53(8), 1095-1108.

Cantarello, E., Newton, A. C., Martin, P. A., Evans, P. M., Gosal, A., and Lucash, M. S. (2017). Quantifying resilience of multiple ecosystem services and biodiversity in a temperate forest landscape. Ecology and Evolution, 7(22), 9661-9675.

Caputo, J., Beier, C. M., Groffman, P. M., Burns, D. A., Beall, F. D., Hazlett, P. W., and Yorks, T. E. (2016). Effects of Harvesting Forest Biomass on Water and Climate Regulation Services: A Synthesis of Long-Term Ecosystem Experiments in Eastern North America. Ecosystems, 19(2), 271-283.

Carlsson, L.and Lazdinis, M. (2004). Institutional frameworks for sustainability? A comparative analysis of the forest sectors of Russia and the Baltic states. Ambio, 33(6), 366-370.

Carrillo-Saucedo, S. M.and Gavito, M. E. (2020). Resilience of soil aggregation and exocellular enzymatic functions associated with arbuscular mycorrhizal fungal communities along a successional gradient in a tropical dry forest. Mycorrhiza, 30(1), 109-120.

Chambers, J. C., Brown, J. L., Bradford, J. B., Board, D. I., Campbell, S. B., Clause, K. J., Hanberry, B., Schlaepfer, D. R., and Urza, A. K. (2023). New indicators of ecological resilience and invasion resistance to support prioritization and management in the sagebrush biome, United States. Frontiers in Ecology and Evolution, 10, 17, Article 1009268.

Chapin, F. S., Robards, M. D., Huntington, H. P., Johnstone, J. E., Trainor, S. E., Kofinas, G. P., Ruess, R. W., Fresco, N., Natcher, D. C., and Naylor, R. L. (2006). Directional changes in ecological communities and social-ecological systems: A framework for prediction based on Alaskan examples. American Naturalist, 168(6), S36-S49.

Chapin, J., Abrams, J., Timberlake, T. J., Schultz, C., Evans, A. M., and Fernández-Giménez, M. (2021). Operationalizing Resilience on US National Forestlands: A Quantitative Analysis of Environmental Impact Statements. Society & Natural Resources, 34(10), 1394-1411.

Charnley, S., Spies, T. A., Barros, A. M. G., White, E. M., and Olsen, K. A. (2017). Diversity in forest management to reduce wildfire losses: implications for resilience. Ecology and Society, 22(1), 25, Article 22.

Chazdon, R. L.and Guariguata, M. R. (2016). Natural regeneration as a tool for large-scale forest restoration in the tropics: prospects and challenges. Biotropica, 48(6), 716-730.

Che, C. W., Xiao, S. C., Peng, X. M., Ding, A. J., and Su, J. R. (2023). Radial growth of Korshinsk peashrub and its response to drought in different sub-arid climate regions of northwest China. Journal of Environmental Management, 326, 10, Article 116708.

Che, C. W., Xiao, S. C., Peng, X. M., Su, J. R., and Ding, A. J. (2023). Mixed of arbor-shrub significantly enhanced the drought stress adaptive capacity of plantation forests-An interpretation based on dendroecology. Journal of Hydrology, 623, 9, Article 129785.

Chuvieco, E., Yebra, M., Martino, S., Thonicke, K., Gómez-Giménez, M., San-Miguel, J., Oom, D., Velea, R., Mouillot, F., Molina, J. R., Miranda, A. I., Lopes, D., Salis, M., Bugaric, M., Sofiev, M., Kadantsev, E., Gitas, I. Z., Stavrakoudis, D., Eftychidis, G., Bar-Massada, A., Neidermeier, A., Pampanoni, V., Pettinari, M. L., Arrogante-Funes, F., Ochoa, C., Moreira, B., and Viegas, D. (2023). Towards an Integrated Approach to Wildfire Risk Assessment: When, Where, What and How May the Landscapes Burn. Fire-Switzerland, 6(5), 60, Article 215.

Colyn, R. B., Smith, D. A. E., Smith, Y. C. E., Smit-Robinson, H., and Downs, C. T. (2020). Predicted distributions of avian specialists: A framework for conservation of endangered forests under future climates. Diversity and Distributions, 26(6), 652-667.

Connolly, J. J., Svendsen, E. S., Fisher, D. R., and Campbell, L. K. (2013). Organizing urban ecosystem services through environmental stewardship governance in New York City. Landscape and Urban Planning, 109(1), 76-84.

Cotroneo, S. M., Jacobo, E. J., and Brassiolo, M. M. (2021). Degradation processes and adaptive strategies in communal forests of Argentine dry Chaco. Integrating stakeholder knowledge and perceptions. Ecosystems and People, 17(1), 507-522.

Cottrell, S., Mattor, K. M., Morris, J. L., Fettig, C. J., McGrady, P., Maguire, D., James, P. M. A., Clear, J., Wurtzebach, Z., Wei, Y., Brunelle, A., Western, J., Maxwell, R., Rotar, M., Gallagher, L., and Roberts, R. (2020). Adaptive capacity in social-ecological systems: a framework for addressing bark beetle disturbances in natural resource management. Sustainability Science, 15(2), 555-567.

Cowles, J., Templeton, L., Battles, J. J., Edmunds, P. J., Carpenter, R. C., Carpenter, S. R., Nelson, M. P., Cleavitt, N. L., Fahey, T. J., Groffman, P. M., Sullivan, J. H., Neel, M. C., Hansen, G. J. A., Hobbie, S., Holbrook, S. J., Kazanski, C. E., Seabloom, E. W., Schmitt, R. J., Stanley, E. H., Tepley, A. J., van Doorn, N. S., and Vander Zanden, J. M. (2021). Resilience: insights from the US LongTerm Ecological Research Network. Ecosphere, 12(5), 31, Article e03434.

Crous, C. J., Malan, F. S., and Wingfield, M. J. (2016). Securing African forests for future drier climates: applying ecophysiology in tree improvement [Review]. Southern Forests-a Journal of Forest Science, 78(4), 241-254.

Danneyrolles, V., Valeria, O., Djerboua, I., Gauthier, S., and Bergeron, Y. (2020). How Initial Forest Cover, Site Characteristics and Fire Severity Drive the Dynamics of the Southern Boreal Forest. Remote Sensing, 12(23), 11, Article 3957.

Daume, S., Albert, M., and von Gadow, K. (2014). Forest monitoring and social media - Complementary data sources for ecosystem surveillance? Forest Ecology and Management, 316, 9-20.

de la Barrera, F., Barraza, F., Favier, P., Ruiz, V., and Quense, J. (2018). Megafires in Chile 2017: Monitoring multiscale environmental impacts of burned ecosystems. Science of the Total Environment, 637, 1526-1536.

de la Mora, G. D., Sánchez-Nupan, L. O., Castro-Torres, B., and Galicia, L. (2021). Sustainable Community Forest Management in Mexico: An Integrated Model of Three Socio-ecological Frameworks. Environmental Management, 68(6), 900-913.

de Medeiros-Sarmento, P. S., Ferreira, L. V., and Gastauer, M. (2021). Natural regeneration triggers compositional and functional shifts in soil seed banks. Science of the Total Environment, 753, 9, Article 141934.

de Vos, A., Biggs, R., and Preiser, R. (2019). Methods for understanding social-ecological systems: a review of place-based studies [Review]. Ecology and Society, 24(4), 19, Article 16.

Delgado-Serrano, M. D., Escalante, R., and Basurto, S. (2015). Is the community-based management of natural resources inherently linked to resilience? An analysis of the Santiago Comaltepec community (Mexico). Ager-Revista De Estudios Sobre Despoblacion Y Desarrollo Rural(18), 91-114.

Depante, M., Morison, M. Q., Petrone, R. M., Devito, K. J., Kettridge, N., and Waddington, J. M. (2019). Hydraulic redistribution and hydrological controls on aspen transpiration and establishment in peatlands following wildfire. Hydrological Processes, 33(21), 2714-2728.

Devisscher, T., Spies, J., and Griess, V. C. (2021). Time for change: Learning from community forests to enhance the resilience of multi-value forestry in British Columbia, Canada. Land Use Policy, 103, 16, Article 105317.

Ding, Y.and Zang, R. G. (2021). Effects of thinning on the demography and functional community structure of a secondary tropical lowland rain forest. Journal of Environmental Management, 279, 9, Article 111805.

Dinis, I.and Simoes, O. (2021). Resilience in Retrospective: The Trajectory of Agro-Pastoral Systems in the Centro Region of Portugal. Sustainability, 13(9), 29, Article 5089.

dos Santos, C. R., de Freitas, R. R., Costa, R. N. E., and Pimenta, L. H. F. (2021). Ecosystem-based disaster management in the coastal zone: Governance and public engagement after fires in a state park in southern Brazil. International Journal of Disaster Risk Reduction, 63, 12, Article 102449.

Drillet, Z., Fung, T. K., Leong, R. A. T., Sachidhanandam, U., Edwards, P., and Richards, D. (2020). Urban Vegetation Types are Not Perceived Equally in Providing Ecosystem Services and Disservices. Sustainability, 12(5), 14, Article 2076.

Dutra, D. J., Elmiro, M. A. T., and Ribeiro, S. M. C. (2022). Association between forest resources and water availability: temporal analysis of the Serra Azul stream sub-basin. Anais Da Academia Brasileira De Ciencias, 94(3), 15, Article e20201289.

Dymond, C. C., Tedder, S., Spittlehouse, D. L., Raymer, B., Hopkins, K., McCallion, K., and Sandland, J. (2014). Diversifying managed forests to increase resilience. Canadian Journal of Forest Research, 44(10), 1196-1205.

Ebel, B. A.and Martin, D. A. (2017). Meta-analysis of field-saturated hydraulic conductivity recovery following wildland fire: Applications for hydrologic model parameterization and resilience assessment. Hydrological Processes, 31(21), 3682-3696.

Ekblom, A., Gillson, L., Risberg, J., Holmgren, K., and Chidoub, Z. (2012). Rainfall variability and vegetation dynamics of the lower Limpopo Valley, Southern Africa, 500 AD to present. Palaeogeography Palaeoclimatology Palaeoecology, 363, 69-78.

Ellison, A. M., Felson, A. J., and Friess, D. A. (2020). Mangrove Rehabilitation and Restoration as Experimental Adaptive Management. Frontiers in Marine Science, 7, 19, Article 327.

Enoki, T., Nakashizuka, T., Nakano, S., Miki, T., Lin, Y. P., Nakaoka, M., Mizumachi, E., and Shibata, H. (2014). Progress in the 21st century: a Roadmap for the Ecological Society of Japan. Ecological Research, 29(3), 357-368.

Enrica, G., Josep, P. R., Amanda, J. A., Garry, P., Albert, N., Anna, R. P., and Josep, V. S. (2023). Landscape features shape people's perception of ecosystem service supply areas. Ecosystem Services, 64, 13, Article 101561.

Ernstson, H., Barthel, S., Andersson, E., and Borgström, S. T. (2010). Scale-Crossing Brokers and Network Governance of Urban Ecosystem Services: The Case of Stockholm. Ecology and Society, 15(4), 25, Article 28.

Estevo, C. A., Nagy-Reis, M. B., and Silva, W. R. (2017). Urban parks can maintain minimal resilience for Neotropical bird communities. Urban Forestry & Urban Greening, 27, 84-89.

Faivre, N., Sgobbi, A., Happaerts, S., Raynal, J., and Schmidt, L. (2018). Translating the Sendai Framework into action: The EU approach to ecosystem-based disaster risk reduction. International Journal of Disaster Risk Reduction, 32, 4-10.

Fang, O. Y.and Zhang, Q. B. (2019). Tree resilience to drought increases in the Tibetan Plateau. Global Change Biology, 25(1), 245-253.

Farley, J.and Voinov, A. (2016). Economics, socio-ecological resilience and ecosystem services. Journal of Environmental Management, 183, 389-398.

Fathi-Taperasht, A., Shafizadeh-Moghadam, H., Sadian, A., Xu, T. T., and Nikoo, M. R. (2023). Drought-induced vulnerability and resilience of different land use types using time series of MODIS-based indices. International Journal of Disaster Risk Reduction, 91, 13, Article 103703.

Fedele, G., Locatelli, B., and Djoudi, H. (2017). Mechanisms mediating the contribution of ecosystem services to human well-being and resilience. Ecosystem Services, 28, 43-54.

Ferrara, A., Kelly, C., Wilson, G. A., Nolè, A., Mancino, G., Bajocco, S., and Salvati, L. (2016). Shaping the role of 'fast' and 'slow' drivers of change in forest-shrubland socio-ecological systems. Journal of Environmental Management, 169, 155-166.

Field, J. P., Breshears, D. D., Bradford, J. B., Law, D. J., Feng, X., and Allen, C. D. (2020). Forest Management Under Megadrought: Urgent Needs at Finer Scale and Higher Intensity. Frontiers in Forests and Global Change, 3, 10, Article 502669.

Field, R. D.and Parrott, L. (2022). Mapping the functional connectivity of ecosystem services supply across a regional landscape. Elife, 11, 41, Article e69395.

Fischer, J., Bergsten, A., Dorresteijn, I., Hanspach, J., Hylander, K., Jiren, T. S., Manlosa, A. O., Rodrigues, P., Schultner, J., Senbeta, F., and Shumi, G. (2021). A social-ecological assessment of food security and biodiversity conservation in Ethiopia. Ecosystems and People, 17(1), 400-410.

Flood, S., Cradock-Henry, N. A., Blackett, P., and Edwards, P. (2018). Adaptive and interactive climate futures: systematic review of 'serious games' for engagement and decision-making [Review]. Environmental Research Letters, 13(6), 20, Article 063005.

Flores, B. M.and Staal, A. (2022). Feedback in tropical forests of the Anthropocene [Review]. Global Change Biology, 28(17), 5041-5061.

Frazier, A. E., Renschler, C. S., and Miles, S. B. (2013). Evaluating post-disaster ecosystem resilience using MODIS GPP data. International Journal of Applied Earth Observation and Geoinformation, 21, 43-52.

Frei, B., Queiroz, C., Chaplin-Kramer, B., Andersson, E., Renard, D., Rhemtulla, J. M., and Bennett, E. M. (2020). A brighter future: Complementary goals of diversity and multifunctionality to build resilient agricultural landscapes. Global Food Security-Agriculture Policy Economics and Environment, 26, 11, Article 100407.

Freudenberger, L., Hobson, P. R., Schluck, M., and Ibisch, P. L. (2012). A global map of the functionality of terrestrial ecosystems. Ecological Complexity, 12, 13-22.

Galicia-Gallardo, A. P., Ceccon, E., Castillo, A., and González-Esquivel, C. E. (2023). An Integrated Assessment of Social-ecological Resilience in Me′Phaa Indigenous Communities in Southern Mexico. Human Ecology, 51(1), 151-164.

Garate-Quispe, J., Herrera-Machaca, M., Auquipata, V. P., Aguirre, G. A., Quispe, S. B., and Carpio-Vargas, E. E. (2024). Resilience of Aboveground Biomass of Secondary Forests Following the Abandonment of Gold Mining Activity in the Southeastern Peruvian Amazon. Diversity-Basel, 16(4), 22, Article 233.

García, C. (2019). From ecological indicators to ecological functioning: Integrative approaches to seize on ecological, climatic and socio-economic databases. Ecological Indicators, 107, 6, Article 105612.

Gauthier, S., Bernier, P., Kuuluvainen, T., Shvidenko, A. Z., and Schepaschenko, D. G. (2015). Boreal forest health and global change [Review]. Science, 349(6250), 819-822.

Gazol, A., Valeriano, C., Cantero, A., Vergarechea, M., and Camarero, J. J. (2022). Douglas Fir Growth Is Constrained by Drought: Delineating the Climatic Limits of Timber Species under Seasonally Dry Conditions. Forests, 13(11), 16, Article 1796.

Gillson, L., Whitlock, C., and Humphrey, G. (2019). Resilience and fire management in the Anthropocene. Ecology and Society, 24(3), 14, Article 14.

Gómez-Baggethun, E., Mingorría, S., Reyes-García, V., Calvet, L., and Montes, C. (2010). Traditional Ecological Knowledge Trends in the Transition to a Market Economy: Empirical Study in the Donana Natural Areas. Conservation Biology, 24(3), 721-729.

Gonzales, F. N., Craven, D., and Armesto, J. J. (2023). Islands in the mist: A systematic review of the coastal lomas of South America [Review]. Journal of Arid Environments, 211, 16, Article 104942.

González-Cruz, G., García-Frapolli, E., Casas, A., and Dupuy, J. M. (2015). Responding to disturbances: lessons from a Mayan social-ecological system. International Journal of the Commons, 9(2), 831-850.

Gonzalez-Redin, J., Gordon, I. J., Polhill, J. G., Dawson, T. P., and Hill, R. (2024). Navigating Sustainability: Revealing Hidden Forces in Social-Ecological Systems. Sustainability, 16(3), 23, Article 1132.

Gorman, D.and Turra, A. (2016). The role of mangrove revegetation as a means of restoring macrofaunal communities along degraded coasts. Science of the Total Environment, 566, 223-229.

Graça, M. S., Gonçalves, J. F., Alves, P. J. M., Nowak, D. J., Hoehn, R., Ellis, A., Farinha-Marques, P., and Cunha, M. (2017). Assessing mismatches in ecosystem services proficiency across the urban fabric of Porto (Portugal): The influence of structural and socioeconomic variables. Ecosystem Services, 23, 82-93.

Guz, J.and Kulakowski, D. (2020). Forests in the Anthropocene. Annals of the American Association of Geographers, 111(3), 869-879.

Guzy, M. R., Smith, C. L., Bolte, J. P., Hulse, D. W., and Gregory, S. V. (2008). Policy Research Using Agent-Based Modeling to Assess Future Impacts of Urban Expansion into Farmlands and Forests. Ecology and Society, 13(1), 38, Article 37.

Haberstroh, S.and Werner, C. (2022). The role of species interactions for forest resilience to drought [Review]. Plant Biology, 24(7), 1098-1107.

Haga, C., Hotta, W., Inoue, T., Matsui, T., Aiba, M., Owari, T., Suzuki, S. N., Shibata, H., and Morimoto, J. (2022). Modeling Tree Recovery in Wind-Disturbed Forests with Dense Understory Species under Climate Change. Ecological Modelling, 472, 15, Article 110072.

Hahn, T., Eggers, J., Subramanian, N., Caicoya, A. T., Uhl, E., and Snäll, T. (2021). Specified resilience value of alternative forest management adaptations to storms. Scandinavian Journal of Forest Research, 36(7-8), 585-597.

Haider, L. J., Neusel, B., Peterson, G. D., and Schlüter, M. (2019). Past management affects success of current joint forestry management institutions in Tajikistan. Environment Development and Sustainability, 21(5), 2183-2224.

Hajjar, R., Engbring, G., and Kornhauser, K. (2021). The impacts of REDD plus on the social-ecological resilience of community forests. Environmental Research Letters, 16(2), 10, Article 024001.

Hamel, P., Hamann, M., Kuiper, J. J., Andersson, E., Arkema, K. K., Silver, J. M., Daily, G. C., and Guerry, A. D. (2021). Blending Ecosystem Service and Resilience Perspectives in Planning of Natural Infrastructure: Lessons from the San Francisco Bay Area. Frontiers in Environmental Science, 9, 13, Article 601136.

Hansen, W. D. (2014). Generalizable principles for ecosystem stewardship-based management of social-ecological systems: lessons learned from Alaska. Ecology and Society, 19(4), 13, Article 13.

Hapsari, K. A., Biagioni, S., Jennerjahn, T. C., Reimer, P., Saad, A., Sabiham, S., and Behling, H. (2018). Resilience of a peatland in Central Sumatra, Indonesia to past anthropogenic disturbance: Improving conservation and restoration designs using palaeoecology. Journal of Ecology, 106(6), 2473-2490.

Haq, S. M., Pieroni, A., Bussmann, R. W., Abd-ElGawad, A. M., and El-Ansary, H. O. (2023). Integrating traditional ecological knowledge into habitat restoration: implications for meeting forest restoration challenges. Journal of Ethnobiology and Ethnomedicine, 19(1), 19, Article 33.

Heinimann, H. R. (2010). A concept in adaptive ecosystem management—an engineering perspective. Forest Ecology and Management, 259(4), 848-856.

Higuera, P. E., Metcalf, A. L., Miller, C., Buma, B., McWethy, D. B., Metcalf, E. C., Ratajczak, Z., Nelson, C. R., Chaffin, B. C., Stedman, R. C., McCaffrey, S., Schoennagel, T., Harvey, B. J., Hood, S. M., Schultz, C. A., Black, A. E., Campbell, D., Haggerty, J. H., Keane, R. E., Krawchuk, M. A., Kulig, J. C., Rafferty, R., and Virapongse, A. (2019). Integrating Subjective and Objective Dimensions of Resilience in Fire-Prone Landscapes. Bioscience, 69(5), 379-388.

Himes, A., Bauhus, J., Adhikari, S., Barik, S. K., Brown, H., Brunner, A., Burton, P. J., Coll, L., D'Amato, A. W., Diaci, J., Dorji, Y., Foli, E. G., Ganz, D. J., Hall, J. S., Keenan, R., Lu, Y. C., Messier, C., Munanura, I., Piotto, D., Seifert, T., Sheil, D., Shorohova, E., Sisay, K., Soto, D., Tanaka, H., Umunay, P., Velázquez-Martínez, A., and Puettmann, K. J. (2023). Forestry in the Face of Global Change: Results of a Global Survey of Professionals [Review]. Current Forestry Reports, 9(6), 473-489.

Hlásny, T., König, L., Krokene, P., Lindner, M., Montagné-Huck, C., Müller, J., Qin, H., Raffa, K. F., Schelhaas, M. J., Svoboda, M., Viiri, H., and Seidl, R. (2021). Bark Beetle Outbreaks in Europe: State of Knowledge and Ways Forward for Management. Current Forestry Reports, 7(3), 138-165.

Holl, K. D.and Aide, T. M. (2011). When and where to actively restore ecosystems? Forest Ecology and Management, 261(10), 1558-1563.

Holland, J. D. (2021). Longhorned Beetle Functional Diversity n Response to Biomass Harvesting. Environmental Entomology, 50(6), 1370-1377.

Holm, S. O. (2015). A Management Strategy for Multiple Ecosystem Services in Boreal Forests. Journal of Sustainable Forestry, 34(4), 358-379.

Hostert, P., Kuemmerle, T., Prishchepov, A., Sieber, A., Lambin, E. F., and Radeloff, V. C. (2011). Rapid land use change after socio-economic disturbances: the collapse of the Soviet Union versus Chernobyl. Environmental Research Letters, 6(4), 8, Article 045201.

Huang, B. W., Zha, R. B., Chen, S. F., Zha, X., and Jiang, X. X. (2023). Fuzzy evaluation of ecological vulnerability based on the SRP-SES method and analysis of multiple decision-making attitudes based on OWA operators: A case of Fujian Province, China. Ecological Indicators, 153, 17, Article 110432.

Huang, W. J., Wang, W., Cao, M., Fu, G., Xia, J. Y., Wang, Z. X., and Li, J. S. (2021). Local climate and biodiversity affect the stability of China's grasslands response to drought. Science of the Total Environment, 768, 12, Article 145482.

Huntington, H. P., Trainor, S. F., Natcher, D. C., Huntington, O. H., DeWilde, L., and Chapin, F. S. (2006). The significance of context in community-based research: Understanding discussions about wildfire in Huslia, Alaska [; Proceedings Paper]. Ecology and Society, 11(1), 12, Article 40.

Hurley, P. T.and Emery, M. R. (2018). Locating provisioning ecosystem services in urban forests: Forageable woody species in New York City, USA. Landscape and Urban Planning, 170, 266-275.

Huuskonen, S., Domisch, T., Finér, L., Hantula, J., Hynynen, J., Matala, J., Miina, J., Neuvonen, S., Nevalainen, S., Niemistö, P., Nikula, A., Piri, T., Siitonen, J., Smolander, A., Tonteri, T., Uotila, K., and Viiri, H. (2021). What is the potential for replacing monocultures with mixed-species stands to enhance ecosystem services in boreal forests in Fennoscandia? Forest Ecology and Management, 479, 21, Article 118558.

Ilunga, E. I. W., Mahy, G., Piqueray, J., Séleck, M., Shutcha, M. N., Meerts, P., and Faucon, M. P. (2015). Plant functional traits as a promising tool for the ecological restoration of degraded tropical metal-rich habitats and revegetation of metal-rich bare soils: A case study in copper vegetation of Katanga, DRC. Ecological Engineering, 82, 214-221.

Imbert, D. (2018). Hurricane disturbance and forest dynamics in east Caribbean mangroves. Ecosphere, 9(7), 13, Article e02231.

Isbell, F., Craven, D., Connolly, J., Loreau, M., Schmid, B., Beierkuhnlein, C., Bezemer, T. M., Bonin, C., Bruelheide, H., de Luca, E., Ebeling, A., Griffin, J. N., Guo, Q. F., Hautier, Y., Hector, A., Jentsch, A., Kreyling, J., Lanta, V., Manning, P., Meyer, S. T., Mori, A. S., Naeem, S., Niklaus, P. A., Polley, H. W., Reich, P. B., Roscher, C., Seabloom, E. W., Smith, M. D., Thakur, M. P., Tilman, D., Tracy, B. F., van der Putten, W. H., van Ruijven, J., Weigelt, A., Weisser, W. W., Wilsey, B., and Eisenhauer, N. (2015). Biodiversity increases the resistance of ecosystem productivity to climate extremes. Nature, 526(7574), 574-U263.

Islam, M. A., Paull, D. J., Griffin, A. L., and Murshed, S. (2020). Assessing ecosystem resilience to a tropical cyclone based on ecosystem service supply proficiency using geospatial techniques and social responses in coastal Bangladesh. International Journal of Disaster Risk Reduction, 49, 17, Article 101667.

Jackson, C. R., Webster, J. R., Knoepp, J. D., Elliott, K. J., Emanuel, R. E., Caldwell, P. V., and Miniat, C. F. (2018). Unexpected ecological advances made possible by long-term data: A Coweeta example. Wiley Interdisciplinary Reviews-Water, 5(2), 11, Article e1273.

Jackson, L. E., Pascual, U., and Hodgkin, T. (2007). Utilizing and conserving agrobiodiversity in agricultural landscapes [; Proceedings Paper]. Agriculture Ecosystems & Environment, 121(3), 196-210.

Jansson, Å.and Polasky, S. (2010). Quantifying Biodiversity for Building Resilience for Food Security in Urban Landscapes: Getting Down to Business. Ecology and Society, 15(3), 16, Article 20.

Jeffers, E. S., Nogué, S., and Willis, K. J. (2015). The role of palaeoecological records in assessing ecosystem services [Review]. Quaternary Science Reviews, 112, 17-32.

Jensen, D. A.and Svenning, J. C. (2021). Population ecology and dynamics of a remnant natural population of European yew, Taxus baccata, in a lowland temperate forest - implications for use in reforestation. Nordic Journal of Botany, 39(5), 14, Article e03167.

Jenssen, M., Nickel, S., and Schröder, W. (2021). Methodology for classifying the ecosystem integrity of forests in Germany using quantified indicators. Environmental Sciences Europe, 33(1), 28, Article 46.

Jiao, W. J., Yang, X., and Li, Y. W. (2024). Traditional knowledge's impact on soil and water conservation in mountain agricultural systems: A case study of Shexian Dryland stone terraced System, China. Ecological Indicators, 159, 15, Article 111742.

Jiren, T. S., Hanspach, J., Schultner, J., Fischer, J., Bergsten, A., Senbeta, F., Hylander, K., and Dorresteijn, I. (2020). Reconciling food security and biodiversity conservation: participatory scenario planning in southwestern Ethiopia. Ecology and Society, 25(3), 30, Article 24.

Johansson, K. E., Axelsson, R., Kimanzu, N., Sassi, S. O., Bwana, E., and Otsyina, R. (2013). The Pattern and Process of Adoption and Scaling up: Variation in Project Outcome Reveals the Importance of Multilevel Collaboration in Agroforestry Development. Sustainability, 5(12), 5195-5224.

Johnson, A., Clavijo, A. E., Hamar, G., Head, D. A., Thoms, A., Price, W., Lapke, A., Crotteau, J., Cerveny, L. K., Wilmer, H., Petershoare, L., Cook, A., and Reid, S. (2021). Wood Products for Cultural Uses: Sustaining Native Resilience and Vital Lifeways in Southeast Alaska, USA. Forests, 12(1), 26, Article 90.

Johnson, K. A., Dana, G., Jordan, N. R., Draeger, K. J., Kapuscinski, A., Olabisi, L. K. S., and Reich, P. B. (2012). Using Participatory Scenarios to Stimulate Social Learning for Collaborative Sustainable Development. Ecology and Society, 17(2), 22, Article 9.

Jones, A. G., Cridge, A., Fraser, S., Holt, L., Klinger, S., McGregor, K. F., Paul, T., Payn, T., Scott, M. B., Yao, R. T., and Dickinson, Y. (2023). Transitional forestry in New Zealand: re-evaluating the design and management of forest systems through the lens of forest purpose. Biological Reviews, 98(4), 1003-1015.

Jones, H. P., Jones, P. C., Barbier, E. B., Blackburn, R. C., Benayas, J. M. R., Holl, K. D., McCrackin, M., Meli, P., Montoya, D., and Mateos, D. M. (2018). Restoration and repair of Earth's damaged ecosystems. Proceedings of the Royal Society B-Biological Sciences, 285(1873), 8, Article 20172577.

Kabisch, N., Kraemer, R., Masztalerz, O., Hemmerling, J., Püffel, C., and Haase, D. (2021). Impact of summer heat on urban park visitation, perceived health and ecosystem service appreciation. Urban Forestry & Urban Greening, 60, 9, Article 127058.

Kahle, H. P., Hansen, J., and Spiecker, H. (2003, Jun 25-27). A moving target: Forest growth in a changing environment - the role of long-term dynamics.European Forest Institute Proceedings [Towards the sustainable use of europe's forests - forest ecosystem and landscape research: Scientific challenges and opportunities]. International Symposium on Towards the Sustainable Use of Europes Forests, Tours, FRANCE.

Karasmanaki, E., Mallinis, G., Mitsopoulos, I., Karteris, A., Chrysafis, I., Bakaloudis, D., Kokkoris, I. P., Maris, F., Arianoutsou, M., Goldammer, J. G., Rego, F., Vallejo, V. R., and Tsantopoulos, G. (2023). Proposing a Governance Model for Environmental Crises. Land, 12(3), 23, Article 597.

Kelly, C., Ferrara, A., Wilson, G. A., Ripullone, F., Nolè, A., Harmer, N., and Salvati, L. (2015). Community resilience and land degradation in forest and shrubland socio-ecological systems: Evidence from Gorgoglione, Basilicata, Italy. Land Use Policy, 46, 11-20.

Kern, C. C., Schwarzmann, J., Kabrick, J., Gerndt, K., Boyden, S., and Stanovick, J. S. (2019). Mounds facilitate regeneration of light-seeded and browse-sensitive tree species after moderate-severity wind disturbance. Forest Ecology and Management, 437, 139-147.

Keys, P. W., Collins, P. M., Chaplin-Kramer, R., and Wang-Erlandsson, L. (2024). Atmospheric water recycling an essential feature of critical natural asset stewardship. Global Sustainability, 7, 12, Article e2.

Kibria, A. M. G., Costanza, R., Gasparatos, A., and Soto, J. (2022). A composite human wellbeing index for ecosystem-dependent communities: A case study in the Sundarbans, Bangladesh. Ecosystem Services, 53, 13, Article 101389.

Klein, T., Zeppel, M. J. B., Anderegg, W. R. L., Bloemen, J., De Kauwe, M. G., Hudson, P., Ruehr, N. K., Powell, T. L., von Arx, G., and Nardini, A. (2018). Xylem embolism refilling and resilience against drought-induced mortality in woody plants: processes and trade-offs. Ecological Research, 33(5), 839-855.

Kleinschroth, F.and Healey, J. R. (2017). Impacts of logging roads on tropical forests [Review]. Biotropica, 49(5), 620-635.

Leal, L. C., Andersen, A. N., and Leal, I. R. (2014). Anthropogenic disturbance reduces seed-dispersal services for myrmecochorous plants in the Brazilian Caatinga. Oecologia, 174(1), 173-181.

Lee, E.and Krasny, M. E. (2015). The role of social learning for social-ecological systems in Korean village groves restoration. Ecology and Society, 20(1), 12, Article 42.

Li, H., Speer, J. H., and Thapa, I. (2022). Analyzing Resilience in the Greater Yellowstone Ecosystem after the 1988 Wildfire in the Western U.S. Using Remote Sensing and Soil Database. Land, 11(8), 19, Article 1172.

Liao, L. W., Ma, E. P., Long, H. L., and Peng, X. J. (2023). Land Use Transition and Its Ecosystem Resilience Response in China during 1990-2020. Land, 12(1), 19, Article 141.

Lichtenberg, S., Huber-Sannwald, E., Reyes-Agüero, J. A., Anhuf, D., and Nehren, U. (2022). Pau-brasil and string instrument bows telecouple nature, art, and heritage. Ecology and Society, 27(1), 59, Article 32.

Liu, J. G., Cui, W. H., Tian, Z., and Jia, J. L. (2021). Theory of stepwise ecological restoration [Review]. Chinese Science Bulletin-Chinese, 66(9), 1014-1025.

Liu, J. Y., Colloff, M. J., and Freudenberger, D. (2022). The Effects of Rehabilitation Treatments on Landscape Function Within a Softwood Plantation After Fire: Implications for Catchment Management. Forest Science, 68(2), 184-192.

Long, J. W., Lake, F. K., and Goode, R. W. (2021). The importance of Indigenous cultural burning in forested regions of the Pacific West, USA. Forest Ecology and Management, 500, 18, Article 119597.

Lugo-Carvajal, A., Holmgren, M., Zuanon, J., and van der Sleen, P. (2023). Fish on Fire: Shifts in Amazonian fish communities after floodplain forest fires [; Early Access]. Journal of Applied Ecology, 10.

Ma, X. B., Zhang, J. H., Wang, P. J., Zhou, L. Y., and Sun, Y. (2023a). Estimating the nonlinear response of landscape patterns to ecological resilience using a random forest algorithm: Evidence from the Yangtze River Delta. Ecological Indicators, 153, 14, Article 110409.

MacCord, P. L.and Begossi, A. (2006). Dietary changes over time in a Caicara community from the Brazilian Atlantic forest. Ecology and Society, 11(2), 13, Article 38.

MacLean, D. A.and Clark, K. L. (2021). Mixedwood management positively affects forest health during insect infestations in eastern North America. Canadian Journal of Forest Research, 51(7), 910-920.

Maebe, L., Dufrêne, M., Claessens, H., Maréchal, K., Ligot, G., and Messier, C. (2023). The Navigate framework: How the ecosystem services and resilience concepts can help us navigate in the current crises. Ecosystem Services, 64, 20, Article 101570.

Mahjoubi, I., Bossenbroek, L., Berger, E., and Frör, O. (2022). Analyzing Stakeholder Perceptions of Water Ecosystem Services to Enhance Resilience in the Middle Draa Valley, Southern Morocco. Sustainability, 14(8), 17, Article 4765.

Mallick, M., Singh, P. K., and Pandey, R. (2024). Harvesting resilience: Tribal home-gardens as socio-ecological solutions for climate change adaptation and sustainable development in a protected area. Journal of Cleaner Production, 445, 13, Article 141174.

Mansourian, S., Parrotta, J., Balaji, P., Bellwood-Howard, I., Bhasme, S., Bixler, R. P., Boedhihartono, A. K., Carmenta, R., Jedd, T., de Jong, W., Lake, F. K., Latawiec, A., Lippe, M., Rai, N. D., Sayer, J., Van Dexter, K., Vira, B., Visseren-Hamakers, I., Wyborn, C., and Yang, A. (2020). Putting the pieces together: Integration for forest landscape restoration implementation [Review]. Land Degradation & Development, 31(4), 419-429.

Manzano, S., Julier, A. C. M., Dirk, C. J., Razafimanantsoa, A. H., Samuels, I., Petersen, H., Gell, P., Hoffman, M. T., and Gillson, L. (2020). Using the past to manage the future: the role of palaeoecological and long-term data in ecological restoration. Restoration Ecology, 28(6), 1335-1342.

Marchand, L., Castagneyrol, B., Jiménez, J. J., Benayas, J. M. R., Benot, M. L., Martínez-Ruiz, C., Alday, J. G., Jaunatre, R., Dutoit, T., Buisson, E., Mench, M., Alard, D., Corcket, E., and Comin, F. (2021). Conceptual and methodological issues in

estimating the success of ecological restoration. Ecological Indicators, 123, 7, Article 107362.

Martínez, D.and García, D. (2017). Role of Avian Seed Dispersers in Tree Recruitment in Woodland Pastures. Ecosystems, 20(3), 616-629.

Marull, J., Font, C., Tello, E., Fullana, N., Domene, E., Pons, M., and Galán, E. (2016). Towards an energy-landscape integrated analysis? Exploring the links between socio-metabolic disturbance and landscape ecology performance (Mallorca, Spain, 1956-2011). Landscape Ecology, 31(2), 317-336.

Matasov, V., Vasenev, V., Matasov, D., Dvornikov, Y., Filyushkina, A., Bubalo, M., Nakhaev, M., and Konstantinova, A. (2023). COVID-19 pandemic changes the recreational use of Moscow parks in space and time: Outcomes from crowd-sourcing and machine learning. Urban Forestry & Urban Greening, 83, 13, Article 127911.

Mathes, K. C., Ju, Y., Kleinke, C., Oldfield, C., Bohrer, G., Bond-Lamberty, B., Vogel, C. S., Dorheim, K., and Gough, C. M. (2021). A multidimensional stability framework enhances interpretation and comparison of carbon cycling response to disturbance. Ecosphere, 12(11), 12, Article e03800.

Matsushita, K., Taki, H., Yamane, F., and Asano, K. (2018). SHADOW VALUE OF ECOSYSTEM RESILIENCE IN COMPLEX NATURAL LAND AS A WILD POLLINATOR HABITAT. American Journal of Agricultural Economics, 100(3), 829-843.

McCarthy, L. J.and Russo, A. (2023). Exploring the role of nature-based typologies and stewardship schemes in enhancing urban green spaces: Citizen perceptions of landscape design scenarios and ecosystem services. Journal of Environmental Management, 346, 10, Article 118944.

McKenna, P., Erskine, P. D., Glenn, V., and Doley, D. (2019). Response of open woodland and grassland mine site rehabilitation to fire disturbance on engineered landforms. Ecological Engineering, 133, 98-108.

McMillen, H., Campbell, L. K., Svendsen, E. S., and Reynolds, R. (2016). Recognizing Stewardship Practices as Indicators of Social Resilience: In Living Memorials and in a Community Garden. Sustainability, 8(8), 26, Article 775.

Meacham, M., Queiroz, C., Norström, A. V., and Peterson, G. D. (2016). Social-ecological drivers of multiple ecosystem services: what variables explain patterns of ecosystem services across the Norrstrom drainage basin? Ecology and Society, 21(1), 25, Article 14.

Medeiros, N. F., Ordóñez-Parra, C. A., Buisson, E., and Silveira, F. A. O. (2024). Systematic review of field research reveals critical shortfalls for restoration of tropical grassy biomes [Review]. Journal of Applied Ecology, 61(6), 1174-1186.

Medland, S. J., Shaker, R. R., Forsythe, K. W., Mackay, B. R., and Rybarczyk, G. (2020). A multi-Criteria Wetland Suitability Index for Restoration across Ontario's Mixedwood Plains. Sustainability, 12(23), 21, Article 9953.

Mekbeb, E. T., Lilieholm, R. J., Blahna, D. J., and Kruger, L. E. (2009, Jul 08-10). Resource use, dependence and vulnerability: community-resource linkages on Alaska's Tongass National Forest.WIT Transactions on Ecology and the Environment [Ecosystems and sustainable development vii]. 7th International Conference on Ecosystems and Sustainable Development, Chianciano Terme, ITALY.

Meli, P., Holl, K. D., Benayas, J. M. R., Jones, H. P., Jones, P. C., Montoya, D., and Mateos, D. M. (2017). A global review of past land use, climate, and active vs. passive restoration effects on forest recovery. Plos One, 12(2), 17, Article e0171368.

Meng, Y. Y., Liu, X. N., Wang, Z., Ding, C., and Zhu, L. H. (2021). How can spatial structural metrics improve the accuracy of forest disturbance and recovery detection using dense Landsat time series? Ecological Indicators, 132, 12, Article 108336.

Merschel, A. G., Beedlow, P. A., Shaw, D. C., Woodruff, D. R., Lee, E. H., Cline, S. P., Comeleo, R. L., Hagmann, R. K., and Reilly, M. J. (2021). An ecological perspective on living with fire in ponderosa pine forests of Oregon and Washington: Resistance, gone but not forgotten [Review]. Trees Forests and People, 4, 19, Article 100074.

Messier, C., Bauhus, J., Doyon, F., Maure, F., Sousa-Silva, R., Noler, P., Mina, M., Aquilué, N., Fortin, M. J., and Puettmann, K. (2019). The functional complex network approach to foster forest resilience to global changes. Forest Ecosystems, 6, 16, Article 21.

Messier, C., Bauhus, J., Sousa-Silva, R., Auge, H., Baeten, L., Barsoum, N., Bruelheide, H., Caldwell, B., Cavender-Bares, J., Dhiedt, E., Eisenhauer, N., Ganade, G., Gravel, D., Guillemot, J., Hall, J. S., Hector, A., Hérault, B., Jactel, H., Koricheva, J., Kreft, H., Mereu, S., Muys, B., Nock, C. A., Paquette, A., Parker, J. D., Perring, M. P., Ponette, Q., Potvin, C., Reich, P. B., Scherer-Lorenzen, M., Schnabel, F., Verheyen, K., Weih, M., Wollni, M., and Zemp, D. C. (2022). For the sake of resilience and multifunctionality, let's diversify planted forests! Conservation Letters, 15(1), 8, Article e12829.

Meyfroid, P., Chowdhury, R. R., de Bremond, A., Ellis, E. C., Erb, K. H., Filatova, T., Garrett, R. D., Grove, J. M., Heinimann, A., Kuemmerle, T., Kull, C. A., Lambin, E. F., Landon, Y., de Warow, Y. L., Messerli, P., Müller, D., Nielsen, J. O., Peterson, G. D., Garcia, V. R., Schlüter, M., Turner, B. L., and Verburg, P. H. (2018). Middle-range theories of land system change. Global Environmental Change-Human and Policy Dimensions, 53, 52-67.

Miller, A. M.and Davidson-Hunt, I. (2013). Agency and Resilience: Teachings of Pikangikum First Nation Elders, Northwestern Ontario. Ecology and Society, 18(3), 15, Article 9.

Mina, M., Messier, C., Duveneck, M. J., Fortin, M. J., and Aquilué, N. (2022). Managing for the unexpected: Building resilient forest landscapes to cope with global change. Global Change Biology, 28(14), 4323-4341.

Minayeva, T. Y., Filippov, I., Tysiachniouk, M. S., Markina, A., Kiselev, S. B., Lapshina, E. D., and Sirin, A. A. (2021). Connecting biodiversity and human dimensions through

ecosystem services: The Numto Nature Park in West Siberia. Ambio, 50(11), 2009-2021.

Mirauda, D., De Donato, R., and Santandrea, G. (2022). Proposed improvement of coastal habitat resilience: The case study of Pantano forest of Policoro in southern Italy. Frontiers in Marine Science, 9, 18, Article 891251.

Mirza, M. U., Richter, A., van Nes, E. H., and Scheffer, M. (2019). Technology driven inequality leads to poverty and resource depletion. Ecological Economics, 160, 215-226.

Moloise, S. D., Matamanda, A. R., and Bhanye, J. (2024). Traditional ecological knowledge and practices for ecosystem conservation and management: the case of savanna ecosystem services in Limpopo, South Africa. International Journal of Sustainable Development and World Ecology, 31(1), 29-42.

Monroy-Sais, S., García-Frapolli, E., Mora, F., Skutsch, M., Casas, A., Gerritsen, P. R. W., Cohen-Salgado, D., and Ugartechea-Salmerón, O. (2020). Unraveling households' natural resource management strategies: a case study in Jalisco, Mexico. Ecosystems and People, 16(1), 175-187.

Montoya, E., Matthews-Bird, F., Brooks, S. J., and Gosling, W. D. (2021). Forests protect aquatic communities from detrimental impact by volcanic deposits in the tropical Andes (Ecuador). Regional Environmental Change, 21(2), 14, Article 53.

Moreno-Fernández, D., Zavala, M. A., Madrigal-González, J., and Seijo, F. (2021). Resilience as a Moving Target: An Evaluation of Last Century Management Strategies in a Dry-Edge Maritime Pine Ecosystem. Forests, 12(9), 16, Article 1151.

Mori, A. S., Lertzman, K. P., and Gustafsson, L. (2017). Biodiversity and ecosystem services in forest ecosystems: a research agenda for applied forest ecology [Review]. Journal of Applied Ecology, 54(1), 12-27.

Moritsch, M. M., Byrd, K. B., Davis, M., Good, A., Drexler, J. Z., Morris, J. T., Woo, I., Windham-Myers, L., Grossman, E., Nakai, G., Poppe, K. L., and Rybczyk, J. M. (2022). Can Coastal Habitats Rise to the Challenge? Resilience of Estuarine Habitats, Carbon Accumulation, and Economic Value to Sea-Level Rise in a Puget Sound Estuary. Estuaries and Coasts, 45(8), 2293-2309.

Morris, J. L., Cottrell, S., Fettig, C. J., Hansen, W. D., Sherriff, R. L., Carter, V. A., Clear, J. L., Clement, J., DeRose, J., Hicke, J. A., Higuera, P. E., Mattor, K. M., Seddon, A. W. R., Seppä, H. T., Stednick, J. D., and Seybold, S. J. (2017). Managing bark beetle impacts on ecosystems and society: priority questions to motivate future research [Review]. Journal of Applied Ecology, 54(3), 750-760.

Müller, F., Bergmann, M., Dannowski, R., Dippner, J. W., Gnauck, A., Haase, P., Jochimsen, M. C., Kasprzak, P., Kröncke, I., Kümmerlin, R., Küster, M., Lischeid, G., Meesenburg, H., Merz, C., Millat, G., Müller, J., Padisák, J., Schimming, C. G., Schubert, H., Schult, M., Selmeczy, G., Shatwell, T., Stoll, S., Schwabe, M., Soltwedel, T., Straile, D., and Theuerkauf, M. (2016). Assessing resilience in long-term ecological data sets. Ecological Indicators, 65, 10-43.

Nevzati, F., Veldi, M., Kuelvik, M., and Bell, S. (2023). Analysis of Landscape Character Assessment and Cultural Ecosystem Services Evaluation Frameworks for Peri-Urban Landscape Planning: A Case Study of Harku Municipality, Estonia. Land, 12(10), 19, Article 1825.

Newton, A. C. (2011). Social-ecological Resilience and Biodiversity Conservation in a 900-year-old Protected Area. Ecology and Society, 16(4), 23, Article 13.

Nikinmaa, L., Lindner, M., Cantarello, E., Gardiner, B., Jacobsen, J. B., Jump, A. S., Parra, C., Plieninger, T., Schuck, A., Seidl, R., Timberlake, T., Waring, K., Winkel, G., and Muys, B. (2023). A balancing act: Principles, criteria and indicator framework to operationalize social-ecological resilience of forests. Journal of Environmental Management, 331, 14, Article 117039.

Nikinmaa, L., Lindner, M., Cantarello, E., Jump, A. S., Seidl, R., Winkel, G., and Muys, B. (2020). Reviewing the Use of Resilience Concepts in Forest Sciences. Current Forestry Reports, 6(2), 61-80.

Nikodemus, A., Hájek, M., Ndeinoma, A., and Purwestri, R. C. (2022). Forest Ecosystem Services-Based Adaptation Actions Supported by the National Policy on Climate Change for Namibia: Effectiveness, Indicators, and Challenges. Forests, 13(11), 21, Article 1965.

Nykvist, B.and von Heland, J. (2014). Social-ecological memory as a source of general and specified resilience. Ecology and Society, 19(2), 12, Article 47.

O'Donnell, S., Nguyen, T. M. H., Stimpson, C., Holmes, R., Kahlert, T., Hill, E., Vo, T., and Rabett, R. (2020). Holocene development and human use of mangroves and limestone forest at an ancient honglagoon in the Trang An karst, Ninh Binh, Vietnam [Review]. Quaternary Science Reviews, 242, 22, Article 106416.

O'Gorman, C. J., Bentley, L. P., McKay, C., Purser, M., and Everly, K. M. (2022). Examining abiotic and biotic factors influencing specimen black oaks (Quercus kelloggii) in northern California to reimplement traditional ecological knowledge and promote ecosystem resilience post-wildfire. Ecology and Society, 27(2), 12, Article 19.

Ocak, S. (2016). Transhumance in Central Anatolia: A Resilient Interdependence Between Biological and Cultural Diversity. Journal of Agricultural & Environmental Ethics, 29(3), 439-453.

Ocak, S., Ogun, S., and Yilmaz, O. (2016). CREATING RESILIENCE FOR TRANSHUMANT AND SMALL FARM SYSTEMS - TURKISH AND ROMANIAN PARADIGMS. Scientific Papers-Series D-Animal Science, 59, 228-232.

Ordonez-Barona, C., Wolf, K., Kowalski, J. M., Kendal, D., Byrne, J. A., and Conway, T. M. (2022). Diversity in public perceptions of urban forests and urban trees: A critical review [Review]. Landscape and Urban Planning, 226, 14, Article 104466.

Ordóñez, C. (2019). POLYCENTRIC GOVERNANCE IN NATURE-BASED SOLUTIONS: INSIGHTS FROM MELBOURNE URBAN FOREST MANAGERS. Landscape Architecture Frontiers, 7(3), 46-61.

Ortega, M., Pascual, S., Elena-Rosselló, R., and Rescia, A. J. (2020). Land-use and spatial resilience changes in the Spanish olive socio-ecological landscape. Applied Geography, 117, 10, Article 102171.

Osland, M. J., Enwright, N., and Stagg, C. L. (2014). Freshwater availability and coastal wetland foundation species: ecological transitions along a rainfall gradient. Ecology, 95(10), 2789-2802.

Oxbrough, A., Irwin, S., Wilson, M., and O'Halloran, J. (2014). Mechanisms and predictors of ecological change in managed A selection of papers from the second international conference on biodiversity in forest ecosystems and landscapes. Forest Ecology and Management, 321, 1-4.

Padovezi, A., Secco, L., Adams, C., and Chazdon, R. L. (2022). Bridging Social Innovation with Forest and Landscape Restoration. Environmental Policy and Governance, 32(6), 520-531.

Pandey, H. P., Aryal, K., Aryal, S., and Maraseni, T. N. (2023). Understanding local ecosystem dynamics in three provinces of the lowlands of Nepal. Science of the Total Environment, 867, 12, Article 161501.

Paraskevopoulou, A. T., Nektarios, P. A., and Kotsiris, G. (2019). Post-fire attitudes and perceptions of people towards the landscape character and development in the rural Peloponnese, a case study of the traditional village of Leontari, Arcadia, Greece. Journal of Environmental Management, 241, 567-574.

Pardos, M., del Río, M., Pretzsch, H., Jactel, H., Bielak, K., Bravo, F., Brazaitis, G., Defossez, E., Engel, M., Godvod, K., Jacobs, K., Jansone, L., Jansons, A., Morin, X., Nothdurft, A., Oreti, L., Ponette, Q., Pach, M., Riofrío, J., Ruíz-Peinado, R., Tomao, A., Uhl, E., and Calama, R. (2021). The greater resilience of mixed forests to drought mainly depends on their composition: Analysis along a climate gradient across Europe. Forest Ecology and Management, 481, 15, Article 118687.

Pawson, S. M., Brin, A., Brockerhoff, E. G., Lamb, D., Payn, T. W., Paquette, A., and Parrotta, J. A. (2013). Plantation forests, climate change and biodiversity. Biodiversity and Conservation, 22(5), 1203-1227.

Pecchi, M., Marchi, M., Moriondo, M., Forzieri, G., Ammoniaci, M., Bernetti, I., Bindi, M., and Chirici, G. (2020). Potential Impact of Climate Change on the Forest Coverage and the Spatial Distribution of 19 Key Forest Tree Species in Italy under RCP4.5 IPCC Trajectory for 2050s. Forests, 11(9), 19, Article 934.

Pedroza-Arceo, N. M., Weber, N., and Ortega-Argueta, A. (2022). A Knowledge Review on Integrated Landscape Approaches. Forests, 13(2), 24, Article 312.

Penaluna, B. E., Olson, D. H., Flitcroft, R. L., Weber, M. A., Bellmore, J. R., Wondzell, S. M., Dunham, J. B., Johnson, S. L., and Reeves, G. H. (2017). Aquatic biodiversity in

forests: a weak link in ecosystem services resilience. Biodiversity and Conservation, 26(13), 3125-3155.

Perring, M. P., Standish, R. J., Price, J. N., Craig, M. D., Erickson, T. E., Ruthrof, K. X., Whiteley, A. S., Valentine, L. E., and Hobbs, R. J. (2015). Advances in restoration ecology: rising to the challenges of the coming decades. Ecosphere, 6(8), 25, Article 131.

Pinho, P., Casanelles-Abella, J., Luz, A. C., Kubicka, A. M., Branquinho, C., Laanisto, L., Neuenkamp, L., Ortí, M. A., Obrist, M. K., Deguines, N., Tryjanowski, P., Samson, R., Niinemets, Ü., and Moretti, M. (2021). Research agenda on biodiversity and ecosystem functions and services in European cities. Basic and Applied Ecology, 53, 124-133.

Piponiot, C., Rödig, E., Putz, F. E., Rutishauser, E., Sist, P., Ascarrunz, N., Blanc, L., Derroire, G., Descroix, L., Guedes, M. C., Coronado, E. H., Huth, A., Kanashiro, M., Licona, J. C., Mazzei, L., d'Oliveira, M. V. N., Peña-Claros, M., Rodney, K., Shenkin, A., de Souza, C. R., Vidal, E., West, T. A. P., Worter, V., and Hérault, B. (2019). Can timber provision from Amazonian production forests be sustainable? Environmental Research Letters, 14(6), 10, Article 064014.

Plieninger, T., Hartel, T., Martín-López, B., Beaufoy, G., Bergmeier, E., Kirby, K., Montero, M. J., Moreno, G., Oteros-Rozas, E., and Van Uytvanck, J. (2015). Wood-pastures of Europe: Geographic coverage, social-ecological values, conservation management, and policy implications. Biological Conservation, 190, 70-79.

Plieninger, T.and Huntsinger, L. (2018). Complex Rangeland Systems: Integrated Social-Ecological Approaches to Silvopastoralism. Rangeland Ecology & Management, 71(5), 519-525.

Pomara, L. Y.and Lee, D. C. (2021). The Role of Regional Ecological Assessment in Quantifying Ecosystem Services for Forest Management [Review]. Land, 10(7), 21, Article 725.

Rafflegeau, S., Gosme, M., Barkaoui, K., Garcia, L., Allinne, C., Deheuvels, O., Grimaldi, J., Jagoret, P., Lauri, P. E., Merot, A., Metay, A., Reyes, F., Saj, S., Curry, G. N., and Justes, E. (2023). The ESSU concept for designing, modeling and auditing ecosystem service provision in intercropping and agroforestry systems. A review [Review]. Agronomy for Sustainable Development, 43(4), 24, Article 43.

Raj, A.and Sharma, L. K. (2023). Spatial E-PSR modelling for ecological sensitivity assessment for arid rangeland resilience and management. Ecological Modelling, 478, 14, Article 110283.

Ramdzan, K. N. M., Moss, P. T., Heijnis, H., Harrison, M. E., and Yulianti, N. (2022). Application of Palaeoecological and Geochemical Proxies in the Context of Tropical Peatland Degradation and Restoration: A Review for Southeast Asia [Review]. Wetlands, 42(7), 18, Article 95.

Rammer, W.and Seidl, R. (2015). Coupling human and natural systems: Simulating adaptive management agents in dynamically changing forest landscapes. Global Environmental Change-Human and Policy Dimensions, 35, 475-485.

Rani, N.and Sangwan, S. (2022). Advantages of Ecosystem Services to Human Being. Current Journal of Applied Science and Technology, 41(17), 19-24.

Rastetter, E. B., Ohman, M. D., Elliott, K. J., Rehage, J. S., Rivera-Monroy, V. H., Boucek, R. E., Castañeda-Moya, E., Danielson, T. M., Gough, L., Groffman, P. M., Jackson, C. R., Miniat, C. F., and Shaver, G. R. (2021). Time lags: insights from the US Long Term Ecological Research Network. Ecosphere, 12(5), 25, Article e03431.

Ratner, B. D., Meinzen-Dick, R., Hellin, J., Mapedza, E., Unruh, J., Veening, W., Haglund, E., May, C., and Bruch, C. (2017). Addressing conflict through collective action in natural resource management. International Journal of the Commons, 11(2), 877-906.

Raudsepp-Hearne, C.and Peterson, G. D. (2016). Scale and ecosystem services: how do observation, management, and analysis shift with scale-lessons from Quebec. Ecology and Society, 21(3), 35, Article 16.

Ray, D., Berlin, M., Alia, R., Sanchez, L., Hynynen, J., González-Martinez, S., and Bastien, C. (2022). Transformative changes in tree breeding for resilient forest restoration [Review]. Frontiers in Forests and Global Change, 5, 17, Article 1005761.

Reid, J. L., Wilson, S. J., Bloomfield, G. S., Cattau, M. E., Fagan, M. E., Holl, K. D., and Zahawi, R. A. (2017). HOW LONG DO RESTORED ECOSYSTEMS PERSIST? [; Proceedings Paper]. Annals of the Missouri Botanical Garden, 102(2), 258-265.

Reid, R. S., Fernández-Giménez, M. E., and Galvin, K. A. (2014). Dynamics and Resilience of Rangelands and Pastoral Peoples Around the Globe. In A. Gadgil & D. M. Liverman (Eds.), Annual Review of Environment and Resources, Vol 39 (Vol. 39, pp. 217-242). Annual Reviews. https://doi.org/10.1146/annurev-environ-020713-163329

Rist, L.and Moen, J. (2013). Sustainability in forest management and a new role for resilience thinking. Forest Ecology and Management, 310, 416-427.

Riva, M. J., Baeza, J., Bautista, S., Christoforou, M., Daliakopoulos, I. N., Hadjimitsis, D., Keizer, J. J., Liniger, H., Quaranta, G., Ribeiro, C., Salvia, R., Tsanis, I. K., Urgeghe, A. M., Valdecantos, A., and Schwilch, G. (2018). How does land management contribute to the resilience of Mediterranean forests and rangelands? A participatory assessment. Land Degradation & Development, 29(10), 3721-3735.

Rives, F., Antona, M., and Aubert, S. (2012). Social-ecological Functions and Vulnerability Framework to Analyze Forest Policy Reforms. Ecology and Society, 17(4), 18, Article 21.

Rockwell, C. A., Crow, A., Guimaraes, E. R., Recinos, E., and La Belle, D. (2022). Species Richness, Stem Density, and Canopy in Food Forests: Contributions to Ecosystem Services in an Urban Environment. Urban Planning, 7(2), 139-154.

Rodríguez-Rodríguez, J. C., Fenton, N. J., Kembel, S. W., Mestre, E., Jean, M., and Bergeron, Y. (2023). Drivers of contrasting boreal understory vegetation in coniferous and broadleaf deciduous alternative states. Ecological Monographs, 93(3), 24.

Rodriguez-Solorzano, C. (2014). Unintended outcomes of farmers' adaptation to climate variability: deforestation and conservation in Calakmul and Maya biosphere reserves. Ecology and Society, 19(2), 12, Article 53.

Romagnoli, F., Masiero, M., and Secco, L. (2022). Windstorm Impacts on Forest-Related Socio-Ecological Systems: An Analysis from a Socio-Economic and Institutional Perspective [Review]. Forests, 13(6), 31, Article 939.

Romolini, M., Bixler, R. P., and Grove, J. M. (2016). A Social-Ecological Framework for Urban Stewardship Network Research to Promote Sustainable and Resilient Cities. Sustainability, 8(9), 15, Article 956.

Rosenfield, M. F., Jakovac, C. C., Vieira, D. L. M., Poorter, L., Brancalion, P. H. S., Vieira, I. C. G., de Almeida, D. R. A., Massoca, P., Schietti, J., Albernaz, A. L. M., Ferreira, M. J., and Mesquita, R. C. G. (2023). Ecological integrity of tropical secondary forests: concepts and indicators. Biological Reviews, 98(2), 662-676.

Ruiz-Mallén, I.and Corbera, E. (2013). Community-Based Conservation and Traditional Ecological Knowledge: Implications for Social-Ecological Resilience. Ecology and Society, 18(4), 19, Article 12.

Salomón, R. L., Peters, R. L., Zweifel, R., Sass-Klaassen, U. G. W., Stegehuis, A. I., Smiljanic, M., Poyatos, R., Babst, F., Cienciala, E., Fonti, P., Lerink, B. J. W., Lindner, M., Martinez-Vilalta, J., Mencuccini, M., Nabuurs, G. J., van der Maaten, E., von Arx, G., Bär, A., Akhmetzyanov, L., Balanzategui, D., Bellan, M., Bendix, J., Berveiller, D., Blazenec, M., Cada, V., Carraro, V., Cecchini, S., Chan, T., Conedera, M., Delpierre, N., Delzon, S., Ditmarová, L., Dolezal, J., Dufrêne, E., Edvardsson, J., Ehekircher, S., Forner, A., Frouz, J., Ganthaler, A., Gryc, V., Güney, A., Heinrich, I., Hentschel, R., Janda, P., Jezík, M., Kahle, H. P., Knüsel, S., Krejza, J., Kuberski, L., Kucera, J., Lebourgeois, F., Mikolás, M., Matula, R., Mayr, S., Oberhuber, W., Obojes, N., Osborne, B., Paljakka, T., Plichta, R., Rabbel, I., Rathgeber, C. B. K., Salmon, Y., Saunders, M., Scharnweber, T., Sitková, Z., Stangler, D. F., Sterenczak, K., Stojanovic, M., Strelcová, K., Svetlík, J., Svoboda, M., Tobin, B., Trotsiuk, V., Urban, J., Valladares, F., Vavrcík, H., Vejpustková, M., Walthert, L., Wilmking, M., Zin, E., Zou, J. L., and Steppe, K. (2022). The 2018 European heatwave led to stem dehydration but not to consistent growth reductions in forests. Nature Communications, 13(1), 11, Article 28.

Samani, D., Bosak, K., and Halvorson, S. J. (2023). Community-Centered Sustainable Ecotourism Planning in the Bossou Forest Reserve, Guinea, West Africa. Sustainability, 15(5), 24, Article 4615.

Santiago, L. E., Forero-Montaña, J., Melendez-Ackerman, E. J., Gould, W. A., and Zimmerman, J. K. (2022). Social Acceptability of a Sustainable Forestry Industry in Puerto Rico: Views of Private, Public, and Non-Profit Sectors. Forests, 13(4), 16, Article 576.

Sapkota, P., Keenan, R. J., and Ojha, H. R. (2019). Co-evolving dynamics in the social-ecological system of community forestry-prospects for ecosystem-based adaptation in the Middle Hills of Nepal. Regional Environmental Change, 19(1), 179-192.

Sari, R. R., Priyadarshini, R., Rozendaal, D. M. A., Saputra, D. D., Hairiah, K., and van Noordwijk, M. (2023). Tree diversity and social-ecological resilience of agroforestry after volcanic ash deposition in Indonesia. Sustainability Science, 18(6), 2735-2753.

Sarkki, S., Ficko, A., Wielgolaski, F. E., Abraham, E. M., Bratanova-Doncheva, S., Grunewald, K., Hofgaard, A., Holtmeier, F. K., Kyriazopoulos, A. P., Broll, G., Nijnik, M., and Sutinen, M. L. (2017). Assessing the resilient provision of ecosystem services by social-ecological systems: introduction and theory. Climate Research, 73(1-2), 7-15.

Sarkki, S., Jokinen, M., Nijnik, M., Zahvoyska, L., Abraham, E. M., Alados, C. L., Bellamy, C., Bratanova-Dontcheva, S., Grunewald, K., Kollar, J., Krajcí, J., Kyriazopoulos, A. P., La Porta, N., Monteiro, A. T., Munoz-Rojas, J., Parpan, T., Sing, L., Smith, M., Sutinen, M. L., Tolvanen, A., and Zhyla, T. (2017). Social equity in governance of ecosystem services: synthesis from European treeline areas. Climate Research, 73(1-2), 31-44.

Schaffer-Smith, D., Myint, S. W., Muenich, R. L., Tong, D. Q., and DeMeester, J. E. (2020). Repeated Hurricanes Reveal Risks and Opportunities for Social-Ecological Resilience to Flooding and Water Quality Problems. Environmental Science & Technology, 54(12), 7194-7204.

Schick, A., Porembski, S., Hobson, P. R., and Ibisch, P. L. (2019). Classification of key ecological attributes and stresses of biodiversity for ecosystem-based conservation assessments and management. Ecological Complexity, 38, 98-111.

Schirpke, U., Alizinger, A., Leitinger, G., and Tasser, E. (2019). Change from agricultural to touristic use: Effects on the aesthetic value of landscapes over the last 150 years. Landscape and Urban Planning, 187, 23-35.

Schirpke, U., Tasser, E., Leitinger, G., and Tappeiner, U. (2022). Using the Ecosystem Services Concept to Assess Transformation of Agricultural Landscapes in the European Alps. Land, 11(1), 14, Article 49.

Schoennagel, T., Balch, J. K., Brenkert-Smith, H., Dennison, P. E., Harvey, B. J., Krawchuk, M. A., Mietkiewicz, N., Morgan, P., Moritz, M. A., Rasker, R., Turner, M. G., and Whitlock, C. (2017). Adapt to more wildfire in western North American forests as climate changes. Proceedings of the National Academy of Sciences of the United States of America, 114(18), 4582-4590.

Schoenrock, K. M., Chan, K. M., O'Callaghan, T., O'Callaghan, R., Golden, A., Krueger-Hadfield, S. A., and Power, A. M. (2020). A review of subtidal kelp forests in Ireland: From first descriptions to new habitat monitoring techniques [Review]. Ecology and Evolution, 10(13), 6819-6832.

Seddon, A. W. R., Macias-Fauria, M., Long, P. R., Benz, D., and Willis, K. J. (2016). Sensitivity of global terrestrial ecosystems to climate variability. Nature, 531(7593), 229-+.

Seddon, N., Chausson, A., Berry, P., Girardin, C. A. J., Smith, A., and Turner, B. (2020). Understanding the value and limits of nature-based solutions to climate change and other global challenges [Review]. Philosophical Transactions of the Royal Society B-Biological Sciences, 375(1794), 12, Article 20190120.

Seidl, R., Spies, T. A., Peterson, D. L., Stephens, S. L., and Hicke, J. A. (2016). Searching for resilience: addressing the impacts of changing disturbance regimes on forest ecosystem services. Journal of Applied Ecology, 53(1), 120-129.

Semeraro, T., Buccolieri, R., Vergine, M., De Bellis, L., Luvisi, A., Emmanuel, R., and Marwan, N. (2021). Analysis of Olive Grove Destruction by Xylella fastidiosa Bacterium on the Land Surface Temperature in Salento Detected Using Satellite Images. Forests, 12(9), 18, Article 1266.

Semeraro, T., Gatto, E., Buccolieri, R., Catanzaro, V., De Bellis, L., Cotrozzi, L., Lorenzini, G., Vergine, M., and Luvisi, A. (2021). How Ecosystem Services Can Strengthen the Regeneration Policies for Monumental Olive Groves Destroyed by Xylella fastidiosa Bacterium in a Peri-Urban Area. Sustainability, 13(16), 22, Article 8778.

Semeraro, T., Luvisi, A., Lillo, A. O., Aretano, R., Buccolieri, R., and Marwan, N. (2020). Recurrence Analysis of Vegetation Indices for Highlighting the Ecosystem Response to Drought Events: An Application to the Amazon Forest. Remote Sensing, 12(6), 20, Article 907.

Senf, C.and Seidl, R. (2022). Post-disturbance canopy recovery and the resilience of Europe's forests. Global Ecology and Biogeography, 31(1), 25-36.

Sheppard, J. P., Reckziegel, R. B., Borrass, L., Chirwa, P. W., Cuaranhua, C. J., Hassler, S. K., Hoffmeister, S., Kestel, F., Maier, R., Mälicke, M., Morhart, C., Ndlovu, N. P., Veste, M., Funk, R., Lang, F., Seifert, T., Toit, B. D., and Kahle, H. P. (2020). Agroforestry: An Appropriate and Sustainable Response to a Changing Climate in Southern Africa? [Review]. Sustainability, 12(17), 31, Article 6796.

Shuman, J. K., Balch, J. K., Barnes, R. T., Higuera, P. E., Roos, C. I., Schwilk, D. W., Stavros, E. N., Banerjee, T., Bela, M. M., Bendix, J., Bertolino, S., Bililign, S., Bladon, K. D., Brando, P., Breidenthal, R. E., Buma, B., Calhoun, D., Carvalho, L. M. V., Cattau, M. E., Cawley, K. M., Chandra, S., Chipman, M. L., Cobian-Iñiguez, J., Conlisk, E., Coop, J. D., Cullen, A., Davis, K. T., Dayalu, A., De Sales, F., Dolman, M., Ellsworth, L. M., Franklin, S., Guiterman, C. H., Hamilton, M., Hanan, E. J., Hansen, W. D., Hantson, S., Harvey, B. J., Holz, A., Huang, T., Hurteau, M. D., Ilangakoon, N. T., Jennings, M., Jones, C., Klimaszewski-Patterson, A., Kobziar, L. N., Kominoski, J., Kosovic, B., Krawchuk, M. A., Laris, P., Leonard, J., Loria-Salazar, S. M., Lucash, M., Mahmoud, H., Margolis, E., Maxwell, T., McCarty, J. L., McWethy, D. B., Meyer, R. S., Miesel, J. R., Moser, W. K., Nagy, R. C., Niyogi, D., Palmer, H. M., Pellegrini, A., Poulter, B., Robertson, K., Rocha, A. V., Sadegh, M., Santos, F., Scordo, F., Sexton, J. O., Sharma, A. S., Smith, A. M. S., Soja, A. J., Still, C., Swetnam, T., Syphard, A. D., Tingley, M. W., Tohidi, A., Trugman, A. T.,

Turetsky, M., Varner, J. M., Wang, Y. H., Whitman, T., Yelenik, S., and Zhang, X. (2022). Reimagine fire science for the anthropocene. Pnas Nexus, 1(3), 14, Article pgac115.

Shumi, G., Loos, J., and Fischer, J. (2024). Applying social-ecological system resilience principles to the context of woody vegetation management in smallholder farming landscapes of the Global South [Review]. Ecosystems and People, 20(1), 17, Article 2339222.

Smith, D. A. E., Si, X. F., Smith, Y. C. E., and Downs, C. T. (2018). Seasonal variation in avian diversity and tolerance by migratory forest specialists of the patch-isolation gradient across a fragmented forest system. Biodiversity and Conservation, 27(14), 3707-3727.

Soubry, I., Doan, T., Chu, T., and Guo, X. L. (2021). A Systematic Review on the Integration of Remote Sensing and GIS to Forest and Grassland Ecosystem Health Attributes, Indicators, and Measures [Review]. Remote Sensing, 13(16), 30, Article 3262.

Souza-Alonso, P., Saiz, G., García, R. A., Pauchard, A., Ferreira, A., and Merino, A. (2022). Post-fire ecological restoration in Latin American forest ecosystems: Insights and lessons from the last two decades. Forest Ecology and Management, 509, 20, Article 120083.

Specht, M. J., Santos, B. A., Marshall, N., Melo, F. P. L., Leal, I. R., Tabarelli, M., and Baldauf, C. (2019). Socioeconomic differences among resident, users and neighbour populations of a protected area in the Brazilian dry forest. Journal of Environmental Management, 232, 607-614.

Steenberg, J. W. N., Duinker, P. N., and Nitoslawski, S. A. (2019). Ecosystem-based management revisited: Updating the concepts for urban forests [Review]. Landscape and Urban Planning, 186, 24-35.

Stevens-Rumann, C. S., Kemp, K. B., Higuera, P. E., Harvey, B. J., Rother, M. T., Donato, D. C., Morgan, P., and Veblen, T. T. (2018). Evidence for declining forest resilience to wildfires under climate change. Ecology Letters, 21(2), 243-252.

Suarez-Pardo, A., Villegas-Palacio, C., and Berrouet, L. (2022). Resilience in Agroecosystems: An Index Based on a Socioecological Systems Approach. Weather Climate and Society, 14(2), 425-438.

Sui, H. X., Liu, C., Ye, C. M., Xu, X. L., and Sui, T. B. (2023). Analysis of ecosystem resilience in Jiuzhaigou Valley Scenic Area under the effect of geohazards. Frontiers in Earth Science, 10, 14, Article 1053327.

Sun, Z. Y., Ren, H., Schaefer, V., Lu, H. F., Wang, J., Li, L. J., and Liu, N. (2013). Quantifying ecological memory during forest succession: A case study from lower subtropical forest ecosystems in South China. Ecological Indicators, 34, 192-203.

Sutherland, I. J., Copes-Gerbitz, K., Parrott, L., and Rhemtulla, J. M. (2023). Dynamics in the landscape ecology of institutions: lags, legacies, and feedbacks drive path-dependency of forest landscapes in British Columbia, Canada 1858-2020. Landscape Ecology, 38(12), 4325-4341.

Sutherland, I. J., Van Vianen, J., Rowland, D., Palomo, I., Pascual, U., Mathys, A., Narulita, S., and Sunderland, T. (2023). Use, value, and desire: ecosystem services under agricultural intensification in a changing landscape in West Kalimantan (Indonesia). Regional Environmental Change, 23(4), 16, Article 148.

Tagg, N., Kuenbou, J. K., Laméris, D. W., Meigang, F. M. K., Kekeunou, S., Epanda, M. A., Dupain, J., Mbohli, D., Redmond, I., and Willie, J. (2020). Long-term trends in wildlife community structure and functional diversity in a village hunting zone in southeast Cameroon. Biodiversity and Conservation, 29(2), 571-590.

Tagliari, M. M., Bogoni, J. A., Blanco, G. D., Cruz, A. P., and Peroni, N. (2023). Disrupting a socio-ecological system: could traditional ecological knowledge be the key to preserving the Araucaria Forest in Brazil under climate change? Climatic Change, 176(2), 20, Article 2.

Takahashi, S.and Liang, L. (2016). Roles of forests in food security based on case studies in Yunnan, China. International Forestry Review, 18(1), 123-132.

Tang, J. H., Xiong, K. N., Wang, Q., Chen, Y., and Wu, Q. L. (2023). Village ecosystem vulnerability in karst desertification control: evidence from South China Karst. Frontiers in Ecology and Evolution, 11, 14, Article 1126659.

Tanguay, L.and Bernard, S. (2020). Ecoagricultural landscapes in the dieng mountains of central Java; A study of their evolution and dynamics. Journal of Rural Studies, 77, 169-184.

Tanner, S. J., Escobedo, F. J., and Soto, J. R. (2021). Recognizing the insurance value of resilience: Evidence from a forest restoration policy in the southeastern US. Journal of Environmental Management, 289, 10, Article 112442.

Temperton, V. M., Buchmann, N., Buisson, E., Durigan, G., Kazmierczak, L., Perring, M. P., Dechoum, M. D., Veldman, J. W., and Overbeck, G. E. (2019). Step back from the forest and step up to the Bonn Challenge: how a broad ecological perspective can promote successful landscape restoration. Restoration Ecology, 27(4), 705-719.

Tidball, K. G. (2014). Seeing the forest for the trees: hybridity and social-ecological symbols, rituals and resilience in postdisaster contexts. Ecology and Society, 19(4), 14, Article 25.

Tidball, K. G., Krasny, M. E., Svendsen, E., Campbell, L., and Helphand, K. (2010). Stewardship, learning, and memory in disaster resilience. Environmental Education Research, 16(5-6), 591-609, Article Pii 928399893.

Townsend, P. A.and Masters, K. L. (2015). Lattice-work corridors for climate change: a conceptual framework for biodiversity conservation and social-ecological resilience in a tropical elevational gradient. Ecology and Society, 20(2), 11, Article 1.

Tscharntke, T., Clough, Y., Bhagwat, S. A., Buchori, D., Faust, H., Hertel, D., Hölscher, D., Juhrbandt, J., Kessler, M., Perfecto, I., Scherber, C., Schroth, G., Veldkamp, E., and Wanger, T. C. (2011). Multifunctional shade-tree management in tropical agroforestry landscapes - a review [Review]. Journal of Applied Ecology, 48(3), 619-629.

Tuffery, L., Davi, H., López-García, N., Rigolot, E., Jean, F., Stenger, A., and Lefèvre, F. (2021). Adaptive measures for mountain Mediterranean forest ecosystem services under climate and land cover change in the Mont-Ventoux regional nature park, France. Regional Environmental Change, 21(1), 14, Article 12.

Turner, B., Devisscher, T., Chabaneix, N., Woroniecki, S., Messier, C., and Seddon, N. (2022). The role of nature-based solutions in supporting social-ecological resilience for climate change adaptation. Annual Review of Environment and Resources, 47(1), 123-148.

Urgenson, L. S., Hagmann, R. K., Henck, A. C., Harrell, S., Hinckley, T. M., Shepler, S. J., Grub, B. L., and Chi, P. M. (2010). Social-ecological Resilience of a Nuosu Community-linked Watershed, Southwest Sichuan, China. Ecology and Society, 15(4), 23, Article Zz.

Urza, A. K., Weisberg, P. J., Chambers, J. C., Dhaemers, J. M., and Board, D. (2017). Post-fire vegetation response at the woodland-shrubland interface is mediated by the pre-fire community. Ecosphere, 8(6), 20, Article e01851.

Valente, D., Miglietta, P. P., Porrini, D., Pasimeni, M. R., Zurlini, G., and Petrosillo, I. (2019). A first analysis on the need to integrate ecological aspects into financial insurance. Ecological Modelling, 392, 117-127.

Valipour, A., Plieninger, T., Shakeri, Z., Ghazanfari, H., Namiranian, M., and Lexer, M. J. (2014). Traditional silvopastoral management and its effects on forest stand structure in northern Zagros, Iran. Forest Ecology and Management, 327, 221-230.

Vallet, A., Locatelli, B., Barnaud, C., Makowski, D., Conde, Y. Q., and Levrel, H. (2020). Power asymmetries in social networks of ecosystem services governance. Environmental Science & Policy, 114, 329-340.

van der Jagt, A. P. N., Smith, M., Ambrose-Oji, B., Konijnendijk, C. C., Giannico, V., Haase, D., Lafortezza, R., Nastran, M., Pintar, M., Zeleznikar, S., and Cvejic, R. (2019). Co-creating urban green infrastructure connecting people and nature: A guiding framework and approach. Journal of Environmental Management, 233, 757-767.

Vega, S. G. D., Heras, J. D., and Moya, D. (2016). Resilience of Mediterranean terrestrial ecosystems and fire severity in semiarid areas: Responses of Aleppo pine forests in the short, mid and long term. Science of the Total Environment, 573, 1171-1177.

Verkerk, P. J., Costanza, R., Hetemäki, L., Kubiszewski, I., Leskinen, P., Nabuurs, G. J., Potocnik, J., and Palahí, M. (2020). Climate-Smart Forestry: the missing link. Forest Policy and Economics, 115, 4, Article 102164.

Vignola, R., Locatelli, B., Martinez, C., and Imbach, P. (2009). Ecosystem-based adaptation to climate change: what role for policy-makers, society and scientists? Mitigation and Adaptation Strategies for Global Change, 14(8), 691-696.

von Heland, J.and Folke, C. (2014). A social contract with the ancestors-Culture and ecosystem services in southern Madagascar. Global Environmental Change-Human and Policy Dimensions, 24, 251-264.

Wagner, S., Nocentini, S., Huth, F., and Hoogstra-Klein, M. (2014). Forest Management Approaches for Coping with the Uncertainty of Climate Change: Trade-Offs in Service Provisioning and Adaptability. Ecology and Society, 19(1), 16, Article 32.

Waldron, K., Thiffault, N., Venier, L., Bognounou, F., Boucher, D., Campbell, E., Whitman, E., Brehaut, L., and Gauthier, S. (2023). A pan-Canadian assessment of empirical research on post-disturbance recovery in the Canadian Forest Service. Canadian Journal of Forest Research, 53(11), 823-838.

Walpole, E. H., Toman, E., Wilson, R. S., and Stidham, M. (2017). Shared visions, future challenges: a case study of three Collaborative Forest Landscape Restoration Program locations. Ecology and Society, 22(2), 15, Article 35.

Wang, T., Li, H. B., and Huang, Y. (2021). The complex ecological network's resilience of the Wuhan metropolitan area. Ecological Indicators, 130, 16, Article 108101.

Wayne, A. F., Maxwell, M. A., Ward, C. G., Vellios, C. V., Williams, M. R., and Pollock, K. H. (2016). The responses of a critically endangered mycophagous marsupial (Bettongia penicillata) to timber harvesting in a native eucalypt forest. Forest Ecology and Management, 363, 190-199.

Wellmann, T., Andersson, E., Knapp, S., Lausch, A., Palliwoda, J., Priess, J., Scheuer, S., and Haase, D. (2023). Reinforcing nature-based solutions through tools providing social-ecological-technological integration [Review]. Ambio, 52(3), 489-507.

Wells, G. J., Fisher, J., Jindal, R., and Ryan, C. M. (2020). Social as much as environmental: the drivers of tree biomass in smallholder forest landscape restoration programmes. Environmental Research Letters, 15(10), 13, Article 104008.

Williams, J. N., Safford, H. D., Enstice, N., Steel, Z. L., and Paulson, A. K. (2023). High-severity burned area and proportion exceed historic conditions in Sierra Nevada, California, and adjacent ranges. Ecosphere, 14(1), 17, Article e4397.

Winter, K. B., Lincoln, N. K., Berkes, F., Alegado, R. A., Kurashima, N., Frank, K. L., Pascua, P., Rii, Y. M., Reppun, F., Knapp, I. S. S., McClatchey, W. C., Ticktin, T., Smith, C., Franklin, E. C., Oleson, K., Price, M. R., McManus, M. A., Donahue, M. J., Rodgers, K. S., Bowen, B. W., Nelson, C. E., Thomas, B., Leong, J. A., Madin, E. M. P., Rivera, M. A. J., Falinski, K. A., Bremer, L. L., Deenik, J. L., Gon, S. M., Neilson, B., Okano, R., Olegario, A., Nyberg, B., Kawelo, A. H., Kotubetey, K., Kukea-Shultz, J. K., and Toonen, R. J. (2020). Ecomimicry in Indigenous resource management: optimizing ecosystem services to achieve resource abundance, with examples from Hawai'i. Ecology and Society, 25(2), 18, Article 26.

Winter, K. B., Ticktin, T., and Quazi, S. A. (2020). Biocultural restoration in Hawai'i also achieves core conservation goals. Ecology and Society, 25(1), 18, Article 26.

Wonkka, C. L., Twidwell, D., West, J. B., and Rogers, W. E. (2016). Shrubland resilience varies across soil types: implications for operationalizing resilience in ecological restoration. Ecological Applications, 26(1), 128-145.

Wood, C. M.and Jones, G. M. (2019). Framing management of social-ecological systems in terms of the cost of failure: the Sierra Nevada, USA as a case study. Environmental Research Letters, 14(10), 11, Article 105004.

Wu, G. J., Xu, G. B., Wang, B., Liu, X. H., Chen, T., and Kang, H. H. (2023). Post-drought moisture condition determines tree growth recovery after extreme drought events in the Tianshan Mountains, northwestern China. Ecological Indicators, 151, 9, Article 110275.

Wu, T.and Kim, Y. S. (2013). Pricing ecosystem resilience in frequent-fire ponderosa pine forests. Forest Policy and Economics, 27, 8-12.

Wyatt, K. H., Arkema, K. K., Wells-Moultrie, S., Silver, J. M., Lashley, B., Thomas, A., Kuiper, J. J., Guerry, A. D., and Ruckelshaus, M. (2021). Integrated and innovative scenario approaches for sustainable development planning in The Bahamas. Ecology and Society, 26(4), 18, Article 23.

Xie, Q. Y., Moore, C. E., Cleverly, J., Hall, C. C., Ding, Y. L., Ma, X. L., Leigh, A., and Huete, A. (2023). Land surface phenology indicators retrieved across diverse ecosystems using a modified threshold algorithm. Ecological Indicators, 147, 13, Article 110000.

Xu, C., Ke, Y. G., Zhou, W., Luo, W. T., Ma, W., Song, L., Smith, M. D., Hoover, D. L., Wilcox, K. R., Fu, W., Zhang, W. G., and Yu, Q. (2021). Resistance and resilience of a semi-arid grassland to multi-year extreme drought. Ecological Indicators, 131, 7, Article 108139.

Xu, H. Z., Peng, M., Pittock, J., and Xu, J. Y. (2021). Managing Rather Than Avoiding "Difficulties" in Building Landscape Resilience. Sustainability, 13(5), 24, Article 2629.

Yang, Q., Huang, Z., Wu, L., Guo, B. B., Liu, M. L., Xue, X. J., Li, X. Y., and Liu, X. N. (2024). Resilience changes of carbon stocks to quantify the long-term effects of ecological engineering projects in subtropical forests of China based on satellite-derived net ecosystem production time series and inventory data. Land Degradation & Development, 35(7), 2329-2344.

Yu, K. J. (2021). Rescuing Big Rivers Damaged in Industrial Civilization [Editorial Material]. Landscape Architecture Frontiers, 9(3), 4-7.

Zanini, A. M., Mayrinck, R. C., Vieira, S. A., de Camargo, P. B., and Rodrigues, R. R. (2021). The effect of ecological restoration methods on carbon stocks in the Brazilian Atlantic Forest. Forest Ecology and Management, 481, 11, Article 118734.

Zari, M. P. (2019). Devising Urban Biodiversity Habitat Provision Goals: Ecosystem Services Analysis. Forests, 10(5), 17, Article 391.

Zhou, X. P., Shen, D. S., and Gu, X. K. (2022). Influences of Land Policy on Urban Ecological Corridors Governance: A Case Study from Shanghai. International Journal of Environmental Research and Public Health, 19(15), 21, Article 9747.

Zinnert, J. C., Nippert, J. B., Rudgers, J. A., Pennings, S. C., González, G., Alber, M., Baer, S. G., Blair, J. M., Burd, A., Collins, S. L., Craft, C., Di Iorio, D., Dodds, W. K., Groffman, P. M., Herbert, E., Hladik, C., Li, F., Litvak, M. E., Newsome, S., O'Donnell, J., Pockman, W. T., Schalles, J., and Young, D. R. (2021). State changes: insights from the US Long Term Ecological Research Network. Ecosphere, 12(5), 29, Article e03433.

References

Ahmadi, A., Kerachian, R., Rahimi, R., and Skardi, M. J. E. (2019). Comparing and combining social network analysis and stakeholder analysis for natural resource governance. *Environmental Development*, *32*, 100451.

Ahmadi, A., Kerachian, R., Skardi, M. J. E., and Abdolhay, A. (2020). A stakeholder-based decision support system to manage water resources. *Journal of Hydrology*, *589*, 125138.

Albiero, A., Venegas-González, A., Botosso, P. C., Roig, F. A., Camargo, J. L. C., and Tomazello, M. (2019). What is the temporal extension of edge effects on tree growth dynamics? A dendrochronological approach model using *Scleronema micranthum* (Ducke) Ducke trees of a fragmented forest in the Central Amazon. *Ecological Indicators*, *101*, 133-142.

Alfaro-Sánchez, R., Jump, A. S., Pino, J., Díez-Nogales, O., and Espelta, J. M. (2019). Land use legacies drive higher growth, lower wood density and enhanced climatic sensitivity in recently established forests. *Agricultural and Forest Meteorology*, *276*, 11, Article 107630.

Anamaghi, S., Behboudian, M., Mahjouri, N., and Kerachian, R. (2023). A resilience-based framework for evaluating the carrying capacity of water and environmental resources under the climate change. *Science of the Total Environment*, *902*, 165986.

Anderegg, W. R. L., Trugman, A. T., Badgley, G., Konings, A. G., and Shaw, J. (2020). Divergent forest sensitivity to repeated extreme droughts. *Nature Climate Change*, *10*(12), 1091-U1019.

Arenas-Wong, R. A., Robles-Morúa, A., Bojórquez, A., Martínez-Yrízar, A., Yépez, E. A., and Alvarez-Yépiz, J. C. (2023). Climate-induced changes to provisioning ecosystem services in rural socioecosystems in Mexico. *Weather and Climate Extremes*, *41*, 13, Article 100583.

Asbjornsen, H., Hernandez-Santana, V., Liebman, M., Bayala, J., Chen, J., Helmers, M., Ong, C. K., and Schulte, L. A. (2014). Targeting perennial vegetation in agricultural landscapes for enhancing ecosystem services [Review]. *Renewable Agriculture and Food Systems*, *29*(2), 101-125.

Behboudian, M., Anamaghi, S., Kerachian, R., and Kalantari, Z. (2024). Comparison of three group decision-making frameworks for evaluating resilience time series of water resources systems under uncertainty. *Ecological Indicators*, *158*, 111269.

Behboudian, M., Anamaghi, S., Mahjouri, N., and Kerachian, R. (2023). Enhancing the resilience of ecosystem services under extreme events in socio-hydrological systems: a spatio-temporal analysis. *Journal of Cleaner Production*, *397*, 136437.

Beier, C. M., Patterson, T. M., and Chapin, F. S. (2008). Ecosystem services and emergent vulnerability in managed ecosystems: A geospatial decision-support tool. *Ecosystems*, *11*(6), 923-938.

Bryant, T., Waring, K., Meador, A. S., and Bradford, J. B. (2019). A Framework for Quantifying Resilience to Forest Disturbance. *Frontiers in Forests and Global Change*, *2*, 14, Article 56.

Cantarello, E., Newton, A. C., Martin, P. A., Evans, P. M., Gosal, A., and Lucash, M. S. (2017). Quantifying resilience of multiple ecosystem services and biodiversity in a temperate forest landscape. *Ecology and Evolution*, *7*(22), 9661-9675.

Chambers, J. C., Brown, J. L., Bradford, J. B., Board, D. I., Campbell, S. B., Clause, K. J., Hanberry, B., Schlaepfer, D. R., and Urza, A. K. (2023). New indicators of ecological resilience and invasion resistance to support prioritization and management in the sagebrush biome, United States. *Frontiers in Ecology and Evolution*, *10*, 17, Article 1009268.

Charnley, S., Spies, T. A., Barros, A. M. G., White, E. M., and Olsen, K. A. (2017). Diversity in forest management to reduce wildfire losses: implications for resilience. *Ecology and Society*, *22*(1), 25, Article 22.

Chazdon, R. L.and Guariguata, M. R. (2016). Natural regeneration as a tool for large-scale forest restoration in the tropics: prospects and challenges. *Biotropica*, *48*(6), 716-730.

Che, C. W., Xiao, S. C., Peng, X. M., Ding, A. J., and Su, J. R. (2023). Radial growth of Korshinsk peashrub and its response to drought in different sub-arid climate regions of northwest China. *Journal of Environmental Management*, *326*, 10, Article 116708.

Cotroneo, S. M., Jacobo, E. J., and Brassiolo, M. M. (2021). Degradation processes and adaptive strategies in communal forests of Argentine dry Chaco. Integrating stakeholder knowledge and perceptions. *Ecosystems and People*, *17*(1), 507-522.

Cottrell, S., Mattor, K. M., Morris, J. L., Fettig, C. J., McGrady, P., Maguire, D., James, P. M. A., Clear, J., Wurtzebach, Z., Wei, Y., Brunelle, A., Western, J., Maxwell, R., Rotar, M., Gallagher, L., and Roberts, R. (2020). Adaptive capacity in social-ecological systems: a framework for addressing bark beetle disturbances in natural resource management. *Sustainability Science*, *15*(2), 555-567.

Cowles, J., Templeton, L., Battles, J. J., Edmunds, P. J., Carpenter, R. C., Carpenter, S. R., Nelson, M. P., Cleavitt, N. L., Fahey, T. J., Groffman, P. M., Sullivan, J. H., Neel, M. C., Hansen, G. J. A., Hobbie, S., Holbrook, S. J., Kazanski, C. E., Seabloom, E. W., Schmitt, R. J., Stanley, E. H., Tepley, A. J., van Doorn, N. S., and Vander Zanden, J. M. (2021). Resilience: insights from the US LongTerm Ecological Research Network. *Ecosphere*, *12*(5), 31, Article e03434.

Danneyrolles, V., Valeria, O., Djerboua, I., Gauthier, S., and Bergeron, Y. (2020). How Initial Forest Cover, Site Characteristics and Fire Severity Drive the Dynamics of the Southern Boreal Forest. *Remote Sensing*, *12*(23), 11, Article 3957.

de la Barrera, F., Barraza, F., Favier, P., Ruiz, V., and Quense, J. (2018). Megafires in Chile 2017: Monitoring multiscale environmental impacts of burned ecosystems. *Science of the Total Environment*, *637*, 1526-1536.

de la Mora, G. D., Sánchez-Nupan, L. O., Castro-Torres, B., and Galicia, L. (2021). Sustainable Community Forest Management in Mexico: An Integrated Model of Three Socio-ecological Frameworks. *Environmental Management*, *68*(6), 900-913.

de Medeiros-Sarmento, P. S., Ferreira, L. V., and Gastauer, M. (2021). Natural regeneration triggers compositional and functional shifts in soil seed banks. *Science of the Total Environment*, *753*, 9, Article 141934.

Devisscher, T., Spies, J., and Griess, V. C. (2021). Time for change: Learning from community forests to enhance the resilience of multi-value forestry in British Columbia, Canada. *Land Use Policy*, *103*, 16, Article 105317.

Drillet, Z., Fung, T. K., Leong, R. A. T., Sachidhanandam, U., Edwards, P., and Richards, D. (2020). Urban Vegetation Types are Not Perceived Equally in Providing Ecosystem Services and Disservices. *Sustainability*, *12*(5), 14, Article 2076.

Dutra, D. J., Elmiro, M. A. T., and Ribeiro, S. M. C. (2022). Association between forest resources and water availability: temporal analysis of the Serra Azul stream sub-basin. *Anais Da Academia Brasileira De Ciencias*, *94*(3), 15, Article e20201289.

Enoki, T., Nakashizuka, T., Nakano, S., Miki, T., Lin, Y. P., Nakaoka, M., Mizumachi, E., and Shibata, H. (2014). Progress in the 21st century: a Roadmap for the Ecological Society of Japan. *Ecological Research*, *29*(3), 357-368.

Fang, O. Y.and Zhang, Q. B. (2019). Tree resilience to drought increases in the Tibetan Plateau. *Global Change Biology*, *25*(1), 245-253.

Ferrara, A., Kelly, C., Wilson, G. A., Nolè, A., Mancino, G., Bajocco, S., and Salvati, L. (2016). Shaping the role of 'fast' and 'slow' drivers of change in forest-shrubland socio-ecological systems. *Journal of Environmental Management*, *169*, 155-166.

Frazier, A. E., Renschler, C. S., and Miles, S. B. (2013). Evaluating post-disaster ecosystem resilience using MODIS GPP data. *International Journal of Applied Earth Observation and Geoinformation*, *21*, 43-52.

Frei, B., Queiroz, C., Chaplin-Kramer, B., Andersson, E., Renard, D., Rhemtulla, J. M., and Bennett, E. M. (2020). A brighter future: Complementary goals of diversity and multifunctionality to build resilient agricultural landscapes. *Global Food Security-Agriculture Policy Economics and Environment*, *26*, 11, Article 100407.

Freudenberger, L., Hobson, P. R., Schluck, M., and Ibisch, P. L. (2012). A global map of the functionality of terrestrial ecosystems. *Ecological Complexity*, *12*, 13-22.

Gauthier, S., Bernier, P., Kuuluvainen, T., Shvidenko, A. Z., and Schepaschenko, D. G. (2015). Boreal forest health and global change [Review]. *Science*, *349*(6250), 819-822.

Gazol, A., Valeriano, C., Cantero, A., Vergarechea, M., and Camarero, J. J. (2022). Douglas Fir Growth Is Constrained by Drought: Delineating the Climatic Limits of Timber Species under Seasonally Dry Conditions. *Forests*, *13*(11), 16, Article 1796.

Guz, J.and Kulakowski, D. (2020). Forests in the Anthropocene. *Annals of the American Association of Geographers*, *111*(3), 869-879.

Haberstroh, S.and Werner, C. (2022). The role of species interactions for forest resilience to drought [Review]. *Plant Biology*, *24*(7), 1098-1107.

Hlásny, T., König, L., Krokene, P., Lindner, M., Montagné-Huck, C., Müller, J., Qin, H., Raffa, K. F., Schelhaas, M. J., Svoboda, M., Viiri, H., and Seidl, R. (2021). Bark Beetle Outbreaks in Europe: State of Knowledge and Ways Forward for Management. *Current Forestry Reports*, *7*(3), 138-165.

Huang, W. J., Wang, W., Cao, M., Fu, G., Xia, J. Y., Wang, Z. X., and Li, J. S. (2021). Local climate and biodiversity affect the stability of China's grasslands response to drought. *Science of the Total Environment*, *768*, 12, Article 145482.

Ilunga, E. I. W., Mahy, G., Piqueray, J., Séleck, M., Shutcha, M. N., Meerts, P., and Faucon, M. P. (2015). Plant functional traits as a promising tool for the ecological restoration of degraded tropical metal-rich habitats and revegetation of metal-rich bare soils: A case study in copper vegetation of Katanga, DRC. *Ecological Engineering*, *82*, 214-221.

Islam, M. A., Paull, D. J., Griffin, A. L., and Murshed, S. (2020). Assessing ecosystem resilience to a tropical cyclone based on ecosystem service supply proficiency using geospatial techniques and social responses in coastal Bangladesh. *International Journal of Disaster Risk Reduction*, *49*, 17, Article 101667.

Jackson, L. E., Pascual, U., and Hodgkin, T. (2007). Utilizing and conserving agrobiodiversity in agricultural landscapes [; Proceedings Paper]. *Agriculture Ecosystems & Environment*, *121*(3), 196-210.

Jansson, Å.and Polasky, S. (2010). Quantifying Biodiversity for Building Resilience for Food Security in Urban Landscapes: Getting Down to Business. *Ecology and Society*, *15*(3), 16, Article 20.

Johnson, A., Clavijo, A. E., Hamar, G., Head, D. A., Thoms, A., Price, W., Lapke, A., Crotteau, J., Cerveny, L. K., Wilmer, H., Petershoare, L., Cook, A., and Reid, S. (2021). Wood Products for Cultural Uses: Sustaining Native Resilience and Vital Lifeways in Southeast Alaska, USA. *Forests*, *12*(1), 26, Article 90.

Kelly, C., Ferrara, A., Wilson, G. A., Ripullone, F., Nolè, A., Harmer, N., and Salvati, L. (2015). Community resilience and land degradation in forest and shrubland socio-ecological systems: Evidence from Gorgoglione, Basilicata, Italy. *Land Use Policy*, *46*, 11-20.

Klein, T., Zeppel, M. J. B., Anderegg, W. R. L., Bloemen, J., De Kauwe, M. G., Hudson, P., Ruehr, N. K., Powell, T. L., von Arx, G., and Nardini, A. (2018). Xylem embolism refilling and resilience against drought-induced mortality in woody plants: processes and trade-offs. *Ecological Research*, *33*(5), 839-855.

Kleinschroth, F.and Healey, J. R. (2017). Impacts of logging roads on tropical forests [Review]. *Biotropica*, *49*(5), 620-635.

Li, H., Speer, J. H., and Thapa, I. (2022). Analyzing Resilience in the Greater Yellowstone Ecosystem after the 1988 Wildfire in the Western U.S. Using Remote Sensing and Soil Database. *Land*, *11*(8), 19, Article 1172.

Liao, L. W., Ma, E. P., Long, H. L., and Peng, X. J. (2023). Land Use Transition and Its Ecosystem Resilience Response in China during 1990-2020. *Land*, *12*(1), 19, Article 141.

Ma, X. B., Zhang, J. H., Wang, P. J., Zhou, L. Y., and Sun, Y. (2023a). Estimating the nonlinear response of landscape patterns to ecological resilience using a random forest algorithm: Evidence from the Yangtze River Delta. *Ecological Indicators*, *153*, 14, Article 110409.

Mansourian, S., Parrotta, J., Balaji, P., Bellwood-Howard, I., Bhasme, S., Bixler, R. P., Boedhihartono, A. K., Carmenta, R., Jedd, T., de Jong, W., Lake, F. K., Latawiec, A., Lippe, M., Rai, N. D., Sayer, J., Van Dexter, K., Vira, B., Visseren-Hamakers, I., Wyborn, C., and Yang, A. (2020). Putting the pieces together: Integration for forest landscape restoration implementation [Review]. *Land Degradation & Development*, *31*(4), 419-429.

Martínez, D.and García, D. (2017). Role of Avian Seed Dispersers in Tree Recruitment in Woodland Pastures. *Ecosystems*, *20*(3), 616-629.

McMillen, H., Campbell, L. K., Svendsen, E. S., and Reynolds, R. (2016). Recognizing Stewardship Practices as Indicators of Social Resilience: In Living Memorials and in a Community Garden. *Sustainability*, *8*(8), 26, Article 775.

Meacham, M., Queiroz, C., Norström, A. V., and Peterson, G. D. (2016). Social-ecological drivers of multiple ecosystem services: what variables explain patterns of ecosystem services across the Norrstrom drainage basin? *Ecology and Society*, *21*(1), 25, Article 14.

Mina, M., Messier, C., Duveneck, M. J., Fortin, M. J., and Aquilué, N. (2022). Managing for the unexpected: Building resilient forest landscapes to cope with global change. *Global Change Biology*, *28*(14), 4323-4341.

Moreno-Fernández, D., Zavala, M. A., Madrigal-González, J., and Seijo, F. (2021). Resilience as a Moving Target: An Evaluation of Last Century Management Strategies in a Dry-Edge Maritime Pine Ecosystem. *Forests*, *12*(9), 16, Article 1151.

Mori, A. S., Lertzman, K. P., and Gustafsson, L. (2017). Biodiversity and ecosystem services in forest ecosystems: a research agenda for applied forest ecology [Review]. *Journal of Applied Ecology*, *54*(1), 12-27.

Nevzati, F., Veldi, M., Kuelvik, M., and Bell, S. (2023). Analysis of Landscape Character Assessment and Cultural Ecosystem Services Evaluation Frameworks for Peri-Urban Landscape Planning: A Case Study of Harku Municipality, Estonia. *Land*, *12*(10), 19, Article 1825.

Newton, A. C. (2011). Social-ecological Resilience and Biodiversity Conservation in a 900-year-old Protected Area. *Ecology and Society*, *16*(4), 23, Article 13.

Nikinmaa, L., Lindner, M., Cantarello, E., Gardiner, B., Jacobsen, J. B., Jump, A. S., Parra, C., Plieninger, T., Schuck, A., Seidl, R., Timberlake, T., Waring, K., Winkel, G., and Muys, B. (2023). A balancing act: Principles, criteria and indicator framework to operationalize social-ecological resilience of forests. *Journal of Environmental Management*, *331*, 14, Article 117039.

Nikinmaa, L., Lindner, M., Cantarello, E., Jump, A. S., Seidl, R., Winkel, G., and Muys, B. (2020). Reviewing the Use of Resilience Concepts in Forest Sciences. *Current Forestry Reports*, *6*(2), 61-80.

Pardos, M., del Río, M., Pretzsch, H., Jactel, H., Bielak, K., Bravo, F., Brazaitis, G., Defossez, E., Engel, M., Godvod, K., Jacobs, K., Jansone, L., Jansons, A., Morin, X., Nothdurft, A., Oreti, L., Ponette, Q., Pach, M., Riofrío, J., Ruíz-Peinado, R., Tomao, A., Uhl, E., and Calama, R. (2021). The greater resilience of mixed forests to drought mainly depends on their composition: Analysis along a climate gradient across Europe. *Forest Ecology and Management*, *481*, 15, Article 118687.

Penaluna, B. E., Olson, D. H., Flitcroft, R. L., Weber, M. A., Bellmore, J. R., Wondzell, S. M., Dunham, J. B., Johnson, S. L., and Reeves, G. H. (2017). Aquatic biodiversity in forests: a weak link in ecosystem services resilience. *Biodiversity and Conservation*, *26*(13), 3125-3155.

Pomara, L. Y.and Lee, D. C. (2021). The Role of Regional Ecological Assessment in Quantifying Ecosystem Services for Forest Management [Review]. *Land*, *10*(7), 21, Article 725.

Raj, A.and Sharma, L. K. (2023). Spatial E-PSR modelling for ecological sensitivity assessment for arid rangeland resilience and management. *Ecological Modelling*, *478*, 14, Article 110283.

Rastetter, E. B., Ohman, M. D., Elliott, K. J., Rehage, J. S., Rivera-Monroy, V. H., Boucek, R. E., Castañeda-Moya, E., Danielson, T. M., Gough, L., Groffman, P. M., Jackson, C. R., Miniat, C. F., and Shaver, G. R. (2021). Time lags: insights from the US Long Term Ecological Research Network. *Ecosphere*, *12*(5), 25, Article e03431.

Reid, J. L., Wilson, S. J., Bloomfield, G. S., Cattau, M. E., Fagan, M. E., Holl, K. D., and Zahawi, R. A. (2017). HOW LONG DO RESTORED ECOSYSTEMS PERSIST? [; Proceedings Paper]. *Annals of the Missouri Botanical Garden*, *102*(2), 258-265.

Rosenfield, M. F., Jakovac, C. C., Vieira, D. L. M., Poorter, L., Brancalion, P. H. S., Vieira, I. C. G., de Almeida, D. R. A., Massoca, P., Schietti, J., Albernaz, A. L. M., Ferreira, M. J., and Mesquita, R. C. G. (2023). Ecological integrity of tropical secondary forests: concepts and indicators. *Biological Reviews*, *98*(2), 662-676.

Ruiz-Mallén, I.and Corbera, E. (2013). Community-Based Conservation and Traditional Ecological Knowledge: Implications for Social-Ecological Resilience. *Ecology and Society*, *18*(4), 19, Article 12.

Samani, D., Bosak, K., and Halvorson, S. J. (2023). Community-Centered Sustainable Ecotourism Planning in the Bossou Forest Reserve, Guinea, West Africa. *Sustainability*, *15*(5), 24, Article 4615.

Schoennagel, T., Balch, J. K., Brenkert-Smith, H., Dennison, P. E., Harvey, B. J., Krawchuk, M. A., Mietkiewicz, N., Morgan, P., Moritz, M. A., Rasker, R., Turner, M. G., and Whitlock, C. (2017). Adapt to more wildfire in western North American forests as climate changes. *Proceedings of the National Academy of Sciences of the United States of America*, *114*(18), 4582-4590.

Seddon, A. W. R., Macias-Fauria, M., Long, P. R., Benz, D., and Willis, K. J. (2016). Sensitivity of global terrestrial ecosystems to climate variability. *Nature*, *531*(7593), 229-+.

Seidl, R., Spies, T. A., Peterson, D. L., Stephens, S. L., and Hicke, J. A. (2016). Searching for resilience: addressing the impacts of changing disturbance regimes on forest ecosystem services. *Journal of Applied Ecology*, *53*(1), 120-129.

Semeraro, T., Luvisi, A., Lillo, A. O., Aretano, R., Buccolieri, R., and Marwan, N. (2020). Recurrence Analysis of Vegetation Indices for Highlighting the Ecosystem Response to Drought Events: An Application to the Amazon Forest. *Remote Sensing*, *12*(6), 20, Article 907.

Soubry, I., Doan, T., Chu, T., and Guo, X. L. (2021). A Systematic Review on the Integration of Remote Sensing and GIS to Forest and Grassland Ecosystem Health Attributes, Indicators, and Measures [Review]. *Remote Sensing*, *13*(16), 30, Article 3262.

Souza-Alonso, P., Saiz, G., García, R. A., Pauchard, A., Ferreira, A., and Merino, A. (2022). Post-fire ecological restoration in Latin American forest ecosystems: Insights and lessons from the last two decades. *Forest Ecology and Management*, *509*, 20, Article 120083.

Steenberg, J. W. N., Duinker, P. N., and Nitoslawski, S. A. (2019). Ecosystem-based management revisited: Updating the concepts for urban forests [Review]. *Landscape and Urban Planning*, *186*, 24-35.

Stevens-Rumann, C. S., Kemp, K. B., Higuera, P. E., Harvey, B. J., Rother, M. T., Donato, D. C., Morgan, P., and Veblen, T. T. (2018). Evidence for declining forest resilience to wildfires under climate change. *Ecology Letters*, *21*(2), 243-252.

Suarez-Pardo, A., Villegas-Palacio, C., and Berrouet, L. (2022). Resilience in Agroecosystems: An Index Based on a Socioecological Systems Approach. *Weather Climate and Society*, *14*(2), 425-438.

Sui, H. X., Liu, C., Ye, C. M., Xu, X. L., and Sui, T. B. (2023). Analysis of ecosystem resilience in Jiuzhaigou Valley Scenic Area under the effect of geohazards. *Frontiers in Earth Science*, *10*, 14, Article 1053327.

Sun, Z. Y., Ren, H., Schaefer, V., Lu, H. F., Wang, J., Li, L. J., and Liu, N. (2013). Quantifying ecological memory during forest succession: A case study from lower subtropical forest ecosystems in South China. *Ecological Indicators*, *34*, 192-203.

Takahashi, S.and Liang, L. (2016). Roles of forests in food security based on case studies in Yunnan, China. *International Forestry Review*, *18*(1), 123-132.

Tscharntke, T., Clough, Y., Bhagwat, S. A., Buchori, D., Faust, H., Hertel, D., Hölscher, D., Juhrbandt, J., Kessler, M., Perfecto, I., Scherber, C., Schroth, G., Veldkamp, E., and Wanger, T. C. (2011). Multifunctional shade-tree management in tropical agroforestry landscapes - a review [Review]. *Journal of Applied Ecology*, *48*(3), 619-629.

Turner, B., Devisscher, T., Chabaneix, N., Woroniecki, S., Messier, C., and Seddon, N. (2022). The role of nature-based solutions in supporting social-ecological resilience for climate change adaptation. *Annual Review of Environment and Resources*, *47*(1), 123-148.

Valipour, A., Plieninger, T., Shakeri, Z., Ghazanfari, H., Namiranian, M., and Lexer, M. J. (2014). Traditional silvopastoral management and its effects on forest stand structure in northern Zagros, Iran. *Forest Ecology and Management*, *327*, 221-230.

Vega, S. G. D., Heras, J. D., and Moya, D. (2016). Resilience of Mediterranean terrestrial ecosystems and fire severity in semiarid areas: Responses of Aleppo pine forests in the short, mid and long term. *Science of the Total Environment*, *573*, 1171-1177.

Waldron, K., Thiffault, N., Venier, L., Bognounou, F., Boucher, D., Campbell, E., Whitman, E., Brehaut, L., and Gauthier, S. (2023). A pan-Canadian assessment of empirical research on post-disturbance recovery in the Canadian Forest Service. *Canadian Journal of Forest Research*, *53*(11), 823-838.

Wang, T., Li, H. B., and Huang, Y. (2021). The complex ecological network's resilience of the Wuhan metropolitan area. *Ecological Indicators*, *130*, 16, Article 108101.

Wonkka, C. L., Twidwell, D., West, J. B., and Rogers, W. E. (2016). Shrubland resilience varies across soil types: implications for operationalizing resilience in ecological restoration. *Ecological Applications*, *26*(1), 128-145.

Wu, G. J., Xu, G. B., Wang, B., Liu, X. H., Chen, T., and Kang, H. H. (2023). Post-drought moisture condition determines tree growth recovery after extreme drought events in the Tianshan Mountains, northwestern China. *Ecological Indicators*, *151*, 9, Article 110275.

Wu, T.and Kim, Y. S. (2013). Pricing ecosystem resilience in frequent-fire ponderosa pine forests. *Forest Policy and Economics*, *27*, 8-12.

Yang, Q., Huang, Z., Wu, L., Guo, B. B., Liu, M. L., Xue, X. J., Li, X. Y., and Liu, X. N. (2024). Resilience changes of carbon stocks to quantify the long-term effects of ecological engineering projects in subtropical forests of China based on satellite-derived net ecosystem production time series and inventory data. *Land Degradation & Development*, *35*(7), 2329-2344.
